# Highly Accessible Computational Prediction and *In Vivo/In Vitro* Experimental Validation: Novel Synthetic Phenyl Ketone Derivatives as Promising Agents against NAFLD via Modulating Oxidoreductase Activity

**DOI:** 10.1155/2023/3782230

**Published:** 2023-01-09

**Authors:** Yanan Qiao, Huifang Deng, Lihua Liu, Shuran Liu, Luyao Ren, Chuandao Shi, Xi Chen, Lixia Guan, Weiran Liu, Zehua Li, Yunlan Li

**Affiliations:** ^1^School of Pharmaceutical Science, Shanxi Medical University, Taiyuan 030001, China; ^2^Department of Pharmacy, Second Hospital of Shanxi Medical University, Taiyuan, China; ^3^Department of Automation, Tsinghua University, Beijing 100080, China; ^4^School of Public Health, Shaanxi University of Chinese Medicine, Xi'an 712046, China

## Abstract

Nonalcoholic fatty liver disease (NAFLD) has reached epidemic proportions with no pharmacological treatment approved. Several highly accessible computational tools were employed to predict the activities of twelve novel compounds prior to actual chemical synthesis. We began our work by designing two or three hydroxyl groups appended to the phenyl ketone core, followed by prediction of drug-likeness and targets. Most predicted targets for each compound overlapped with NAFLD targets (≥80%). Enrichment analysis showed that these compounds might regulate oxidoreductase activity. Then, these compounds were synthesized and confirmed by IR, MS, ^1^H, and ^13^C NMR. Their cell viability demonstrated that twelve compounds exhibited appreciable potencies against NAFLD (EC_50_ values ≤ 13.5 *μ*M). Furthermore, the most potent compound **5f** effectively prevented NAFLD progression as evidenced by the change in histological features. **5f** significantly reduced total cholesterol and triglyceride levels *in vitro/in vivo*, and the effects of **5f** were significantly stronger than those of the control drug. The proteomic data showed that oxidoreductase activity was the most significantly enriched, and this finding was consistent with docking results. In summary, this validated presynthesis prediction approach was cost-saving and worthy of popularization. The novel synthetic phenyl ketone derivative **5f** holds great therapeutic potential by modulating oxidoreductase activity to counter NAFLD.

## 1. Introduction

In the current era of big data, computational methods have led to a more cost-, time-, and labor-efficient drug discovery process [1]. Among computational methods, cheminformatics has the advantage of simulating human targets and can overcome the shortages of animal experiments to some extent. Bioinformatics better understands biological/molecular behaviors and offers possible solutions for new drugs tailored to individuals, which is crucial to supporting precision medicine [2].

Nonalcoholic fatty liver disease (NAFLD) encompasses a disease spectrum, from simple steatosis to nonalcoholic steatohepatitis (NASH), fibrosis, and cirrhosis. The prevalence of NAFLD is estimated to be 25% globally and continues to increase [3]. Unfortunately, there is currently no drug officially approved for NAFLD treatment, resulting in a significant unmet medical need [4–7]. Hence, new effective NAFLD drugs are urgently needed. Phenyl ketone derivatives have received considerable interest because they exhibit multiple pharmacological activities such as hepatoprotective, anti-inflammatory [8], antimicrobial [9], antidiabetic [10], and anticancer [11]. From a hepatoprotective point of view, phenyl ketone derivatives demonstrate antioxidant [12] and antifibrotic potential [13].

**Figure 1:**
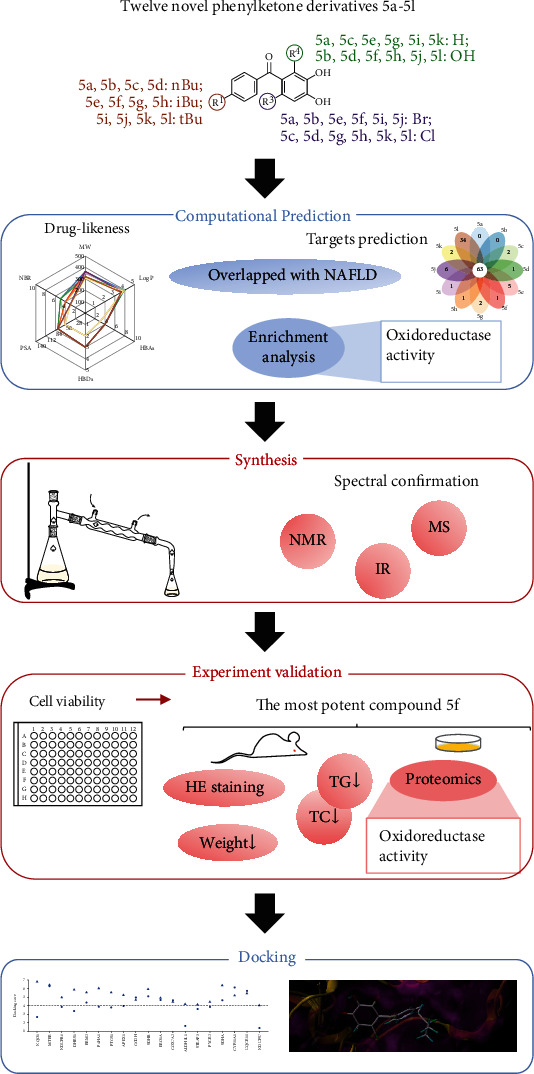
Workflow of this study. The contents within the blue box are performed on computers. The contents within the red box are traditional and more realistic experiments. nBu: n-butyl; iBu: isobutyl; tBu: tert-butyl.

To our knowledge, this was the first time to apply computational technologies to predict the drug-likeness and biological activities of novel phenyl ketone derivatives for treating NAFLD prior to chemical synthesis. The prediction results were in line with subsequent experiment results. The design of this study is shown in Figure 1. Firstly, building on the structure-activity relationship of our previously synthesized patented phenyl ketone derivatives (Chinese patent number 202010778813.5) for anti-inflammatory activities [14, 15], twelve novel phenyl ketone derivatives with a butyl group in ring A and a halogen group in ring B were designed. Computational strategies were applied to assess drug-likeness features and predict the targets of these compounds. For predicted targets, the overlapping percentages with NAFLD targets were calculated and Gene Ontology (GO) analysis was performed. Secondly, synthetic routes were designed to synthesize these twelve compounds. Mass spectroscopy (MS), infrared (IR), ^1^H, and ^13^C nuclear magnetic resonance (NMR) were used to confirm the structures. Thirdly, cell viability experiments were conducted to validate the computational prediction results initially. Another validation was made by evaluating the effects of the most potent compound **5f** on *in vitro/in vivo* NAFLD models. The proteomic study was performed to search for a mechanism by which **5f** intervention may be affecting NAFLD and to validate prediction results further. Finally, molecular docking was conducted to help understand the mechanism of **5f**.

## 2. Results and Discussion

### 2.1. Computational Prediction of Twelve Phenyl Ketone Derivatives

#### 2.1.1. Drug-Likeness Prediction

Preevaluation of drug-likeness is valuable during the early stage of drug discovery. Compounds with desirable bioavailability can be preselected in a time-efficient manner [16]. Compounds that do not go against any criteria of drug-likeness rules are more likely to become oral drugs [17]. The oral dosage form is a route choice for the treatment of NAFLD as it is safe and convenient [18]. The most well-recognized rule is the Lipinski rule [19], which argues that any compound considered as drug should have hydrogen bond acceptors (HBAs) ≤ 10 and hydrogen bond donors (HBDs) ≤ 5, molecular weight (MW) ≤ 500 Da, and partition coefficient (log *p*) ≤ 5 [20]. Another well-known is the Veber rule [21], which states two other criteria: polar surface area (PSA) ≤ 140 Å^2^ and number of rotatable bonds (NRB) ≤ 10.

**Figure 2:**
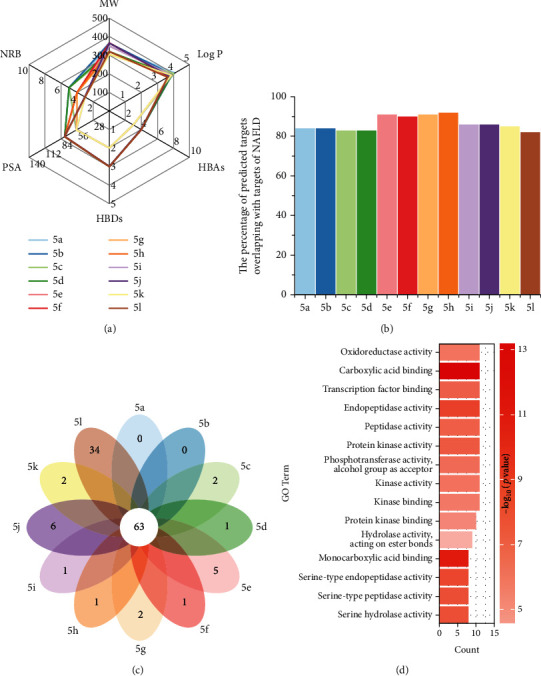
Computational prediction results of twelve compounds **5a-l**. (a) Drug-likeness features of compounds **5a-l**. (b) The percentage of predicted targets of each compound overlapped with targets of NAFLD. (c) The predicted targets on the petal diagram; each petal represents the unique targets of a compound, different colors represent different compounds, and the number in the middle represents 63 common targets of these twelve compounds. (d) Gene Ontology (GO) molecular function term of 63 common predicted targets.

The twelve designed compounds in our lab were checked for the drug-likeness features (Figure 2(a)). MW was an important parameter in therapeutic drug action. Low MW drug compounds (≤500 Da) were easily transported. In designing these twelve molecules, we purposely controlled MW within 500 Da. All compounds exhibited favorable log *p*, which was a vital consideration for suitable lipid solubility for the drugs to penetrate through biomembranes. The presence of HBAs and HBDs in the molecular structure of a solute favors aqueous solvation and tends to make the solute less lipophilic [22]. HBAs and HBDs in the query compounds were found to be less than 5. PSA values of all compounds were found to be less than 84 Å^2^, suggesting good bioavailability by oral route [20]. Molecular flexibility was another critical factor in controlling the bioavailability. NRB had an apparent relationship with molecular flexibility and the permeation rate. All compounds showed proper NRB. From the radar plot, all compounds fully complied with Lipinski and Veber's rules, suggesting that they would display feasible oral use [23].

#### 2.1.2. Target Prediction

A fundamental step in the discovery context is to predict the targets of a given drug-like compound (ligand) and investigate its potential pharmacological effects. To achieve this process, *in silico* ligand-based target prediction is regarded as an effective tool and has shown high-quality performance [24]. The ligand-based method leverages the similar property principle, which states that similar compounds typically share enough structural similarity and have a high probability of binding to similar drug targets [25]. Based on this principle, the ligand-based method is done by comparison of a new ligand to a set of reference ligands of known drug targets. The three most popular ligand-based programs, PharmMapper [26], DIGEP-Pred [27], and TargetNet [28], were applied to predict targets of the twelve designed compounds. Detailed information on predicted targets was presented in Supplementary materials (available here). We analyzed the predicted targets to assess whether these twelve compounds had therapeutic potential against NAFLD. As shown in Figure 2(b), each column/color indicated the overlapped percentage with NAFLD targets of a compound indicated on the *X*-axis. We found that most predicted targets (≥80%) of each compound overlapped with the molecular targets of NAFLD, with some (targets of compounds **5e**, **5f**, **5g**, and **5h**) even reaching 90%.

Figure 2(c) shows the predicted targets on the petal diagram. Each petal indicated the unique targets of a compound, and the core value was the number of common targets of these twelve compounds. A total of 63 common predicted targets were obtained (Table 1). GO provides a representation of terms used to describe genes and their molecular functions. GO analysis was performed on these 63 common targets (Figure 2(d)). Based on the count of targets belonging to each term, the top one GO molecular function term was “oxidoreductase activity.” These results suggest that these twelve designed compounds may have therapeutic utility for the treatment of NAFLD through modulating oxidoreductase activity and could be considered in the next-step synthesis.

### 2.2. Synthesis of Twelve Phenyl Ketone Derivatives

**Figure 3:**
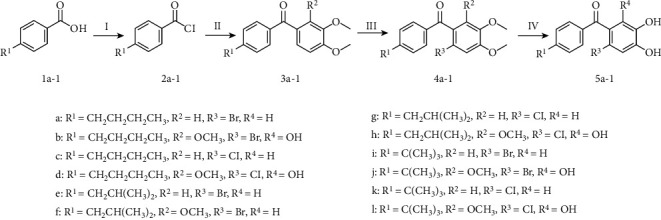
Synthetic routes of target compounds **5a-l**.

Encouraged by the above prediction results, all twelve compounds were selected for the chemical synthesis. Overall synthetic route of phenyl ketone derivatives **5a-l** is outlined in Figure 3. Primarily, intermediates **2a-l** were prepared by treating **1a-l** with commercially available dimethylformamide (DMF) in SOCl_2_. Subsequent Friedel–Crafts' acylation provided the second benzene ring via AlCl_3_-catalyzing. **2a-l** immediately reacted with electron-donating substituted aryl rings such as 1,2-dimethoxy-benzene and 1,2,3-trimethoxy-benzene to afford corresponding **3a-l**. Further, the resulting **3a-l** underwent halogenation with SO_2_Cl_2_ or N-bromosuccinimide (NBS) to give **4a-l**. Finally, **4a-l** were demethylated with BBr_3_, leaving the hydroxyl groups exposed. Formation of desired compounds **5a-l** was achieved.

### 2.3. Experimental Validation of Prediction Results

#### 2.3.1. *In Vitro* Activity Initial Validation

A widely accepted modeling condition was used to resemble the NAFLD condition *in vitro*. HepG2 cells were treated with 500 *μ*M palmitic acid (PA) for 24 hours [29, 30]. The effects of these novel compounds were tested by 3-(4,5-dimethyl-2-thiazolyl)-2,5-diphenyl-2-H-tetrazolium bromide (MTT) method and expressed as 50% of maximal effect (EC_50_) values. As shown in Table 2, the results indicated that this series of compounds exhibited excellent potencies (EC_50_values ≤ 13.5 *μ*M), which initially verified the results of our computational prediction. We found that three butyl-substitution groups on phenyl ketone bearing analogues resulted in different potencies. The isobutyl group substitutions (**5e**, **5f**, **5g**, and **5h**) showed the strongest potency, while the n-butyl substitutions (**5a**, **5b**, **5c**, and **5d**) and tert-butyl (**5i**, **5j**, **5k**, and **5l**) caused lower potency. This trend was in agreement with the percentage of overlapping targets listed in Figure 2(b).

**Figure 4:**
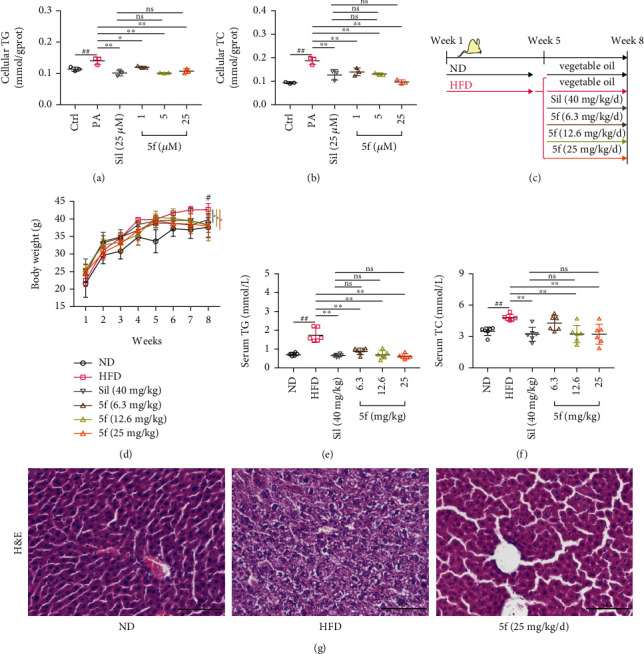
Effects of compound **5f** in HepG2 cells and mice. (a) Cellular triglyceride (TG) accumulation. (b) The total cellular cholesterol (TC) level. (c) A schematic illustration of the animal experiment. (d) The weight growth of mice. (e) Serum TG accumulation. (f) Serum TC accumulation. (g) Representative images of hematoxylin and eosin- (H&E-) stained sectioned livers from the mice. Data are mean ± SD; compared with that in the control group, ^#^*p* < 0.05 and ^##^*p* < 0.01; compared with that in the model group, ^∗^*p* < 0.05 and ^∗∗^*p* < 0.01. ns: not significant; Ctrl: control; PA: palmitic acid; Sil: silybin; ND: normal diet; HFD: high-fat diet.

The most promising compound, **5f** (EC_50_ = 10.2 nM), was further evaluated for the activities to reduce triglycerides (TG) and total cholesterol (TC) levels. As presented in Figures 4(a) and 4(b), PA treatment robustly increased TG and TC levels in cells (*p* < 0.01). Treatment of HepG2 with various dose levels of **5f** decreased the TG accumulation and TC content compared with the model group (*p* < 0.05 or *p* < 0.01). Silybin (sil), a polyphenolic flavonoid, had been used for a long time to treat acute and chronic liver diseases [31]. It was used as the reference group. Compared to 25 *μ*M sil treatment, 10 *μ*M and 25 *μ*M **5f** treatment showed no statistical differences, implying that **5f** may have a lower effective dose.

#### 2.3.2. *In Vivo* Activity Validation

The effect of compound **5f** on NAFLD was further investigated with a murine model of a high-fat diet (HFD) induced [32]. Mice fed normal diet (ND) served as control (Figure 4(c)). It is known that substantial weight loss will benefit NAFLD [33]. As shown in Figure 4(d), the body weight of the HFD-fed group was significantly increased compared with the animals kept on a standard chow (*p* < 0.05). Treatment with **5f** (12.6 mg/kg or 25 mg/kg) resulted in significant weight loss (*p* < 0.05). Consistent with these observations, serum TC and TG contents (Figures 4(e) and 4(f)) remarkably reduced after 12.6 mg/kg and 25 mg/kg **5f** treatment (*p* < 0.01) compared with HFD-fed group, confirming the effects of **5f***in vivo*. Regarding body weight, serum TC and TG levels, 12.6 mg/kg and 25 mg/kg **5f** treatment showed no statistical differences compared to 40 mg/kg sil treatment.

As the “gold standard” for assessing hepatic damage is liver biopsy, liver sections were used for hematoxylin and eosin (H&E) staining. As shown in Figure 4(g), an accelerated progression of fatty liver was seen in mice receiving HFD, which exhibited hepatocyte ballooning and microvesicular steatosis in comparison with the mice maintained on ND. Consistent with the dynamics of serum TC and TG accumulation, histological examination of liver sections confirmed significant improvements in the **5f**-treated mice in comparison to the HFD-fed mice. Results of animal experiments were consistent with *in vitro* experiments that supported the hypothesis that **5f** had promising therapeutic potential against NAFLD.

#### 2.3.3. *In Vitro* Activity Further Validation by Quantitative Proteomics

**Figure 5:**
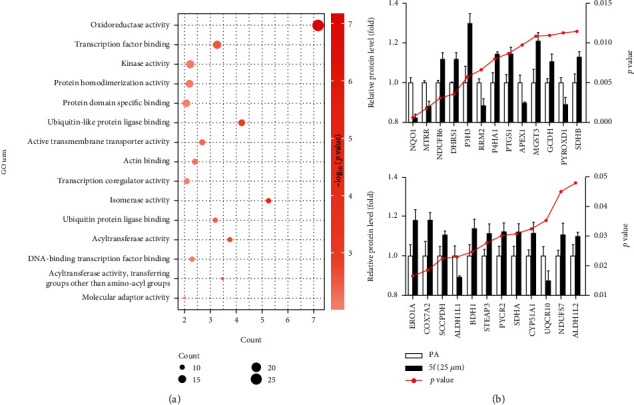
Proteomics results of compound **5f**-treated HepG2 cells. (a) The top fifteen GO molecular function terms. (b) The relative protein level of twenty-five differentially expressed proteins (DEPs) which were clustered in the term “oxidoreductase activity.” DEPs are presented as official gene names and are arranged from left to right in order of increasing *p* value.

To further test this hypothesis, we used tandem mass tag-based quantitative proteomics to identify differentially expressed proteins (DEPs) between the PA-treated group and 25 *μ*M compound **5f** intervention group through *in vitro* experiments. Totally, 290 DEPs were identified (*p* value < 0.05 and fold change > 1.1). GO analysis was then carried out, and the top fifteen molecular function terms are listed in Figure 5(a). Of particular interest, the most significantly enriched term was “oxidoreductase activity.” This highlights the central role of this term in **5f** intervention, suggesting the activity of **5f** might be mediated by oxidoreductase activity. The term “oxidoreductase activity” was also the top term of enrichment analysis results in Figure 2(d), which further supported the credibility of our computational prediction results. Oxidoreductase activity was shown to be directly connected to the modulation of cellular redox balance to rapidly respond to oxidative stress [34]. Oxidative stress is undoubtedly a major player in human NAFLD [35]. One of the mechanisms involved in the condition of both NAFLD and its aggressive form NASH is the oxidative stress coming from oxidase (e.g., NADPH oxidase and xanthine oxidase) [36]. These enzymes could therefore be potential therapeutic targets.

As can be seen in Figure 5(b), twenty-five DEPs were clustered in this term and arranged from left to right in order of increasing *p* value. The two most significant DEPs were NQO1 and MTRR, which were two flavoreductases. NQO1 (NADPH quinone oxidoreductase 1) can catalyze quinone through a one-step two-electron reduction process, scavenging superoxide and protecting endogenous antioxidants. It is well known that NQO1 and MTRR are related [37]. The MTRR system is a key endogenous antioxidative enzyme system which defends the tissues and proteins from oxidative damage [38]. Further, there are three other DEPs (NDUFB6, CYP51A1, and NDUFS7) which are also linked with NQO1. CYP51A1 belongs to a large superfamily of heme-containing monooxygenases involved in the oxidative metabolism of an enormous diversity of substrates. This enzyme requires electrons for its activity, and the electrons are supplied by NQO1 [39]. The hepatocyte-specific CYP51A1 partial KO mice display severe liver inflammation and fibrosis [40]. Six DEGs (NDUFB6, SDHB, COX7A2, SDHA, UQCR10, and NDUFS7) were related to four respiratory chain complexes of mitochondria, which played essential roles in cellular energy production by the process of oxidative phosphorylation [41]. NDUFB6 and NDUFS7 are two subunits of the mitochondrial membrane respiratory chain NADH dehydrogenase (complex I), which is a redox-driven proton pump [42].

**Figure 6:**
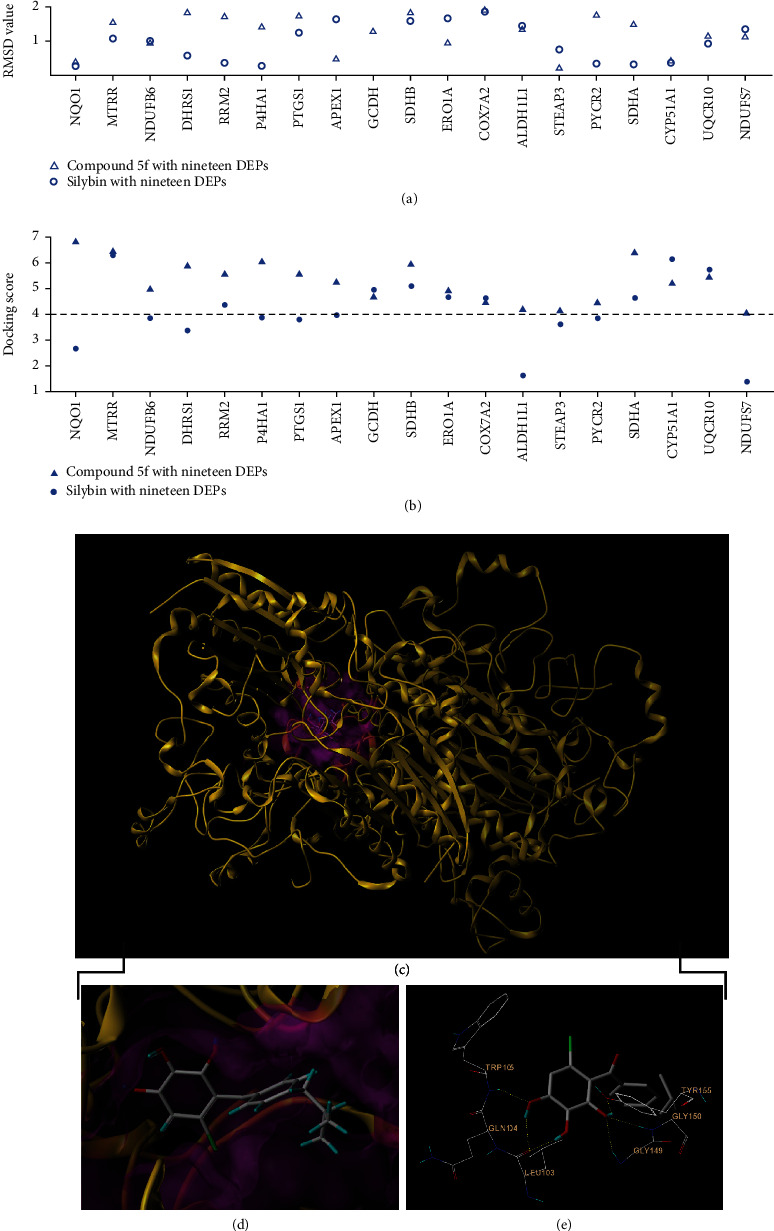
The results of molecular docking. (a) The root-mean-square deviation (RMSD) value of compound **5f** or sil with nineteen DEPs clustered in the term “oxidoreductase activity.” (b) The docking score of compound **5f** or sil with nineteen DEPs clustered in the term “oxidoreductase activity.” (c–e) The visualizations of **5f** with NQO1. **5f** is depicted in capped sticks representation, while NQO1 is shown as gold ribbon. The ligand surface is indicated in magenta. The interacting residues are shown in line representation. H-bond interactions are in yellow dotted lines.

Computational methods like molecular docking provide a necessarily complementary way to experimental methods to predict molecular interactions and explore binding modes of small molecules with targets [43]. Compound **5f** was docked into the nineteen DEPs clustered in the term “oxidoreductase activity,” and the 3D structures for the other six DEPs were not available. Like the above-mentioned experiments on cells and mice, sil was used as the reference compound to perform docking. A docking is considered successful if the root-mean-square deviation (RMSD) is less than or equal to 2.0 [44]. The detailed RMSD results for all docking runs are shown in Figure 6(a). Hollow triangles indicated RMSD values for docking of compound **5f** into nineteen DEPs. Hollow circles indicated RMSD values for docking of sil into nineteen DEPs. All dockings were within an RMSD threshold of 2.0, and this ensured the docking accuracy. According to the rule of SYBYL software, the total score shows the binding affinity between the drug and target. A higher total score suggests a closer interaction between drug and potential target. When total score is ≥4, the drug is considered a candidate to be studied continuously [45]. The obtained total scores of compound **5f** with nineteen DEPs were indicated with filled triangles in Figure 6(b), and all of them were larger than 4. Interestingly, we found that the orders of docking scores in Figure 6(b) mostly coincided with the trends of *p* values in Figure 5(b). The obtained total scores of sil with nineteen DEPs were indicated with filled circles in Figure 6(b). It can be found that compound **5f** scored higher than sil against most DEPs. The binding of NQO1 with **5f** yielded the highest total score value, and the visualization is shown in Figures 6(c) and 6(d). Figure 6(e) shows that compound **5f** was connected to NQO1 through six hydrogen bonds, suggesting that **5f** was very likely an excellent substrate for NQO1. Similar to previous studies [46–48], **5f** could also bind firmly to the TRP105 residue by hydrogen bonding interaction. To sum up, our proteomics analysis indicated that compound **5f** could exert promising therapeutic potential against NAFLD via modulating oxidoreductase activity.

## 3. Conclusion

Such prediction of drug-likeness and biological activities before synthesis can decrease the attrition of drug candidates. This highly accessible computational prediction is worthy of popularization and application. Of these novel phenyl ketone derivatives, we identified **5f** with dual lipid/weight-lowering properties through modulating oxidoreductase activity as a potential therapy for NAFLD, suggesting that **5f** may be worthy of further study.

## 4. Materials and Methods

### 4.1. Computational Method Prediction

Drug-likeness values of designed compounds were collected from the SwissADME open-access web tool [49]. Three platforms were used to predict targets of designed compounds with limitation to “Homo sapiens”: PharmMapper (normalized fit score ≥ 0.5) [26], DIGEP-Pred (Pa value ≥ 0.5) [27], and TargetNet (Prob value ≥ 0.5) [28]. The duplicate targets were removed. To obtain disease targets, NAFLD-related words, such as “Nonalcoholic Fatty Liver Disease,” “NAFLD,” “NAFL,” “Nonalcoholic Steatohepatitis,” “NASH,” “Simple Fatty Liver,” “SFL,” and “Steatohepatitis” were used as keywords to query GeneCards database (relevance score ≥ 1) [50]. After the removal of duplicate targets, 4996 targets were identified as potential targets of NAFLD, which were provided in Supplementary Material. The information on these targets was standardized using the UniProt database [51]. GO enrichment analysis was performed by Metascape [52].

### 4.2. Chemistry

X-4 digital melting point apparatus (Taike, China) was used to record melting points. Bruker Avance (USA) was used to record ^1^H and ^13^C NMR spectra. Waters ZQ2000 (USA) was used to determine the MS spectra. FTIR spectra were recorded on a Bruker Tensor II spectrometer (USA). All chemicals were analytically pure and were used without any further purification. The following chemicals were obtained from Energy Chemical (China): 4-butyl-benzoyl chloride, 4-tertbutyl-benzoyl chloride, 1,2,3-trimethoxy-benzene, NBS, and BBr_3_. The following chemicals were purchased from Fengchuan Chemical (China): DMF, AlCl_3_, and dichloromethane (DCM). 4-Isobutyl-benzoyl chloride was from TCI Chemicals (China). SOCl_2_ was from Damao Chemical (China). 1,2-Dimethoxy-benzene was from Aladdin (China).

#### 4.2.1. Synthesis of 4-Butyl-benzoyl Chloride (**2a-l**)

Primarily, 4-butyl-benzoic acid was added to SOCl_2_. After dissolution, DMF was added, and the solution was stirred at 80°C for 8 h. After the reaction was stopped, excess SOCl_2_ was removed by reduced pressure distillation. The resulting solution was washed with DCM.

#### 4.2.2. Synthesis of (4-Butyl-phenyl)-(methoxy-phenyl)-methanone (**3a-l**)

1,2-Dimethoxy-benzene or 1,2,3-trimethoxy-benzene was added to DCM at 0°C for 10 min. The above-obtained **2a-l** was added, followed by AlCl_3_. After completing the addition, the mixture was brought back to room temperature and stirred for 3.5 hours. The reaction was stopped by adding ice water slowly. The solution was extracted with an equal volume of DCM three times, dried over with Na_2_SO_4_, and then dried under vacuum.

#### 4.2.3. Synthesis of (4-Butyl-phenyl)-(halo-methoxy-phenyl)-methanone (**4a-l**)

The above obtained **3a-l** was dissolved in DCM. For chloro-substitution, a solution of SO_2_Cl_2_ and DCM was added at room temperature. For bromo-substitution, NBS was then added. The reaction was performed for 3 h. The solution was extracted with DCM three times, dried over Na_2_SO_4_, and then dried under vacuum.

#### 4.2.4. Synthesis of (4-Butyl-phenyl)-(halo-hydroxy-phenyl)-methanone (**5a-l**)

The above obtained **4a-l** was dissolved in DCM and stirred for 10 min. After that, BBr_3_ was slowly added. The reaction was performed at room temperature for 2 h. The reaction was stopped by adding ice water. The solution was extracted with DCM three times, dried over Na_2_SO_4_, and then dried under vacuum.

All twelve compounds were synthesized and confirmed by ^1^H and ^13^C NMR, MS, and IR, which were provided in Supplementary Material. The analyses of these spectral data were as follows.

#### 4.2.5. (2-Bromo-4,5-dihydroxy-phenyl)-(4-butyl-phenyl)-methanone (**5a**)

Green solid. Yield: 45.5%. M.P.: 88.0-89.2°C. ^1^H NMR*δ*/ppm (400 MHz, d_6_-DMSO): 7.72 (d, J = 8.2 Hz, 2H, Ar H), 7.25 (d, J = 8.5 Hz, 2H, Ar H), 7.08 (s, 1H, Ar H), 7.00 (s, 1H, Ar H), 6.83 (s, 1H, OH), 6.80 (s, 1H, OH), 2.70–2.64 (m, 2H, CH_2_), 1.62 (q, J = 7.6 Hz, 2H, CH_2_), 1.36 (h, J = 7.4 Hz, 2H, CH_2_), 0.93 (t, J = 7.4 Hz, 3H, CH_3_). ^13^C NMR*δ*/ppm (101 MHz, d_6_-DMSO): 197.91 (CO), 150.45 (Ar C), 147.01 (Ar C), 143.17 (Ar C), 133.67 (Ar C), 131.76 (Ar C), 131.01 (Ar C), 128.79 (Ar C), 119.99 (Ar C), 116.36 (Ar C), 110.13 (Ar C), 35.88 (CH_2_), 33.11 (CH_2_), 22.36 (CH_2_), 13.89 (CH_3_). MS (M^−^): 349.22. IR cm^−1^: 3241 br m, 2360 m, 1649 s, 1601 s, 1278 s, 1175 s, 598 s.

#### 4.2.6. (6-Bromo-2,3,4-trihydroxy-phenyl)-(4-butyl-phenyl)-methanone (**5b**)

Brown solid. Yield: 47.5%. M.P.: 58.0-59.2°C. ^1^H NMR*δ*/ppm (400 MHz, d_6_-DMSO): 12.56 (s, 1H, OH), 7.59 (d, *J* = 8.2 Hz, 2H, Ar H), 7.43 (s, 1H, Ar H), 7.33 (d, *J* = 8.2 Hz, 2H, Ar H), 6.44 (s, 1H, OH), 6.07 (s, 1H, OH), 2.74–2.68 (m, 2H, CH_2_), 1.66 (p, *J* = 7.6 Hz, 2H, CH_2_), 1.40 (h, *J* = 7.4 Hz, 2H, CH_2_), 0.96 (t, *J* = 7.4 Hz, 3H, CH_3_). ^13^C NMR*δ*/ppm (101 MHz, d_6_-DMSO): 199.71 (CO), 150.68 (Ar C), 148.02 (Ar C), 146.66 (Ar C), 134.76 (Ar C), 132.38 (Ar C), 129.30 (Ar C), 128.60 (Ar C), 128.03 (Ar C), 114.12 (Ar C), 99.39 (Ar C), 35.71 (CH_2_), 33.29 (CH_2_), 22.38 (CH_2_), 13.93 (CH_3_). MS (M^−^): 365.15. IR cm^−1^: 3206 br m, 1590 s, 1429 s, 1289 s, 1180 s, 887 s.

#### 4.2.7. (4-Butyl-phenyl)-(2-chloro-4,5-dihydroxy-phenyl)-methanone (**5c**)

Brown solid. Yield: 47.2%. M.P.: 54.8-55.2°C. ^1^H NMR*δ*/ppm (400 MHz, d_6_-DMSO): 7.62 (d, *J* = 8.1 Hz, 2H, Ar H), 7.34 (d, *J* = 7.9 Hz, 2H, Ar H), 6.87 (d, 2H, Ar H), 2.66 (d, J = 7.7 Hz, 2H, CH_2_), 1.56 (*p*, *J* = 7.5 Hz, 2H, CH_2_), 1.30 (h, *J* = 7.4 Hz, 2H, CH_2_), 0.89 (t, *J* = 7.3 Hz, 3H, CH_3_). ^13^C NMR*δ*/ppm (101 MHz, d_6_-DMSO): 193.65 (CO), 148.59 (Ar C), 148.32 (Ar C), 144.27 (Ar C), 134.53 (Ar C), 129.72 (Ar C), 128.62 (Ar C), 128.37 (Ar C), 120.22 (Ar C), 116.54 (Ar C), 116.39 (Ar C), 34.79 (CH_2_), 32.67 (CH_2_), 21.72 (CH_2_), 13.69 (CH_3_). MS (M^−^): 303.34. IR cm^−1^: 3122 br w, 1647 m, 1607 m, 1276 s, 608 m.

#### 4.2.8. (4-Butyl-phenyl)-(6-chloro-2,3,4-trihydroxy-phenyl)-methanone (**5d**)

Yellow solid. Yield: 42.1%. M.P.: 115.2-115.9°C. ^1^H NMR*δ*/ppm (400 MHz, d_6_-DMSO): 12.56 (s, 1H, OH), 7.58 (d, *J* = 7.9 Hz, 2H, Ar H), 7.32 (s, 2H, Ar H), 7.26 (s, 1H, Ar H), 2.69 (t, *J* = 7.7 Hz, 2H, CH_2_), 1.72–1.57 (m, 2H, CH_2_), 1.38 (q, *J* = 14.7, 7.3 Hz, 2H, CH_2_), 0.95 (t, *J* = 7.3 Hz, 3H, CH_3_). ^13^C NMR*δ*/ppm (101 MHz, d_6_-DMSO): 199.81 (CO), 150.29 (Ar C), 147.98 (Ar C), 145.88 (Ar C), 134.78 (Ar C), 132.63 (Ar C), 129.27 (Ar C), 128.58 (Ar C), 125.03 (Ar C), 113.14 (Ar C), 111.43 (Ar C), 35.70 (CH_2_), 33.28 (CH_2_), 22.37 (CH_2_), 13.92 (CH_3_). MS (M^−^): 319.35. IR cm^−1^: 3178 br m, 1620 s, 1288 s, 1181 s, 647 s.

#### 4.2.9. (2-Bromo-4,5-dihydroxy-phenyl)-(4-isobutyl-phenyl)-methanone (**5e**)

Brown solid. Yield: 46.7%. M.P.: 104.2-105.6°C. ^1^H NMR*δ*/ppm (400 MHz, d_6_-DMSO): 9.78 (s, 1H, OH), 7.63 (d, *J* = 8.2 Hz, 2H, Ar H), 7.32 (d, *J* = 8.0 Hz, 2H, Ar H), 6.87 (s, 1H, Ar H), 6.81 (s, 1H, Ar H), 2.53 (d, *J* = 7.2 Hz, 2H, CH_2_), 1.88 (h, *J* = 6.8 Hz, 1H, CH), 0.87 (d, *J* = 6.5 Hz, 6H, 2 × CH_3_). ^13^C NMR*δ*/ppm (101 MHz, d_6_-DMSO): 193.68 (CO), 148.35 (Ar C), 147.39 (Ar C), 144.26 (Ar C), 134.61 (Ar C), 129.57 (Ar C), 129.27 (Ar C), 128.35 (Ar C), 120.26 (Ar C), 116.49 (d, *J* = 13.7 Hz, Ar C)), 44.43 (CH_2_), 38.94 (CH), 22.08 (CH_3_). MS (M^−^): 347.18. IR cm^−1^: 3239 br m, 1649 s, 1601 s, 1280 s, 1176 s, 756 s.

#### 4.2.10. (6-Bromo-2,3,4-trihydroxy-phenyl)-(4-isobutyl-phenyl)-methanone (**5f**)

Brown solid. Yield: 45.7%. M.P.: 137.0-138.0°C. ^1^H NMR*δ*/ppm (400 MHz, d_6_-DMSO): 12.55 (s, 1H, OH), 7.59 (d, *J* = 7.8 Hz, 2H, Ar H), 7.43 (s, 1H, Ar H), 7.29 (d, *J* = 7.8 Hz, 2H, Ar H), 6.27 (s, 1H, OH), 5.85 (s, 1H, OH), 2.57 (d, *J* = 7.2 Hz, 2H, CH_2_), 1.94 (q, *J* = 13.5, 6.7 Hz, 1H, CH), 0.95 (s, 3H, CH_3_), 0.94 (s, 3H, CH_3_). ^13^C NMR*δ*/ppm (101 MHz, d_6_-DMSO): 199.68 (CO), 150.65 (Ar C), 146.85 (Ar C), 146.48 (Ar C), 134.81 (Ar C), 132.35 (Ar C), 129.28 (Ar C), 129.17 (Ar C), 127.98 (Ar C), 114.14 (Ar C), 99.38 (Ar C), 45.41 (CH_2_), 30.15 (CH), 22.40 (CH_3_). MS (M^−^): 365.26. IR cm^−1^: 3341 br m, 1607 m, 1287 s, 1178 s, 882 s, 781 s.

#### 4.2.11. (2-Chloro-4,5-dihydroxy-phenyl)-(4-isobutyl-phenyl)-methanone (**5g**)

Brown solid. Yield: 50.3%. M.P.: 105.0-105.7°C. ^1^H NMR*δ*/ppm (400 MHz, d_6_-DMSO): 7.63 (d, *J* = 1.7 Hz, 1H, Ar H), 7.61 (s, 1H, Ar H), 7.32 (s, 1H, Ar H), 7.30 (s, 1H, Ar H), 6.87 (s, 1H, Ar H), 6.80 (s, 1H, Ar H), 2.53 (s, 2H, CH_2_), 1.87 (h, *J* = 6.9 Hz, 1H, CH), 0.86 (d, *J* = 6.6 Hz, 6H, 2 × CH_3_). ^13^C NMR*δ*/ppm (101 MHz, d_6_-DMSO): 193.68 (CO), 148.35 (Ar C), 147.39 (Ar C), 144.26 (Ar C), 134.61 (Ar C), 129.57 (Ar C), 129.27 (Ar C), 128.35 (Ar C), 120.26 (Ar C), 116.56 (Ar C), 116.42 (Ar C), 44.43 (CH_2_), 29.47 (CH), 22.09 (CH_3_). MS (M^−^): 303.33. IR cm^−1^: 3242 br m, 1648 s, 1279 s, 758 s.

#### 4.2.12. (6-Chloro-2,3,4-trihydroxy-phenyl)-(4-isobutyl-phenyl)-methanone (**5h**)

Yellow solid. Yield: 42.8%. M.P.: 138.5-139.2°C. ^1^H NMR*δ*/ppm (400 MHz, d_6_-DMSO): 12.82 (s, 1H, OH), 7.84 (d, *J* = 1.7 Hz, 1H, Ar H), 7.83 (d, *J* = 2.0 Hz, 1H, Ar H), 7.54 (s, 1H, Ar H), 7.52 (s, 2H, Ar H), 2.81 (d, *J* = 7.1 Hz, 2H, CH_2_), 2.18 (h, *J* = 6.7 Hz, 1H, CH), 1.19 (d, *J* = 6.7 Hz, 6H, 2 × CH_3_). ^13^C NMR*δ*/ppm (101 MHz, d_6_-DMSO): 199.83 (CO), 150.24 (Ar C), 146.82 (Ar C), 145.82 (Ar C), 134.79 (Ar C), 132.59 (Ar C), 129.25 (Ar C), 129.16 (Ar C), 125.05 (Ar C), 113.14 (Ar C), 111.45 (Ar C), 45.39 (CH_2_), 30.13 (CH), 22.38 (CH_3_). MS (M^−^): 319.35. IR cm^−1^: 3333 br m, 1606 m, 1288 s, 1182 s, 900 m, 760 m.

#### 4.2.13. (2-Bromo-4,5-dihydroxy-phenyl)-(4-tert-butyl-phenyl)-methanone (**5i**)

Brown solid. Yield: 42.5%. M.P.: 113.0-114.5°C. ^1^H NMR*δ*/ppm (400 MHz, d_6_-DMSO): 9.95 (s, 1H, OH), 7.65 (d, *J* = 8.6 Hz, 2H, Ar H), 7.56 (d, *J* = 8.6 Hz, 2H, Ar H), 6.87 (s, 1H, Ar H), 6.79 (s, 1H, Ar H), 1.31 (s, 9H, 3 × CH_3_). ^13^C NMR*δ*/ppm (101 MHz, d_6_-DMSO): 193.58 (CO), 156.56 (Ar C), 148.32 (Ar C), 144.24 (Ar C), 134.26 (Ar C), 129.58 (Ar C), 128.33 (Ar C), 125.56 (Ar C), 120.24 (Ar C), 116.57 (Ar C), 116.40 (Ar C), 34.88 (C(CH_3_)_3_), 30.76 (CH_3_). MS (M^−^): 349.13. IR cm^−1^: 3219 br m, 2920 s, 1649 s, 1599 s, 1280 s, 878 s.

#### 4.2.14. (6-Bromo-2,3,4-trihydroxy-phenyl)-(4-tert-butyl-phenyl)-methanone (**5j**)

Brown solid. Yield: 40.0% M.P.: 131.3-132.5°C. ^1^H NMR*δ*/ppm (400 MHz, d_6_-DMSO): 12.58 (s, 1H, OH), 7.62 (d, *J* = 8.3 Hz, 2H, Ar H), 7.54 (d, *J* = 8.2 Hz, 2H, Ar H), 7.45 (s, 1H, Ar H), 1.38 (s, 9H, 3 × CH_3_). ^13^C NMR*δ*/ppm (101 MHz, d_6_-DMSO): 199.65 (CO), 156.08 (Ar C), 150.66 (Ar C), 146.49 (Ar C), 134.52 (Ar C), 132.35 (Ar C), 129.14 (Ar C), 128.00 (Ar C), 125.57 (Ar C), 114.15 (Ar C), 99.38 (Ar C), 35.16 (C(CH_3_)_3_), 31.16 (CH_3_). MS (M^−^): 365.31. IR cm^−1^: 3476 br m, 1603 s, 1265 s, 885 s.

#### 4.2.15. (4-Tert-butyl-phenyl)-(2-chloro-4,5-dihydroxy-phenyl)-methanone (**5k**)

Brown solid. Yield: 45.8%. M.P.: 116.3-117.1°C. ^1^H NMR*δ*/ppm (400 MHz, d_6_-DMSO): 7.65 (s, 1H Ar H), 7.63 (s, 1H Ar H), 7.56 (s, 1H Ar H), 7.54 (s, 1H Ar H), 6.87 (s, 1H Ar H), 6.79 (s, 1H Ar H), 1.30 (s, 9H, 3 × CH_3_). ^13^C NMR*δ*/ppm (101 MHz, d_6_-DMSO): 193.58 (CO), 156.56 (Ar C), 148.32 (Ar C), 144.24 (Ar C), 134.26 (Ar C), 129.58 (Ar C), 128.33 (Ar C), 125.56 (Ar C), 120.24 (Ar C), 116.57 (Ar C), 116.40 (Ar C), 34.88 (C(CH_3_)_3_), 30.76 (CH_3_). MS (M^−^): 303.33. IR cm^−1^: 3234 br m, 1648 s, 1282 s, 725 s.

#### 4.2.16. (4-Tert-butyl-phenyl)-(6-chloro-2,3,4-trihydroxy-phenyl)-methanone (**5l**)

Yellow solid. Yield: 39.2%. M.P.: 72.8-73.6°C. ^1^H NMR*δ*/ppm (400 MHz, d_6_-DMSO): 12.61 (s, 1H, OH), 7.59–7.52 (d, 3H, Ar H), 6.57 (s, 1H, Ar H), 6.24 (s, 1H, Ar H), 1.40 (s, 9H, 3 × CH_3_). ^13^C NMR*δ*/ppm (101 MHz, d_6_-DMSO): 199.82 (CO), 156.04 (Ar C), 150.29 (Ar C), 145.90 (Ar C), 134.52 (Ar C), 132.61 (Ar C), 129.11 (Ar C), 125.53 (Ar C), 125.08 (Ar C), 113.14 (Ar C), 111.45 (Ar C), 35.15 (C(CH_3_)_3_), 31.14 (CH_3_). MS (M^−^): 319.34. IR cm^−1^: 3272 br m, 1605 s, 1283 s, 902 s, 780 s.

### 4.3. Biological Assays

#### 4.3.1. Cell Culture and Treatment

HepG2 cells purchased from Procell (China) were grown in DMEM (Boster, China) supplemented with 10% fetal bovine serum (Minhai, China) and 1% penicillin/streptomycin at 37°C and 5% CO_2_. After growing HepG2 cells overnight, the cells were treated with 500 *μ*M PA (Solarbio, China) for 24 h. For pharmacological treatments, HepG2 cells were treated with different concentrations of compound 5f simultaneously. The treated cells were subjected to a cell viability and TC/TG content assay. Cell viability was measured by MTT assays. 10 *μ*l MTT (5 mg/ml in PBS, Solarbio, China) solution was added and incubated for 4 hours. Media were removed and replaced with 100 *μ*l of DMSO. The absorbance was measured at 490 nm using a microplate reader (SpectraMax Plus 384, Molecular Devices, USA). EC_50_ values were calculated. To determine TG/TC contents, the cells were gently washed with phosphate-buffered saline and disrupted by sonication. The contents were normalized to total protein using a BCA protein assay kit (Boster, China) and quantified based on the instructions on the corresponding assay kit (Jiancheng, China).

#### 4.3.2. Animal Studies

36 male ICR mice (provided by the Experimental Animal Center of Shanxi Medical University, No. 22829) were fed adaptively for one week and then were randomly divided into six groups. Animals were given either a normal chow diet (control group, *n* = 6) or a high-fat diet (HFD, 60% calories from fat, Jiangsu Synergy Medical Bioengineering Co., Ltd., *n* = 30) for four weeks. After successfully modeling, positive control silybin (40 mg/kg) or compound **5f** (low dose, 6.3 mg/kg; medium dose, 12.6 mg/kg; and high dose, 25 mg/kg) solved in vegetable oil was administrated intragastrically to the assigned HFD groups once every day for three weeks. The dosage for gavage is calculated via the conversion method of human and mice in “Pharmacological Experimental Methodology” edited by Professor Xu Shuyun. Meanwhile, the control and model groups were administered the same amount of vegetable oil (vehicle). Mice were anesthetized with isoflurane inhalation anesthesia. The blood was sampled from the orbital venous. The levels of TG and TC in serum were investigated according to the manufacturer's protocols. Liver samples were fixed in buffered formalin (10%) overnight and embedded in paraffin. 3~5 *μ*m thick serial sections were made from paraffin-embedded tissue and then stained with hematoxylin and eosin (H&E) according to standard protocols.

#### 4.3.3. Proteomics

The model and **5f** groups of HepG2 cells were treated with 500 *μ*M PA for 24 h. **5f** groups additionally received 25 *μ*M **5f** simultaneously. After being harvested, the cells were washed with cold PBS and lysed with SDT buffer containing 4% (*w*/*v*) SDS, 100 mM Tris/HCl, and 1 mM DTT. The samples were quantitated by BCA Assay Kit (Bio-Rad, USA). Subsequently, the proteins were digested by adding trypsin according to the filter-aided sample preparation described by Matthias Mann. The peptides were then desalted on C18 Cartridges (Empore™ SPE Cartridges C18, 3 ml, 7 mm i.d., Sigma, USA) and resolubilized in 40 *μ*l of 0.1% formic acid. The resulting peptide mixtures were labeled using TMT reagents according to the instructions provided by the company (Thermo Fisher, USA). The LC-MS/MS analysis was conducted on an Easy-nLC Liquid Chromatographer (Thermo Fisher, USA) coupled online to a Q Exactive mass spectrometer (Thermo Fisher, USA) at Shanghai Applied Protein Technology Co., Ltd. LC was performed with a constant flow of 300 nL/min using a C18-reversed phase column (Thermo Scientific Acclaim PepMap100, 2 cm^∗^100 *μ*m) connected to a reversed-phase analytical column (Thermo Scientific Easy Column, 10 cm^∗^75 *μ*m^∗^3 *μ*m). Mobile phase A was water with 0.1% formic acid. Mobile phase B was 84% acetonitrile and 0.1% formic acid. The mass spectrometer was run in positive ion mode and a data-dependent mode. Full scan (m/z 300–1800) was obtained at a resolution of 70,000 at m/z 200, followed by high energy collisional dissociation at a resolution of 17,500 at m/z 200. The automatic gain control values were set to 3e6. Dynamic exclusion duration was 40.0 s. The raw HPLC-MS/MS data were searched using MASCOT 2.2 (Matrix Science, UK) embedded into Proteome Discoverer 1.4. Maximum missed cleavages were set to 2 and enzyme specificity to trypsin. The FDR values for peptides were 0.01.

### 4.4. Molecular Docking

The crystal structure of the proteins was downloaded from Protein Data Bank database (https://www.pdbus.org/). The PDB IDs for nineteen DEPs are listed in Table 3. The RMSD was calculated via PyMOL 2.3. SYBYL X-2.0 (Tripos, St. Louis, USA) was used for molecular docking. The Surflex-Dock program was used for the docking calculations. The protomol threshold was set to 0.50 Å [53]. Ligands were rendered flexible in surflex docking routine. The AMBER FF99 force field was utilized for the purpose of energy minimization which was followed by protomol generation.

### 4.5. Statistical Analysis

The data were presented as the mean ± standard error (mean ± SD). The difference between groups was analyzed with one-way ANOVA. *p* < 0.05 were considered statistically significant. Statistical analyses were performed using Prism software (version 7, GraphPad Software).

## Figures and Tables

**Table 1:** The 63 common predicted targets of twelve compounds **5a-l**.

Common predicted targets^a^
ADH5, ADIPOQ, AKR1B1, AKR1C1, AKR1C3, ANXA5, BACE1, CA2, CA7, CASP3, CES2, CFB, CHEK1, CTSK, DHODH, DPP4, EGFR, F10, F2, FABP3, FGFR1, GC, GSK3B, GSTP1, HDAC8, HSD11B1, HSD17B1, HSP90AA1, KDR, KIF11, MAOB, MAPK10, MAPK14, MAPK8, MIF, MMP3, MMP7, MMP8, NOS3, NR1H4, NR3C2, PDE3B, PDE4B, PDE4D, PIM1, PLA2G2A, PNMT, PPARD, PPARG, PTPN1, PTPN11, REN, RORA, SEC14L2, SORD, SRC, STS, TGFBR1, THRA, THRB, TNNC1, TTR, TYMS

^a^Presented as official gene names.

**Table 2:** The 50% of maximal effect values.

Compound	EC_50_ (*μ*M)^a^	Compound	EC_50_ (*μ*M)^a^
5a	13.5 ± 2.72	5g	1.36 × 10^−1^ ± 1.16 × 10^−3^
5b	9.63 ± 1.59	5h	2.37 × 10^−2^ ± 1.16 × 10^−3^
5c	12.3 ± 5.65	5i	10.4 ± 1.29
5d	2.61 ± 1.09	5j	10.0 ± 2.96
5e	5.47 × 10^−1^ ± 1.49 × 10^−3^	5k	11.2 ± 2.07
5f	1.02 × 10^−2^ ± 1.56 × 10^−3^	5l	11.0 ± 2.02

^a^Data are mean ± SD, *n* = 3.

**Table 3:** The PDB IDs for nineteen DEPs.

Gene symbol	PDB ID	Gene symbol	PDB ID
NQO1	1D4A	ERO1A	3AHQ
MTRR	2QTZ	COX7A2	5Z62
NDUFB6	5XTC	ALDH1L1	2BW0
DHRS1	2QQ5	STEAP3	2VNS
RRM2	3OLJ	PYCR2	6LHM
P4HA1	2YQ8	SDHA	6VAX
PTGS1	6Y3C	CYP51A1	3JUS
APEX1	4QHE	UQCR10	5XTE
GCDH	2R0N	NDUFS7	5XTB
SDHB	7KCL	—	—

## Data Availability

The data used to support the findings of this study are included within the article.

## Supplementary Materials

Supplementary 1 Supplementary Figure 1S–48S: the ^1^H and ^13^C NMR, MS, and IR spectra of twelve phenyl ketone compounds.

Supplementary 2 Supplementary Material-predicted targets: the detailed information on predicted targets of twelve phenyl ketone compounds.
