# Supplementary material for: Highly Accessible Computational Prediction and In Vivo/In Vitro Experimental Validation: Novel Synthetic Phenyl Ketone Derivatives as Promising Agents against NAFLD via Modulating Oxidoreductase Activity
Source: Oxid Med Cell Longev. 2023 Jan 9;2023:3782230. doi: 10.1155/2023/3782230 (PMC9844233; doi:10.1155/2023/3782230)
Supplement: Supplementary 1 — Supplementary Figure 1S–48S: the 1H and 13C NMR, MS, and IR spectra of twelve phenyl ketone compounds. [file 3782230.f1.pdf]

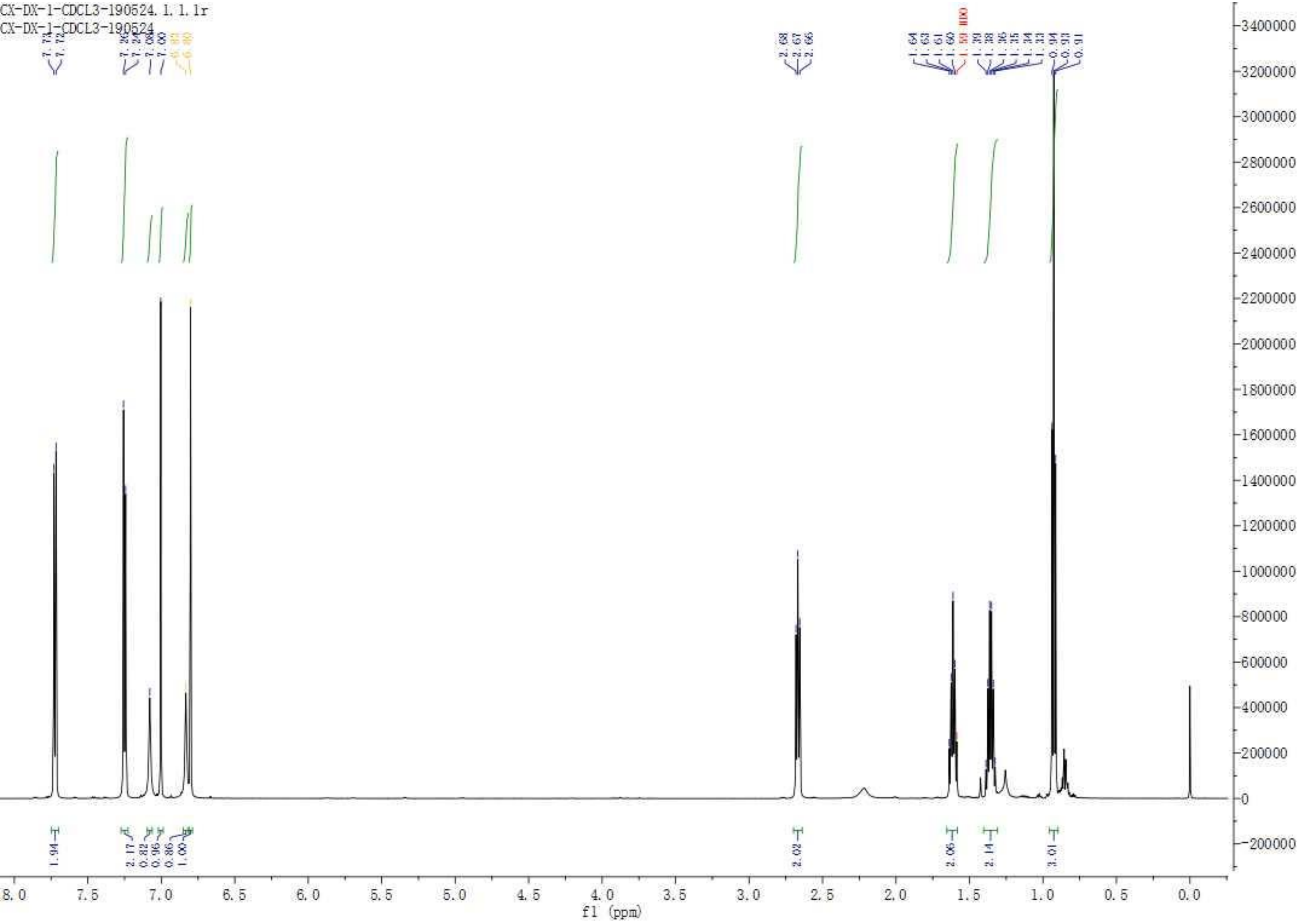

Figure 1S ^{1}H-NMR of compound 5a

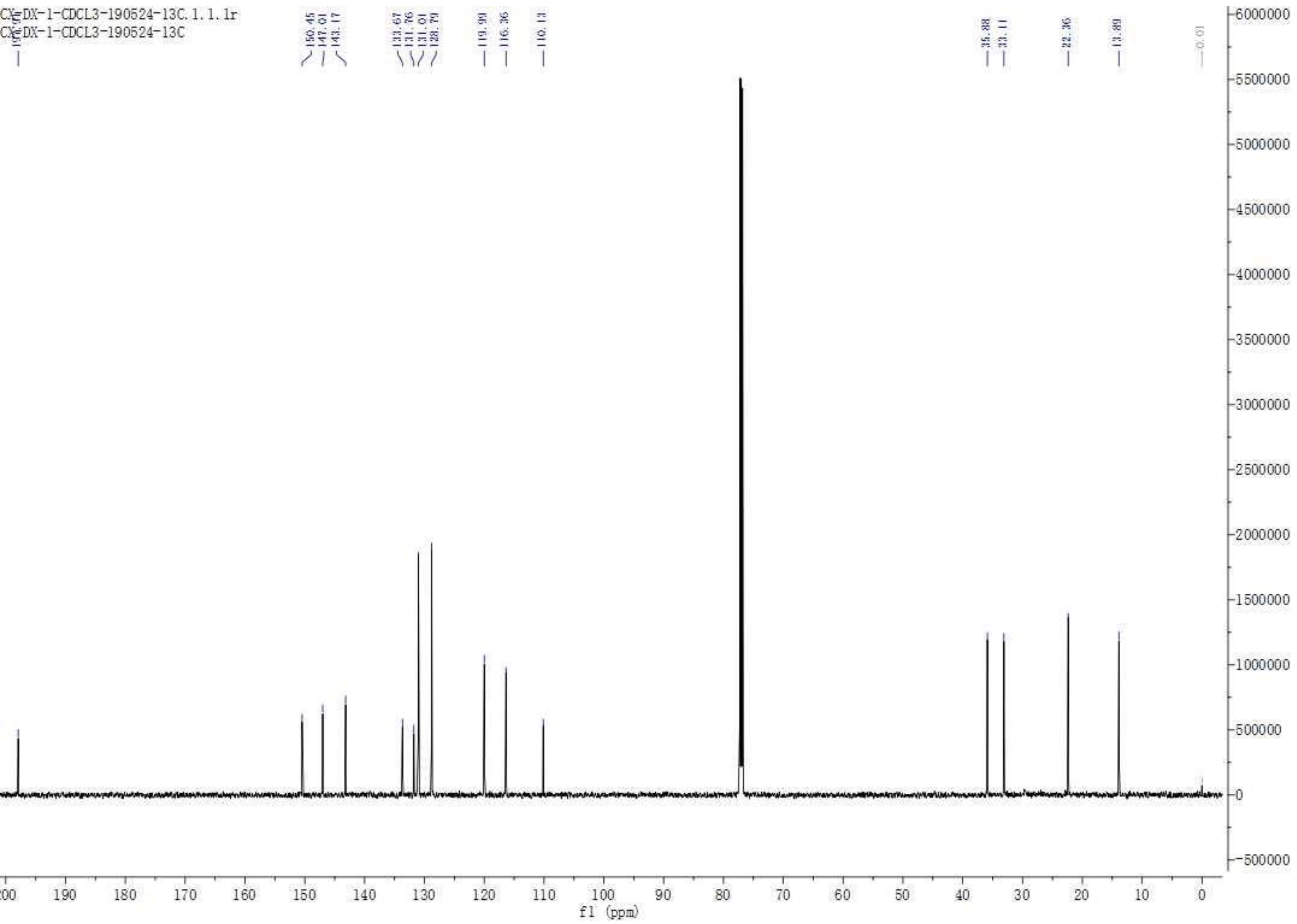

Figure 2S ^{13}C-NMR of compound 5a

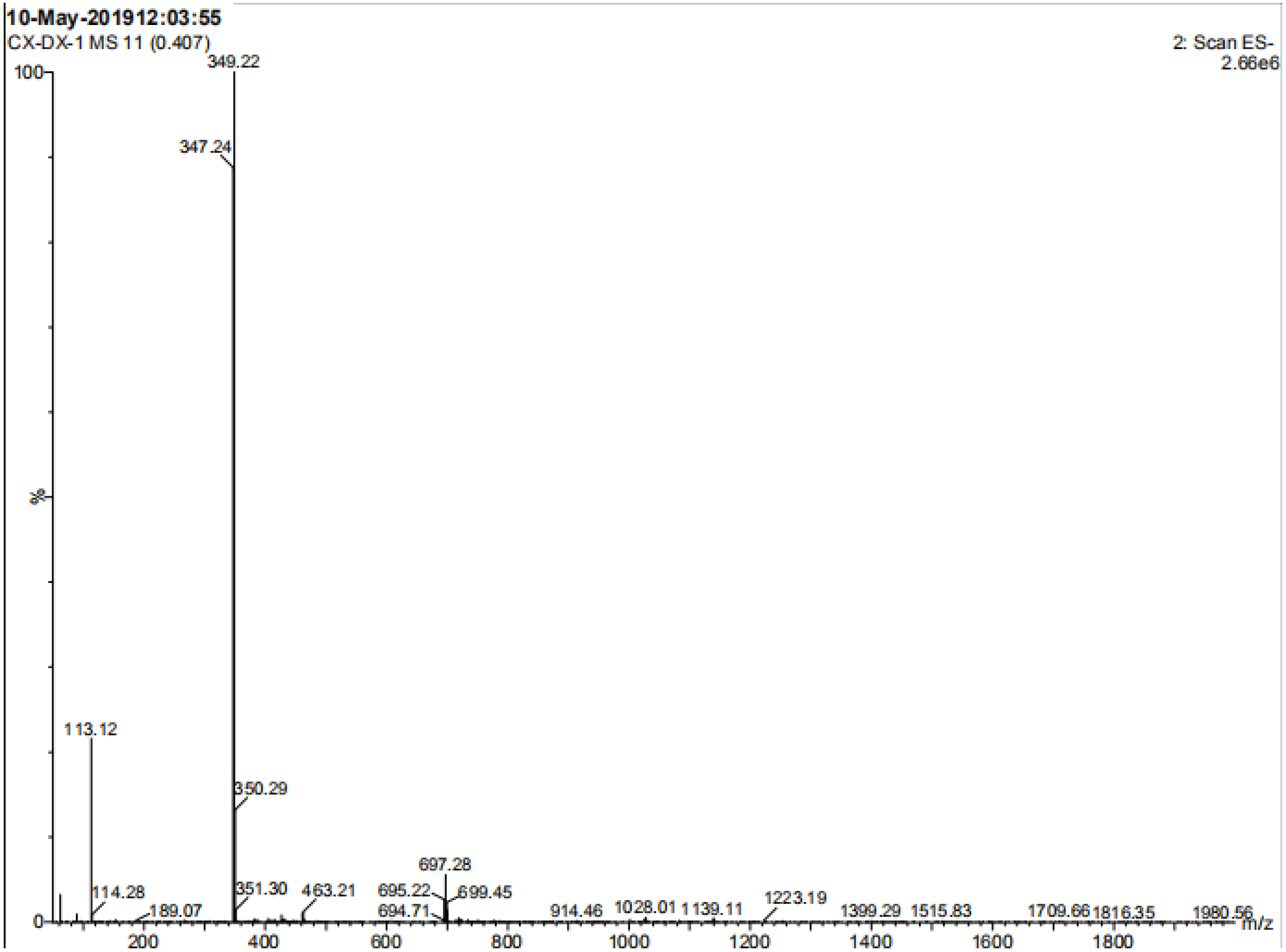

Figure 3S ESI-MS of compound 5a

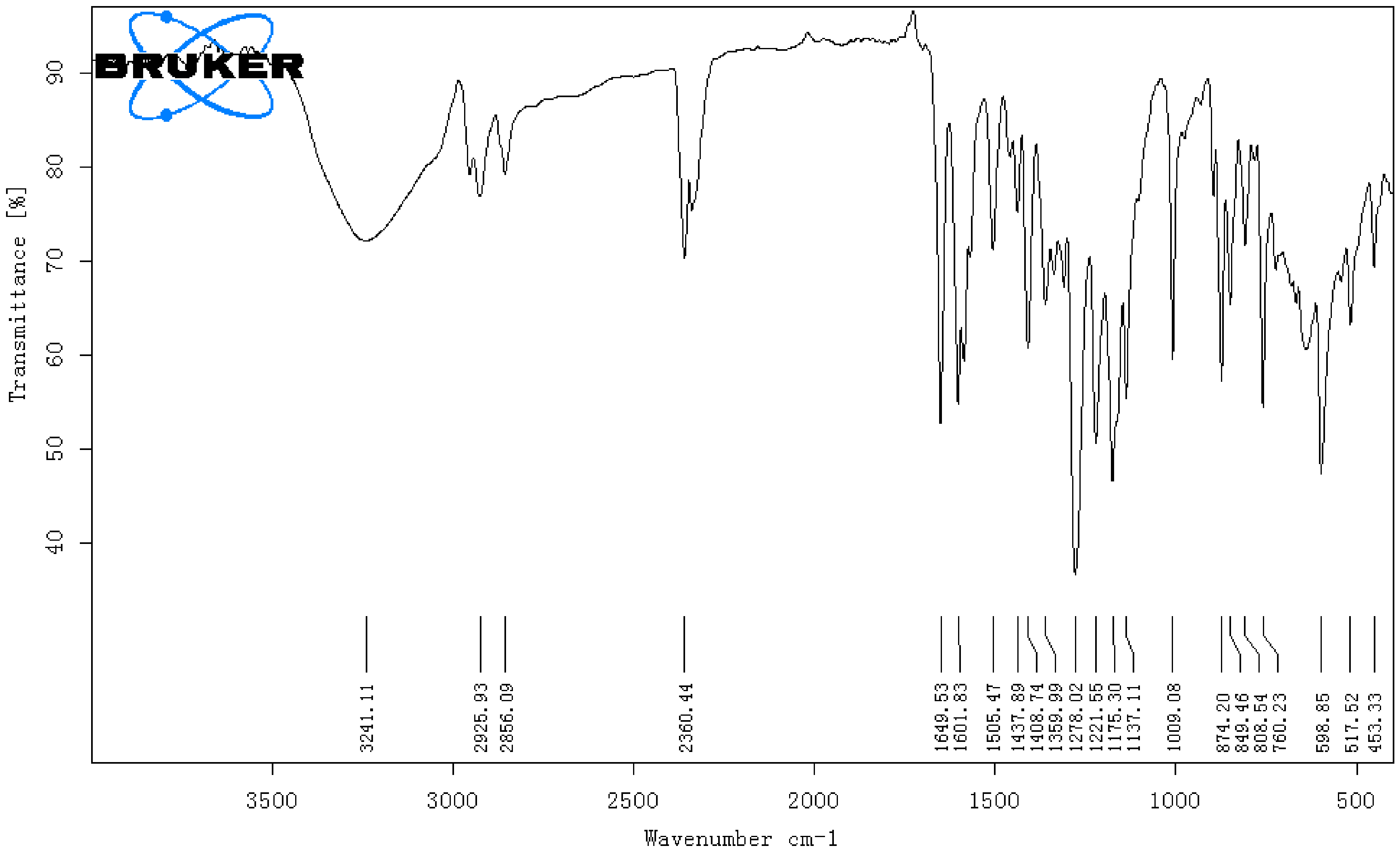

Figure 4S IR of compound 5a

- (2-bromo-4, 5-dihydroxy-phenyl)-(4-butyl-phenyl)-methanone (5a): Green solid. Yield: 45.5%. M.P.: 88.0-89.2 ℃. ^{1}H NMRδ/ppm (400 MHz, d_6-DMSO): 7.72 (d, *J* = 8.2 Hz, 2H, Ar H), 7.25 (d, *J* = 8.5 Hz, 2H, Ar H), 7.08 (s, 1H, Ar H), 7.00 (s, 1H, Ar H), 6.83 (s, 1H, OH), 6.80 (s, 1H, OH), 2.70–2.64 (m, 2H, CH_2), 1.62 (q, *J* = 7.6 Hz, 2H, CH_2), 1.36 (h, *J* = 7.4 Hz, 2H, CH_2), 0.93 (t, *J* = 7.4 Hz, 3H, CH_3). ^{13}C NMRδ/ppm (101 MHz, d_6-DMSO):197.91 (CO), 150.45 (Ar C), 147.01 (Ar C), 143.17 (Ar C), 133.67 (Ar C), 131.76 (Ar C), 131.01 (Ar C), 128.79 (Ar C), 119.99 (Ar C), 116.36 (Ar C), 110.13 (Ar C), 35.88 (CH_2), 33.11 (CH_2), 22.36 (CH_2), 13.89 (CH_3). MS (M^-):349.22. IR cm^{-1}: 3241 br m, 2360 m, 1649 s, 1601 s, 1278 s, 1175 s, 598s.

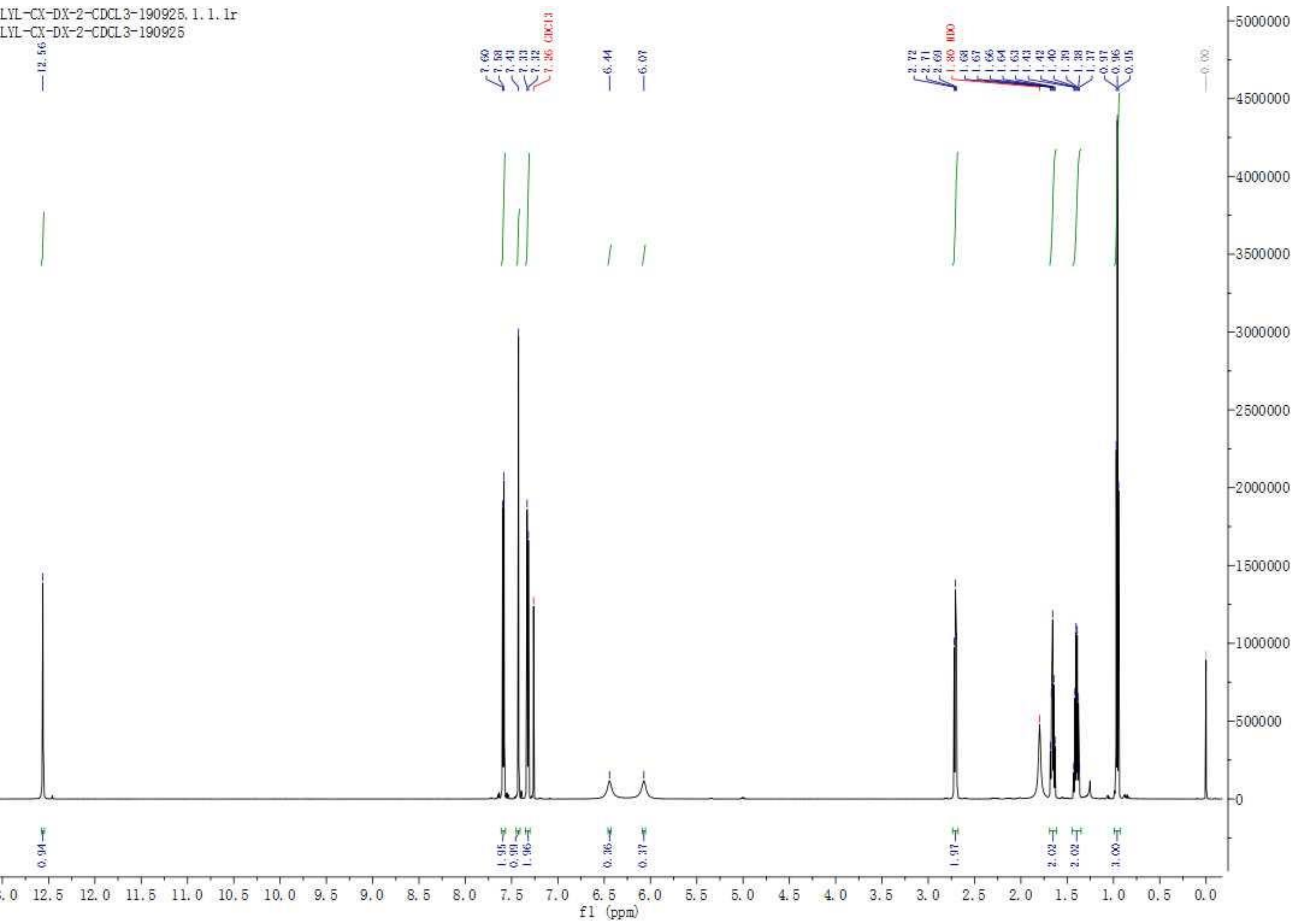

Figure 5S ^{1}H-NMR of compound 5b

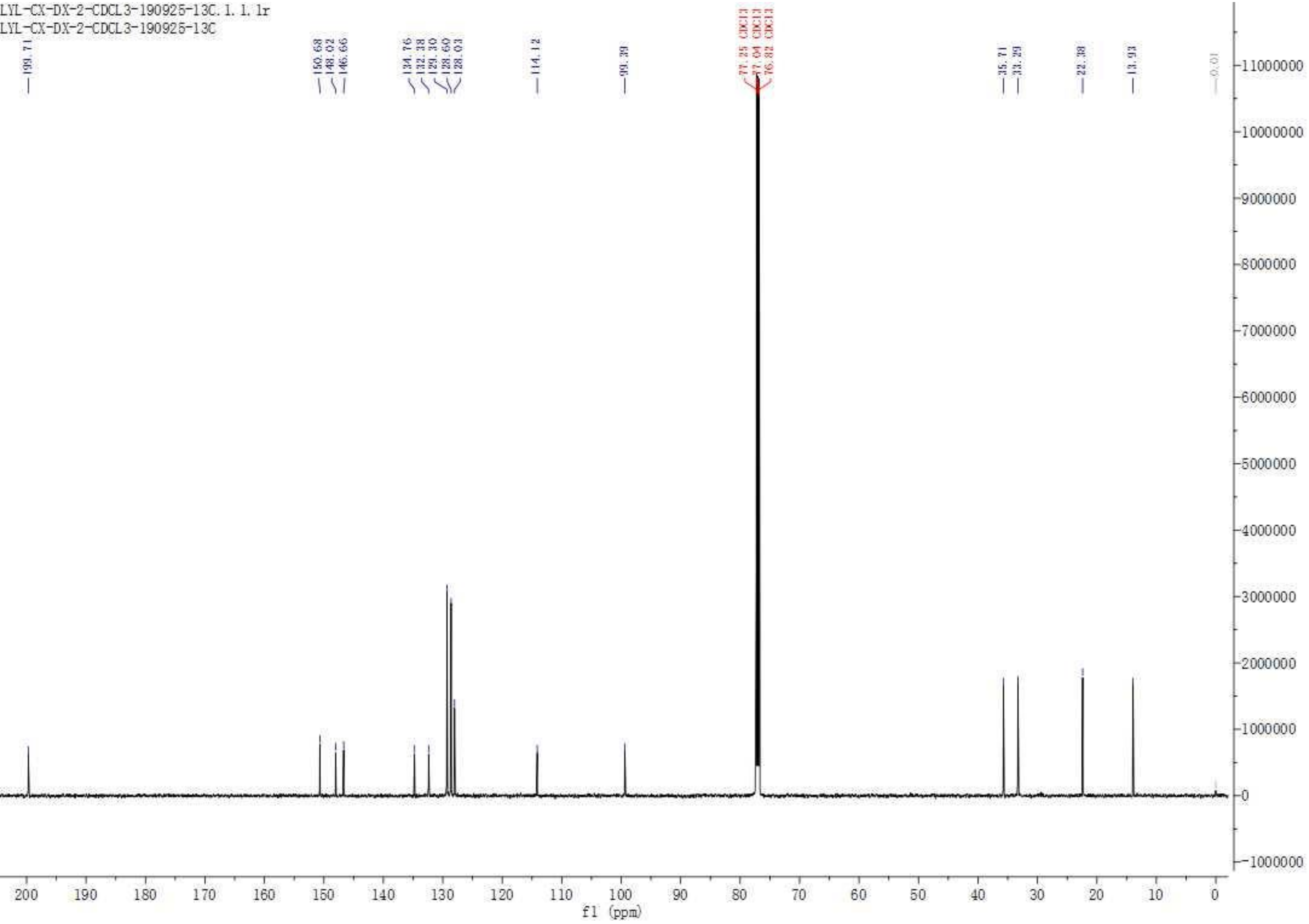

Figure 6S ^{13}C-NMR of compound 5b

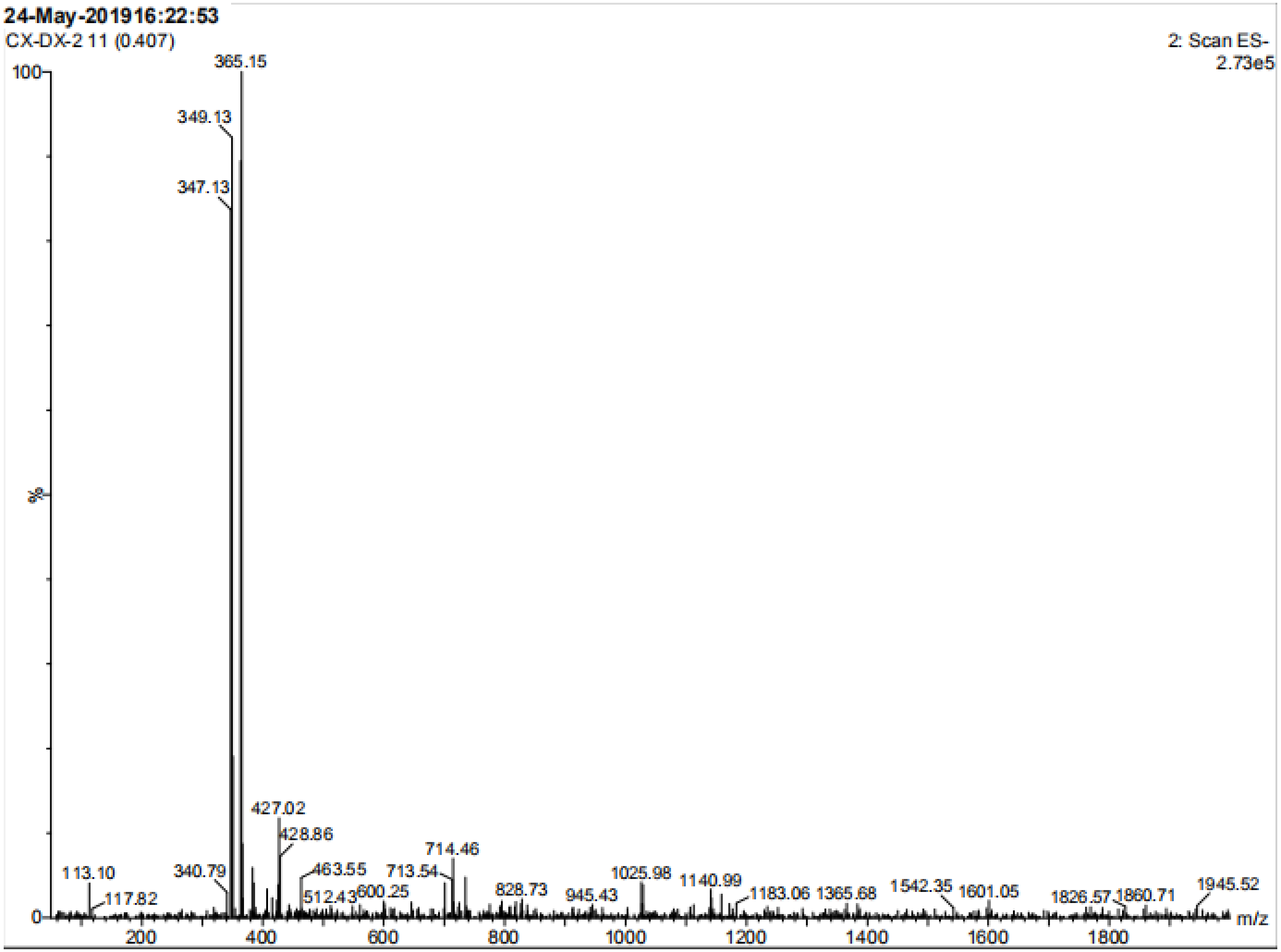

Figure 7S ESI-MS of compound 5b

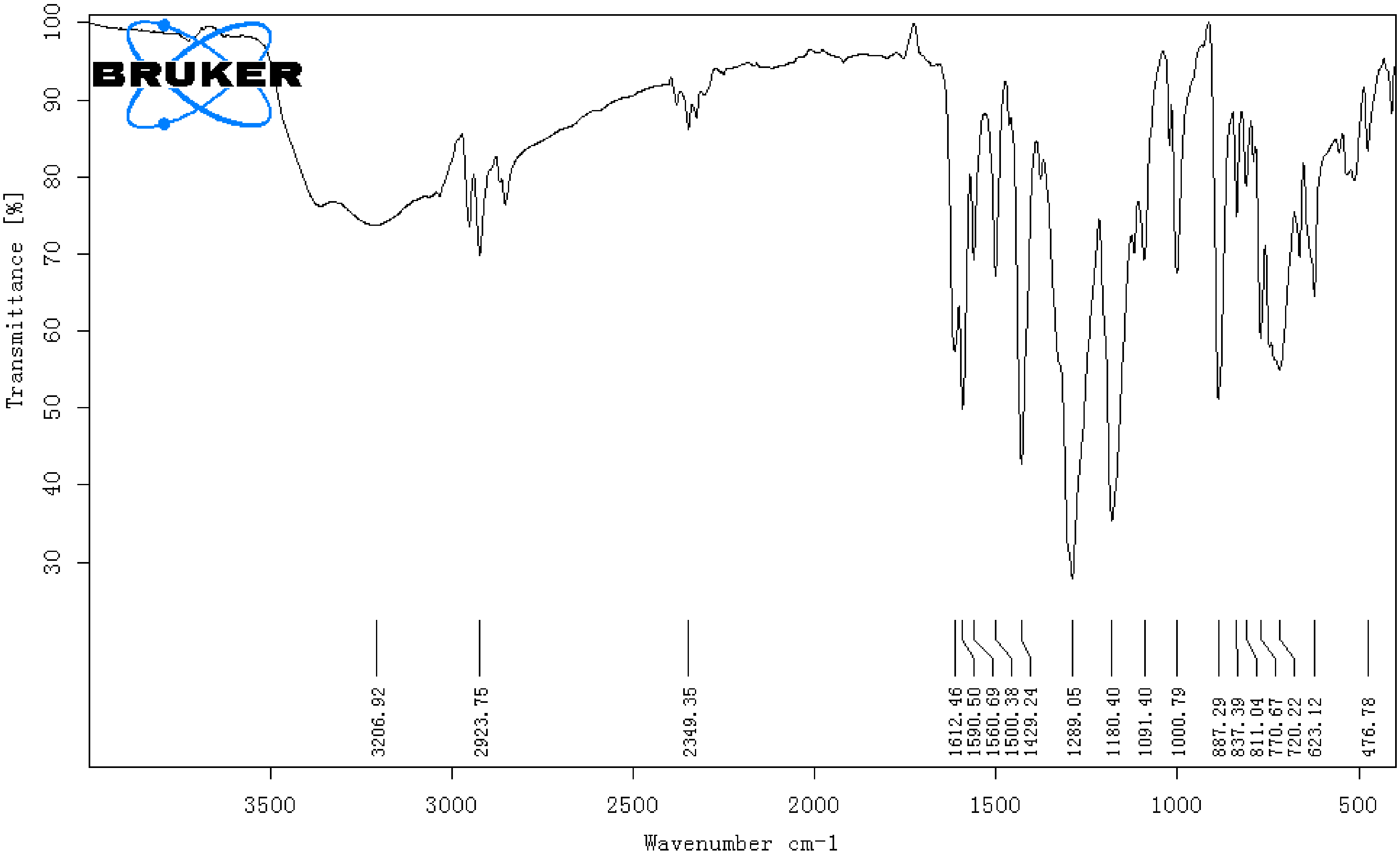

Figure 8S IR of compound 5b

- (6-bromo-2, 3, 4-trihydroxy-phenyl)-(4-butyl-phenyl)-methanone (5b): Brown solid. Yield: 47.5%. M.P.: 58.0-59.2 ℃. ^{1}H NMRδ/ppm (400 MHz, d_6-DMSO): 12.56 (s, 1H, OH), 7.59 (d, *J* = 8.2 Hz, 2H, Ar H), 7.43 (s, 1H, Ar H), 7.33 (d, *J* = 8.2 Hz, 2H, Ar H), 6.44 (s, 1H, OH), 6.07 (s, 1H, OH), 2.74–2.68 (m, 2H, CH_2), 1.66 (p, *J* = 7.6 Hz, 2H, CH_2), 1.40 (h, *J* = 7.4 Hz, 2H, CH_2), 0.96 (t, *J* = 7.4 Hz, 3H, CH_3). ^{13}C NMRδ/ppm (101 MHz, d_6-DMSO): 199.71 (CO), 150.68 (Ar C), 148.02 (Ar C), 146.66 (Ar C), 134.76 (Ar C), 132.38 (Ar C), 129.30 (Ar C), 128.60 (Ar C), 128.03 (Ar C), 114.12 (Ar C), 99.39 (Ar C), 35.71 (CH_2), 33.29 (CH_2), 22.38 (CH_2), 13.93 (CH_3).MS (M^-):365.15. IR cm^{-1}: 3206 br m, 1590 s, 1429 s, 1289 s, 1180 s, 887 s.

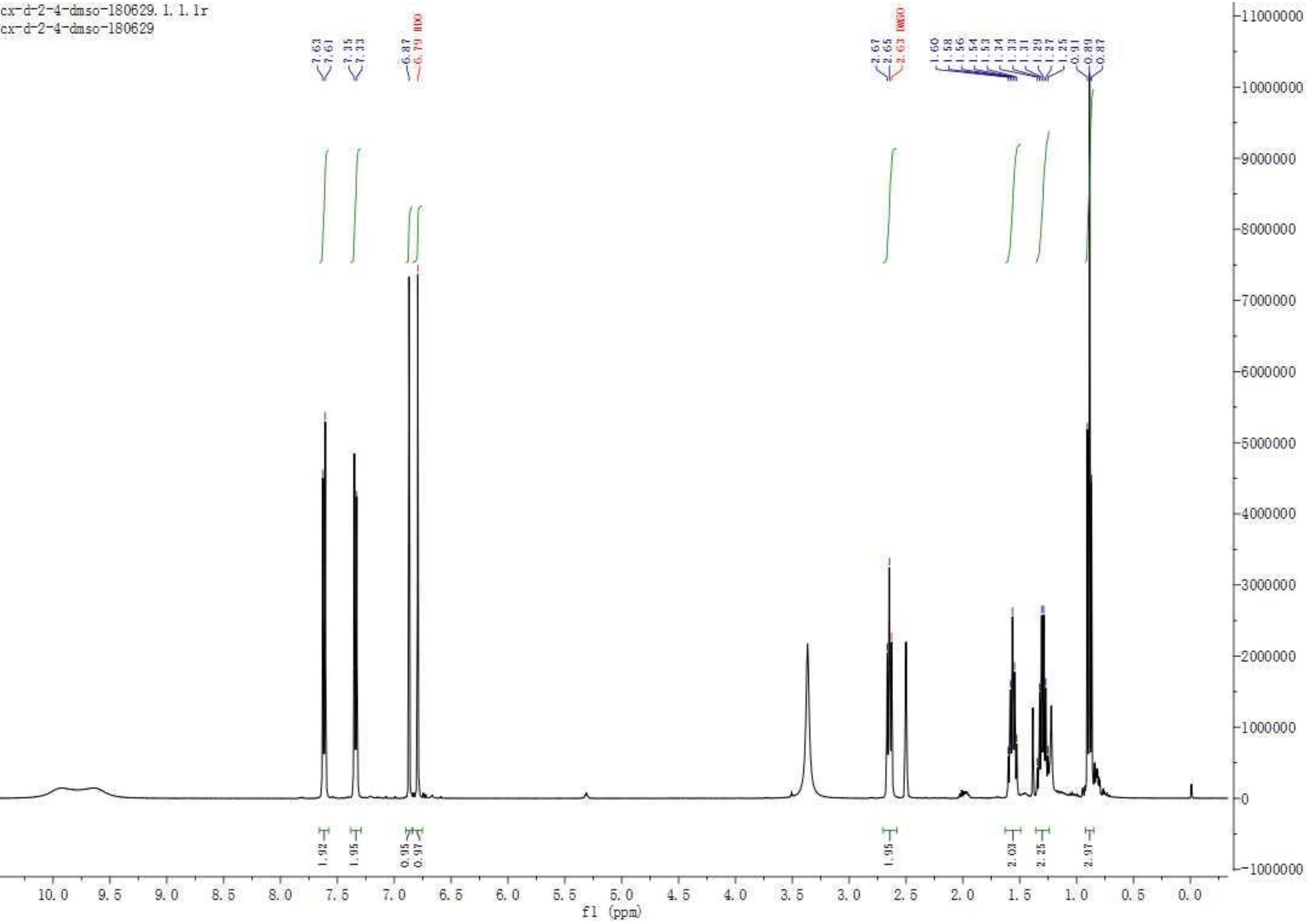

Figure 9S ¹H-NMR of compound 5c

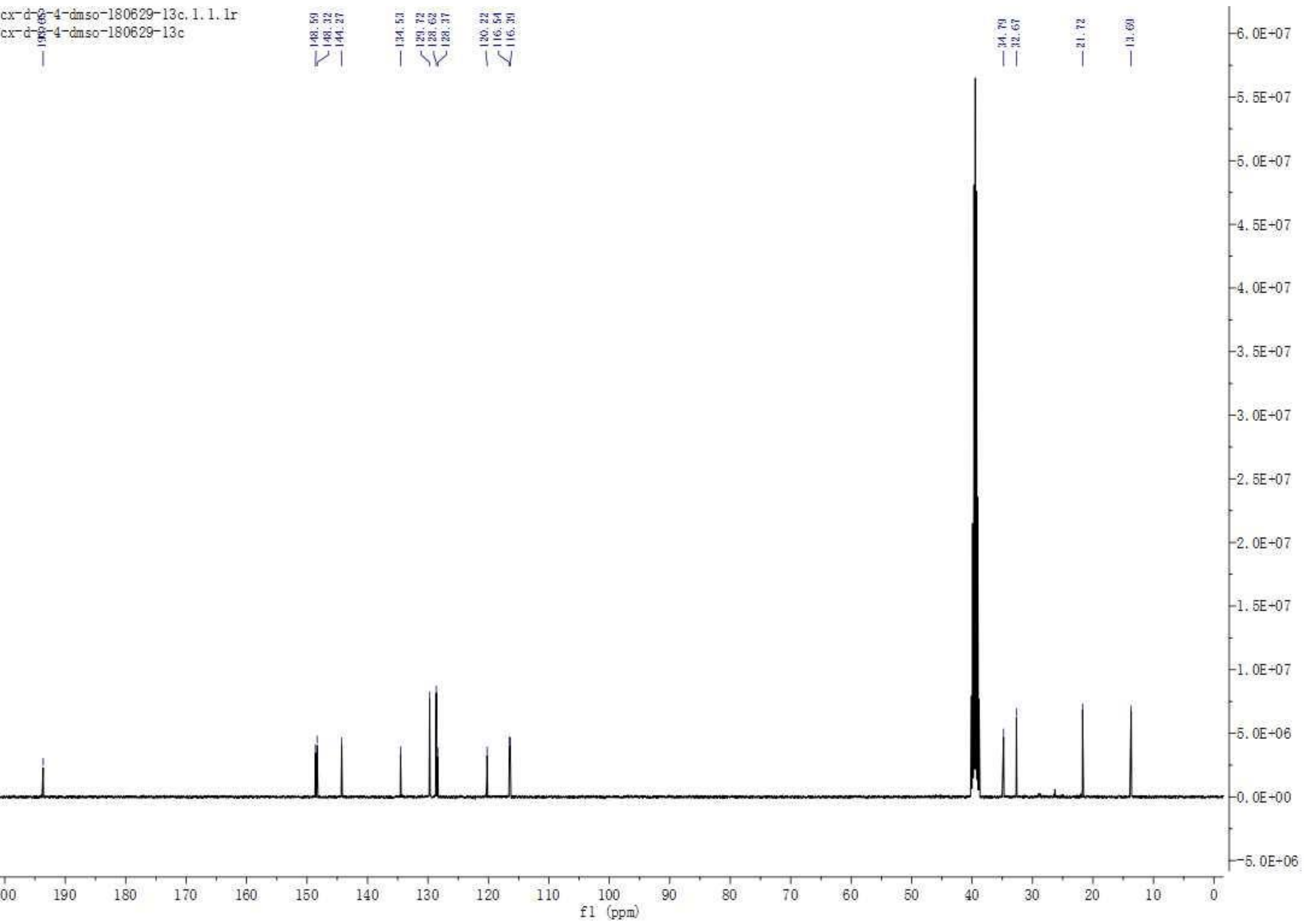

Figure 10S ^{13}C-NMR of compound 5c

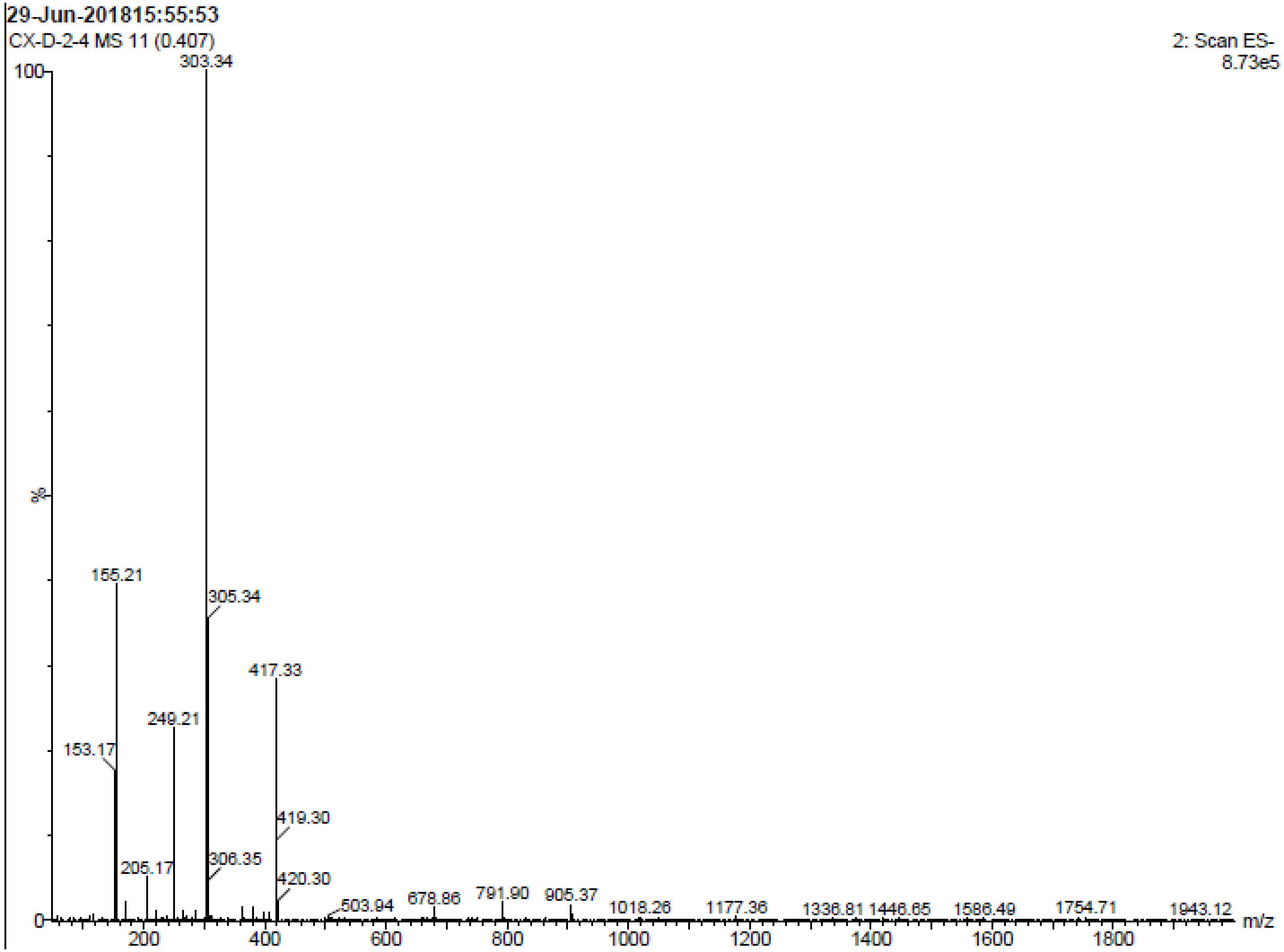

Figure 11S ESI-MS of compound 5c

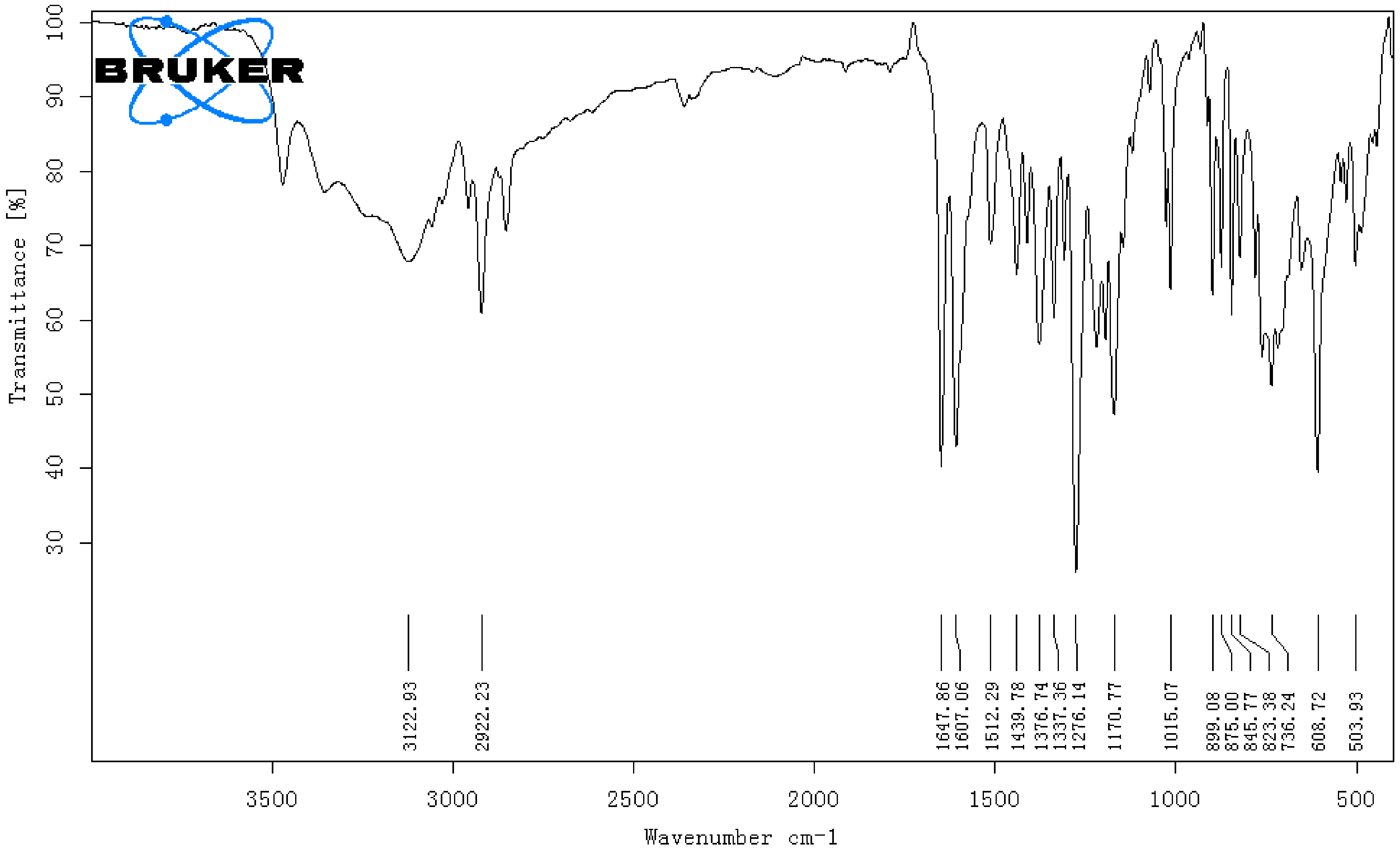

Figure 12S IR of compound 5c

- (4-butyl-phenyl)-(2-chloro-4, 5-dihydroxy-phenyl)-methanone (5c): Brown solid. Yield: 47.2%. M.P.: 54.8-55.2 ℃. ^{1}H NMR δ/ppm (400 MHz, d_6-DMSO): 7.62 (d, J = 8.1 Hz, 2H, Ar H), 7.34 (d, J = 7.9 Hz, 2H, Ar H), 6.87 (d, 2H, Ar H), 2.66 (d, J = 7.7 Hz, 2H, CH_2), 1.56 (p, J = 7.5 Hz, 2H, CH_2), 1.30 (h, J = 7.4 Hz, 2H, CH_2), 0.89 (t, J = 7.3 Hz, 3H, CH_3). ^{13}C NMR δ/ppm (101 MHz, d_6-DMSO): 193.65 (CO), 148.59 (Ar C), 148.32 (Ar C), 144.27 (Ar C), 134.53 (Ar C), 129.72 (Ar C), 128.62 (Ar C), 128.37 (Ar C), 120.22 (Ar C), 116.54 (Ar C), 116.39 (Ar C), 34.79 (CH_2), 32.67 (CH_2), 21.72 (CH_2), 13.69 (CH_3). MS (M^-): 303.34. IR cm^{-1}: 3122 br w, 1647 m, 1607 m, 1276 s, 608 m.

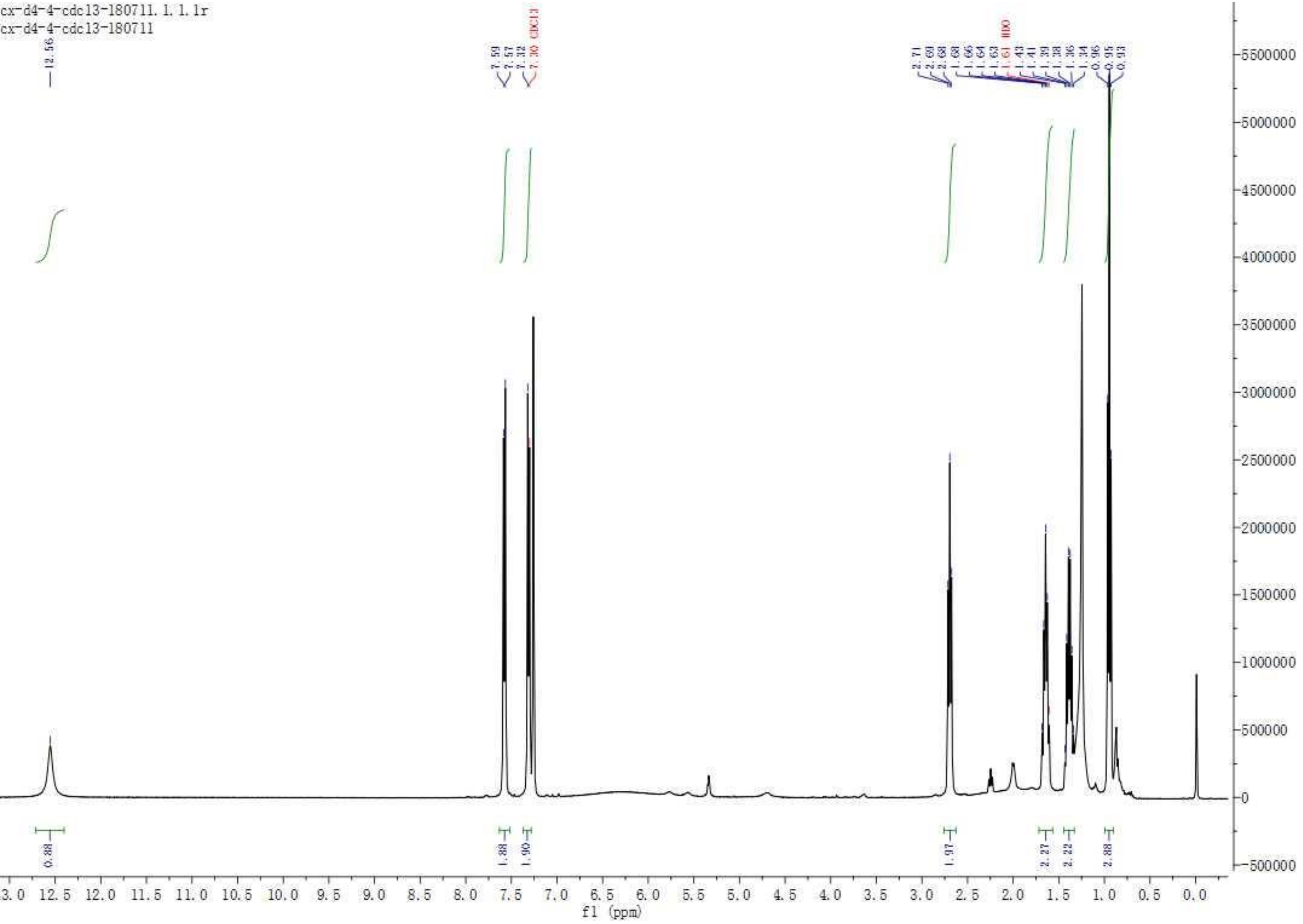

Figure 13S ¹H-NMR of compound 5d

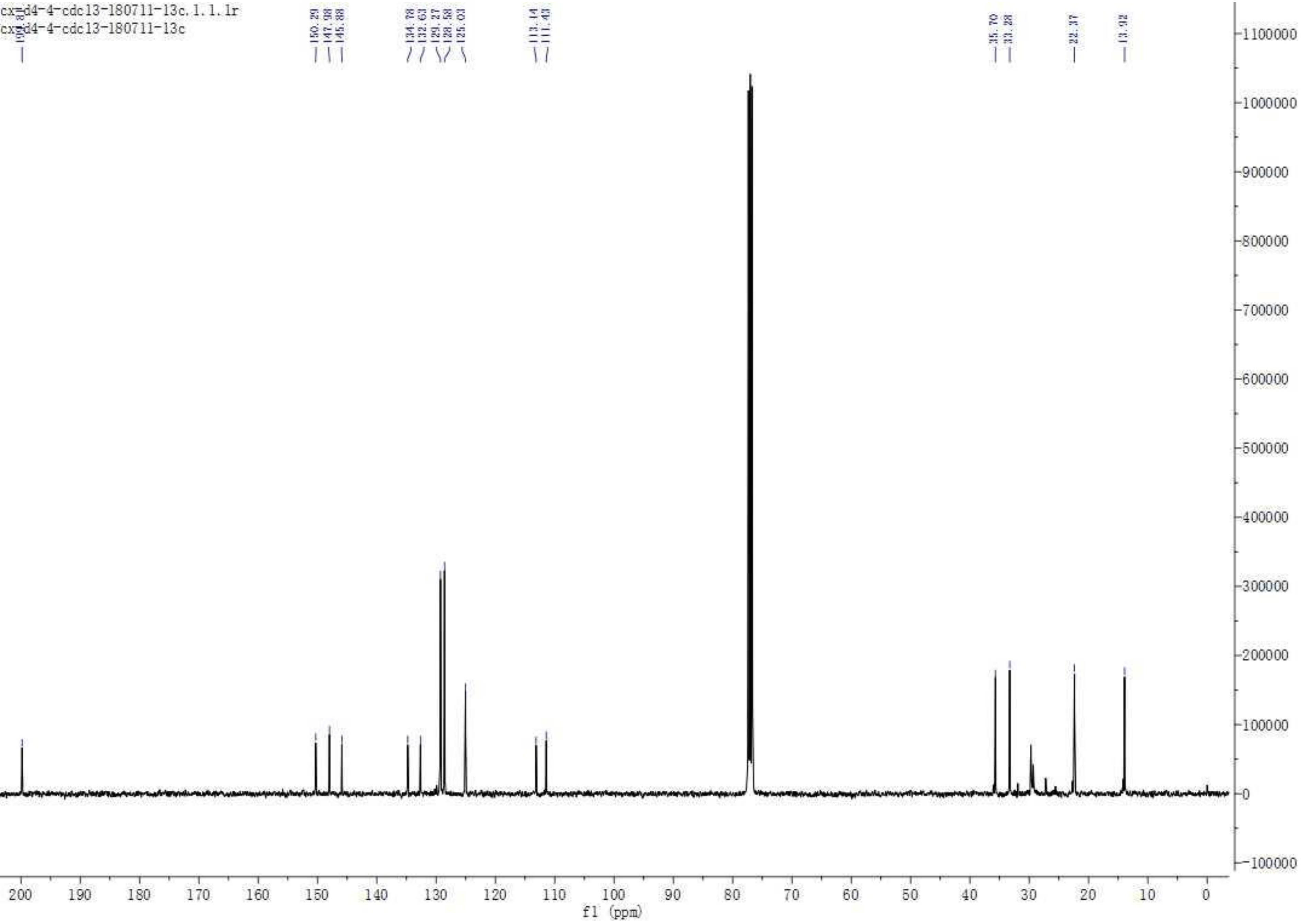

Figure 14S ^{13}C-NMR of compound 5d

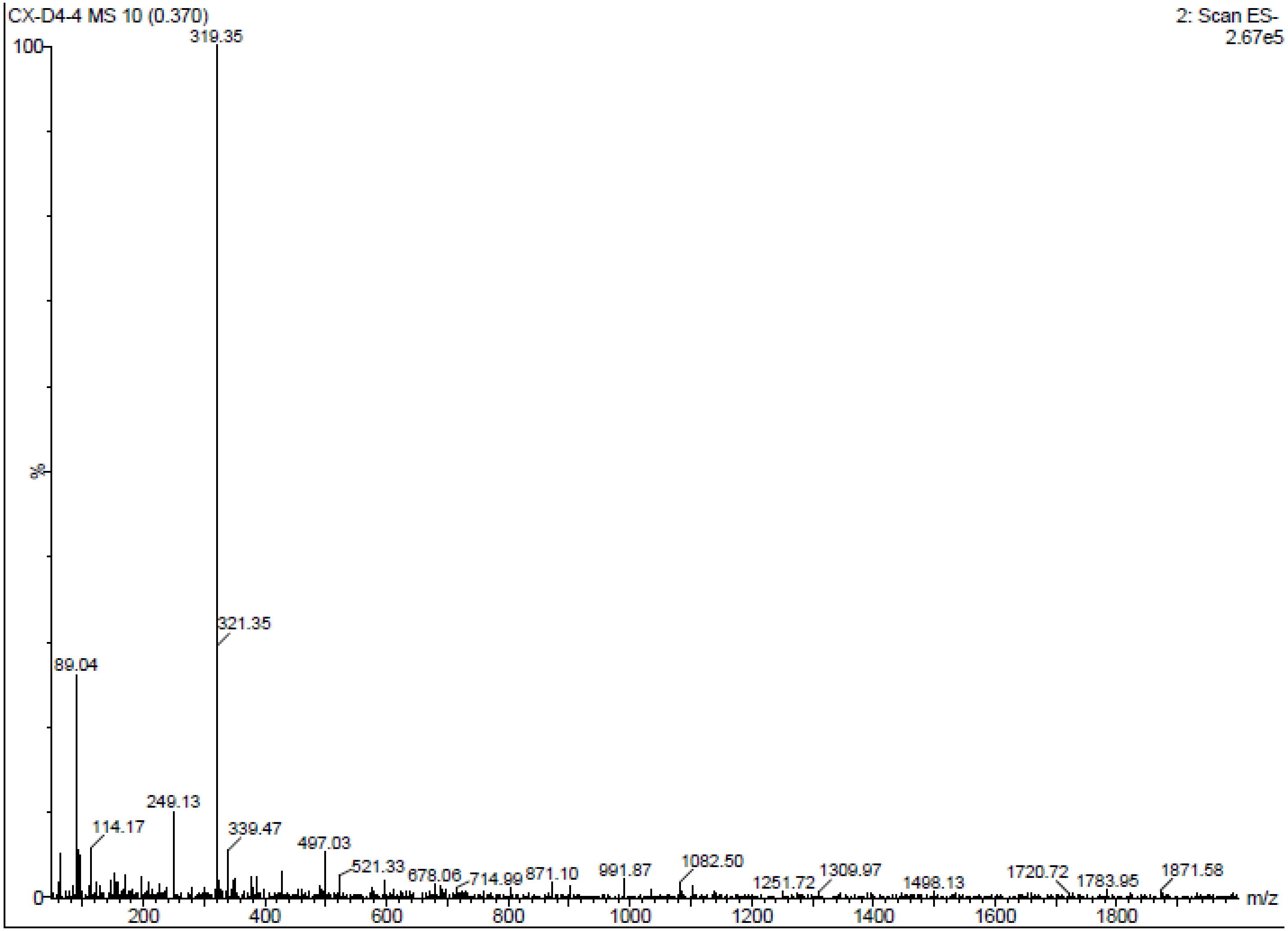

Figure 15S ESI-MS of compound 5d

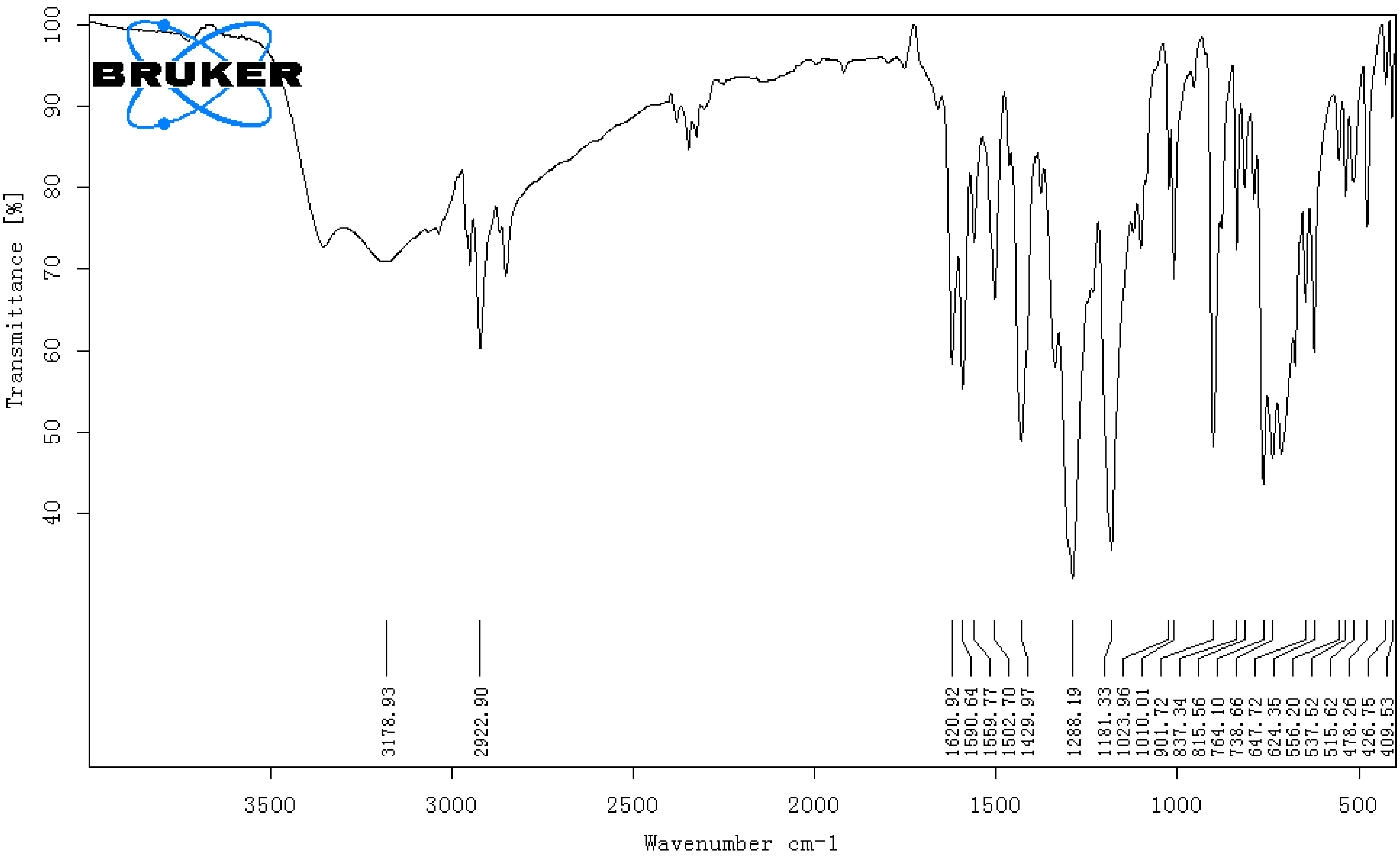

Figure 16S IR of compound 5d

- (4-butyl-phenyl)-(6-chloro-2, 3, 4-trihydroxy-phenyl)-methanone (5d): Yellow solid. Yield: 42.1%. M.P.: 115.2-115.9 ℃. ^{1}H NMRδ/ppm (400 MHz, d_6-DMSO): 12.56 (s, 1H, OH), 7.58 (d, *J* = 7.9 Hz, 2H, Ar H), 7.32 (s, 2H, Ar H), 7.26 (s, 1H , Ar H), 2.69 (t, *J* = 7.7 Hz, 2H, CH_2), 1.72–1.57 (m, 2H, CH_2), 1.38 (q, *J* = 14.7, 7.3 Hz, 2H, CH_2), 0.95 (t, *J* = 7.3 Hz, 3H, CH_3). ^{13}C NMRδ/ppm (101 MHz, d_6-DMSO): 199.81 (CO), 150.29 (Ar C), 147.98 (Ar C), 145.88 (Ar C), 134.78 (Ar C), 132.63 (Ar C), 129.27 (Ar C), 128.58 (Ar C), 125.03 (Ar C), 113.14 (Ar C), 111.43 (Ar C), 35.70 (CH_2), 33.28 (CH_2), 22.37 (CH_2), 13.92 (CH_3). MS (M^-):319.35. IR cm^{-1}: 3178 br m, 1620 s, 1288 s, 1181 s, 647 s.

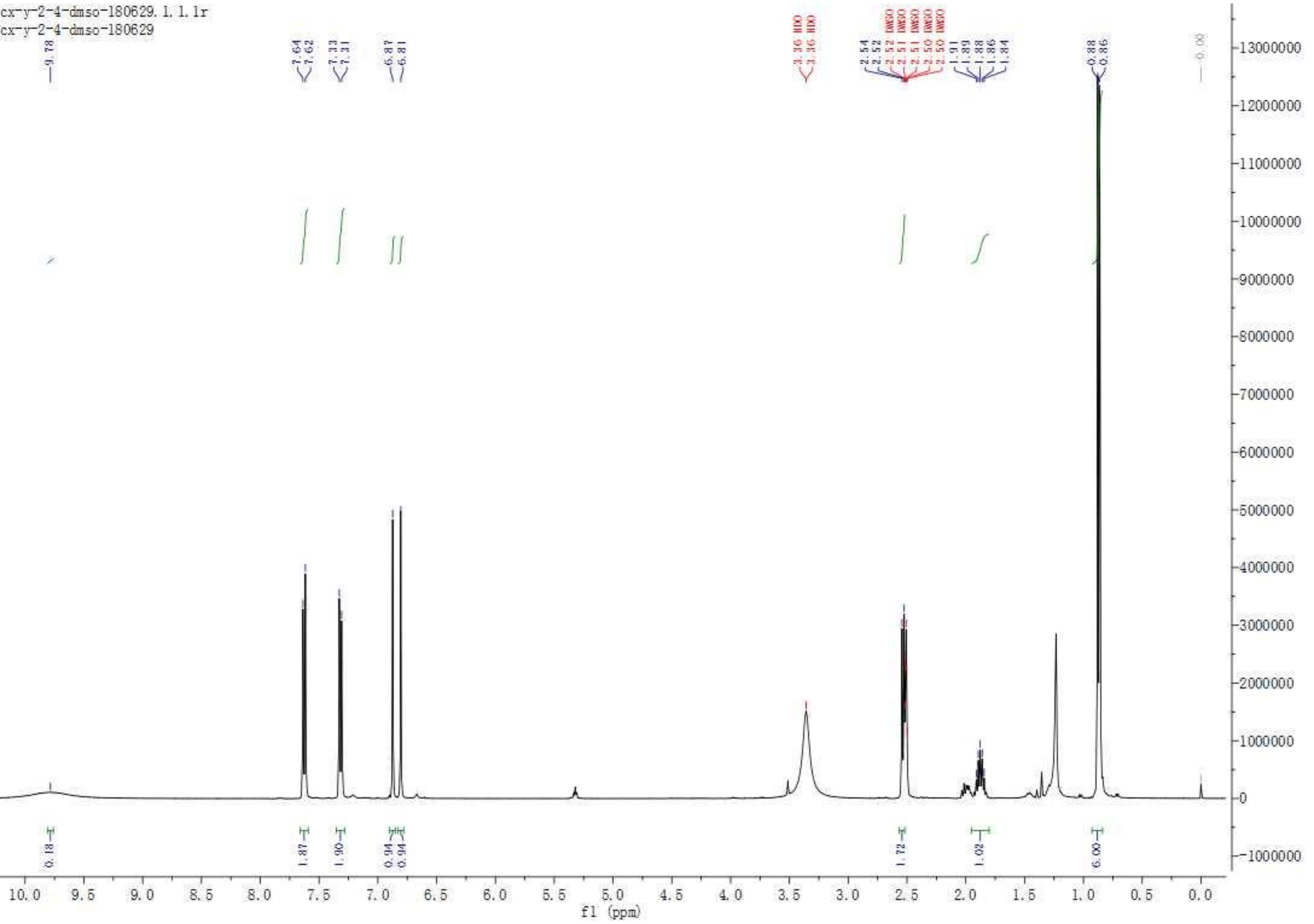

Figure 17S ¹H-NMR compound 5e

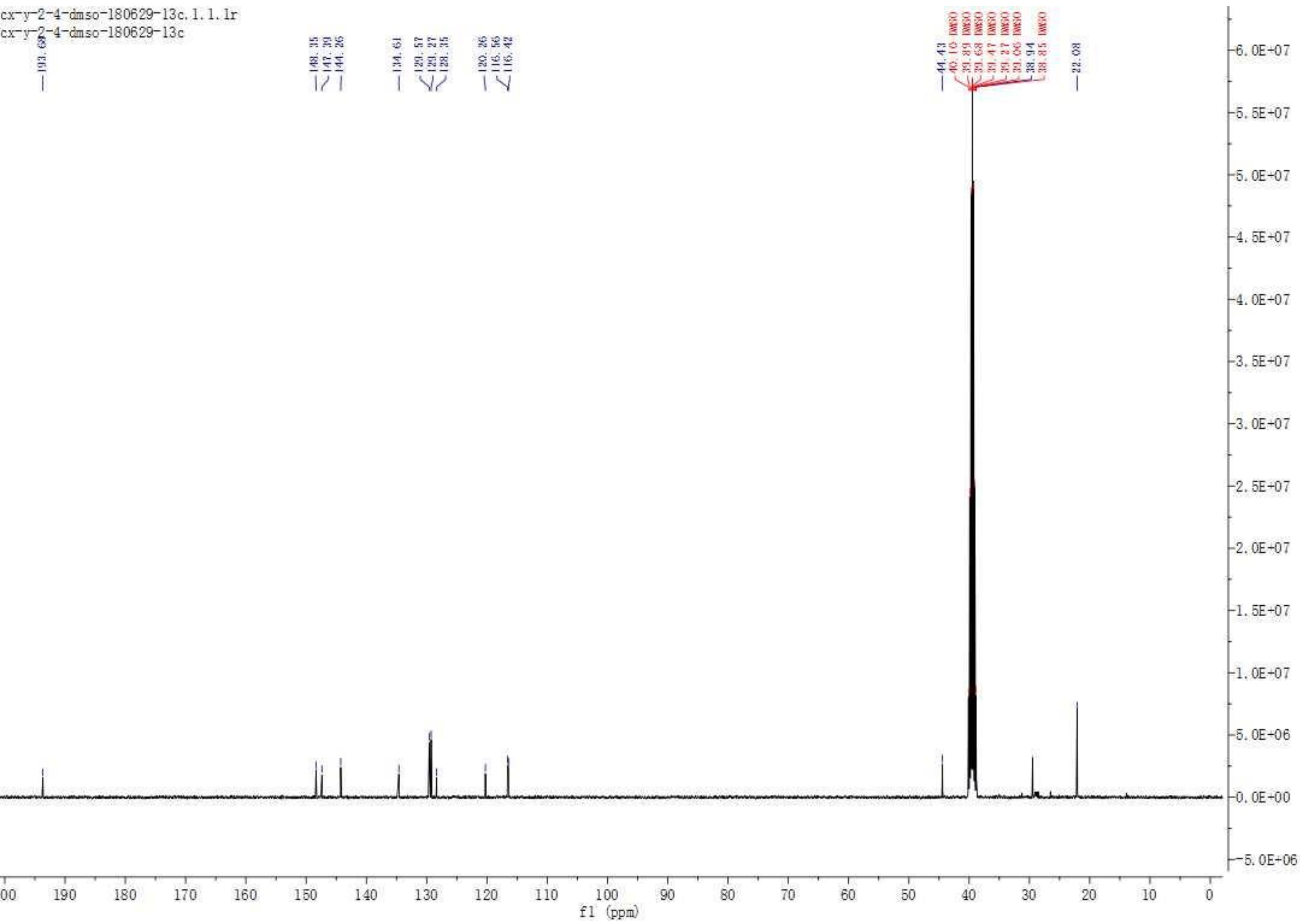

Figure 18S ^{13}C-NMR of compound 5e

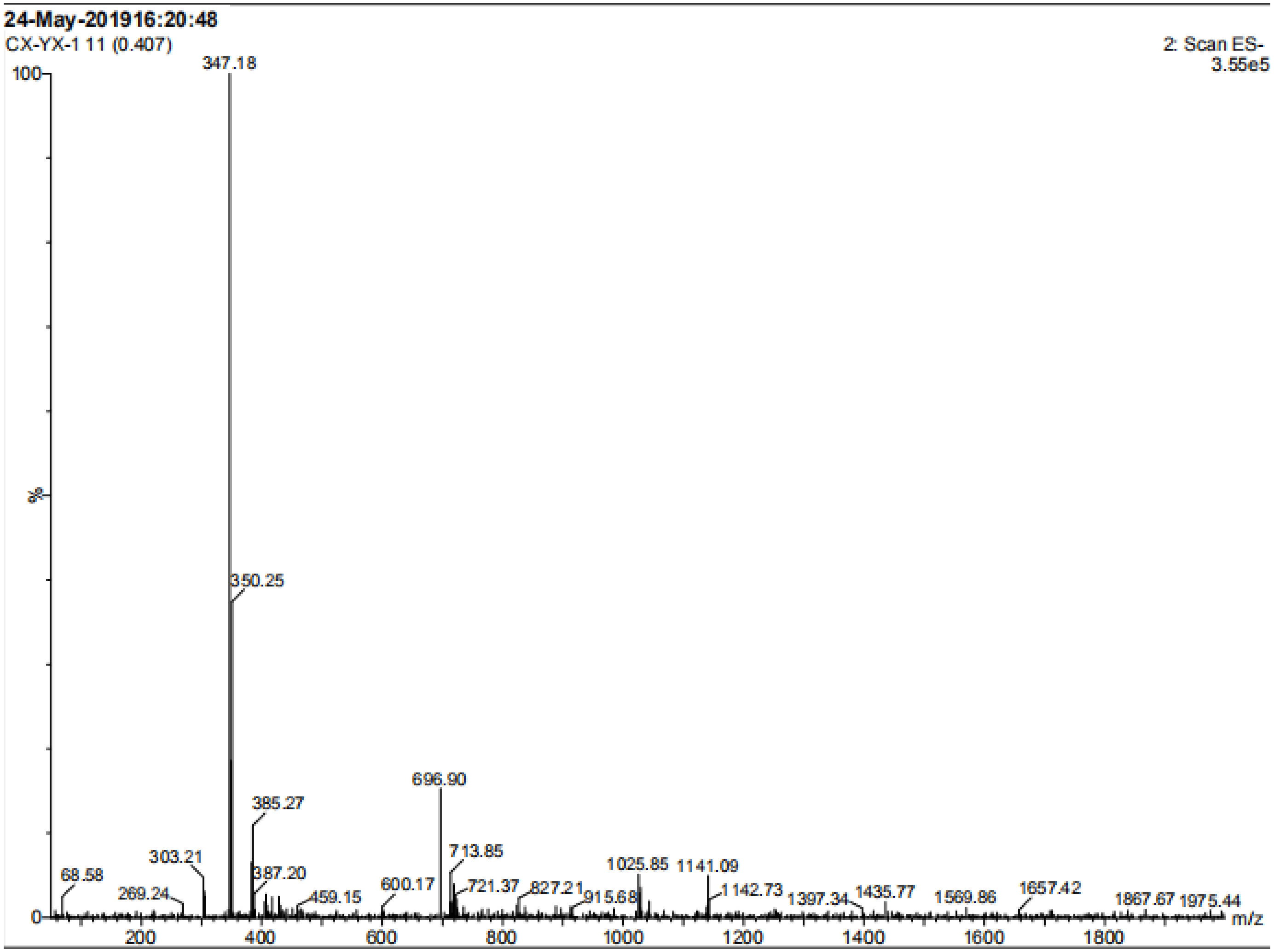

Figure 19S ESI-MS of compound 5e

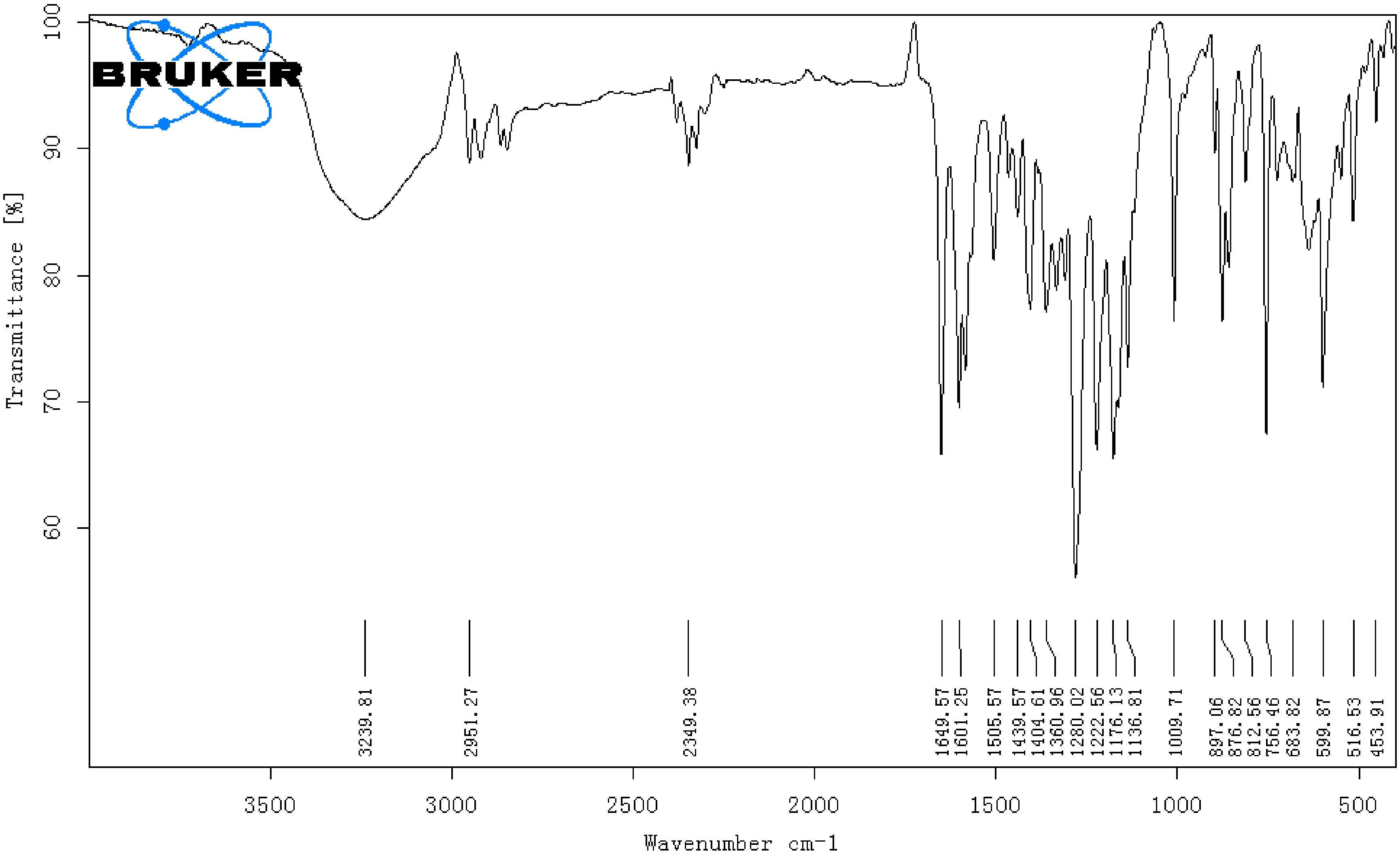

Figure 20S IR of compound 5e

- (2-bromo-4, 5-dihydroxy-phenyl)-(4-isobutyl-phenyl)-methanone (5e): Brown solid. Yield: 46.7%. M.P.: 104.2-105.6 ℃. ^{1}H NMRδ/ppm (400 MHz, d_6-DMSO): 9.78 (s, 1H, OH), 7.63 (d, *J* = 8.2 Hz, 2H, Ar H), 7.32 (d, *J* = 8.0 Hz, 2H, Ar H), 6.87 (s, 1H, Ar H), 6.81 (s, 1H, Ar H), 2.53 (d, *J* = 7.2 Hz, 2H, CH_2), 1.88 (h, *J* = 6.8 Hz, 1H, CH), 0.87 (d, *J* = 6.5 Hz, 6H, 2 × CH_3). ^{13}C NMRδ/ppm (101 MHz, d_6-DMSO): 193.68 (CO), 148.35 (Ar C), 147.39 (Ar C), 144.26 (Ar C), 134.61 (Ar C), 129.57 (Ar C), 129.27 (Ar C), 128.35 (Ar C), 120.26 (Ar C), 116.49 (d, *J* = 13.7 Hz, Ar C)), 44.43 (CH_2), 38.94 (CH), 22.08 (CH_3). MS (M^-):347.18. IR cm^{-1}: 3239 br m, 1649 s, 1601 s, 1280 s, 1176 s, 756 s.

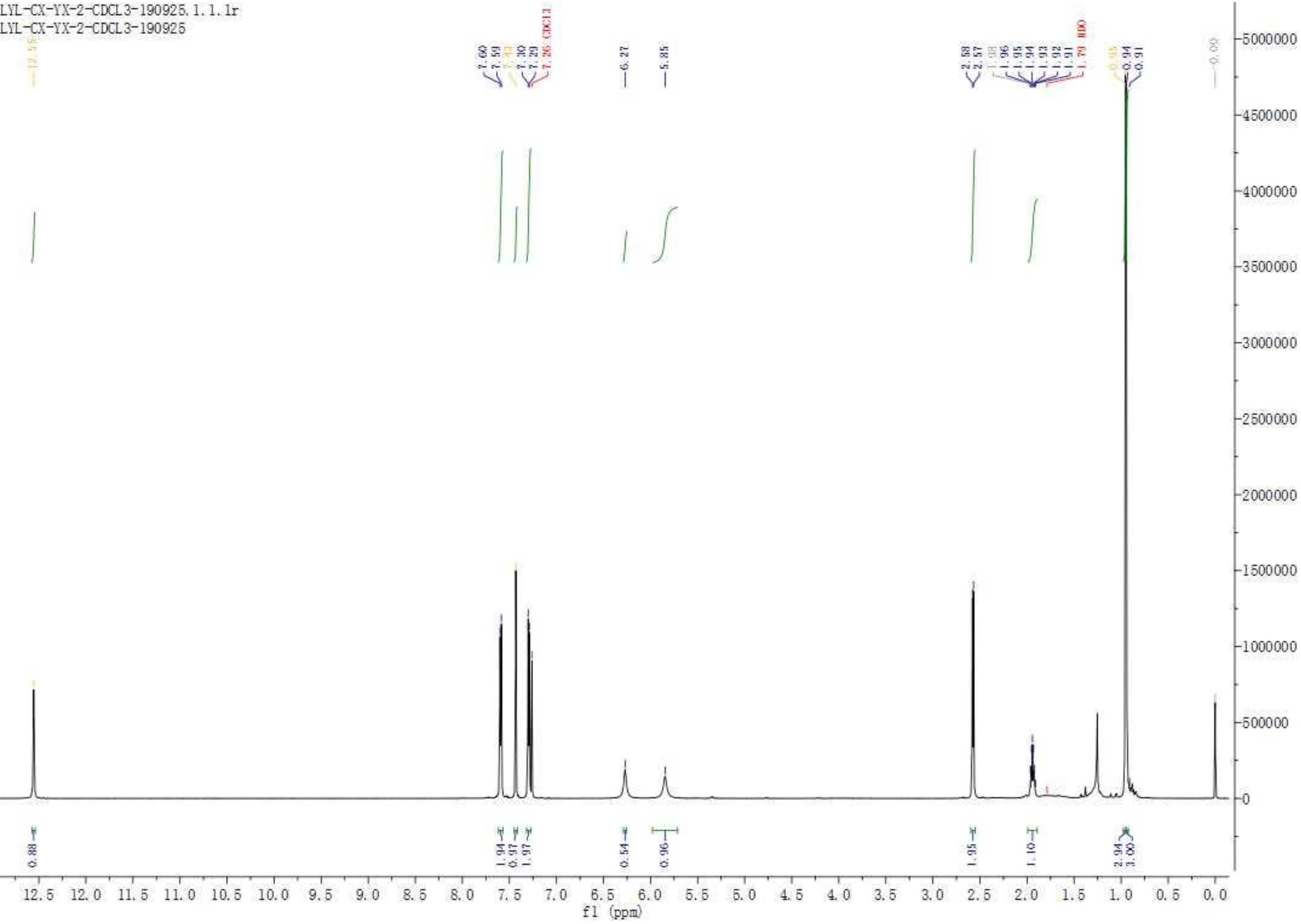

Figure 21S ^{1}H-NMR of compound 5f

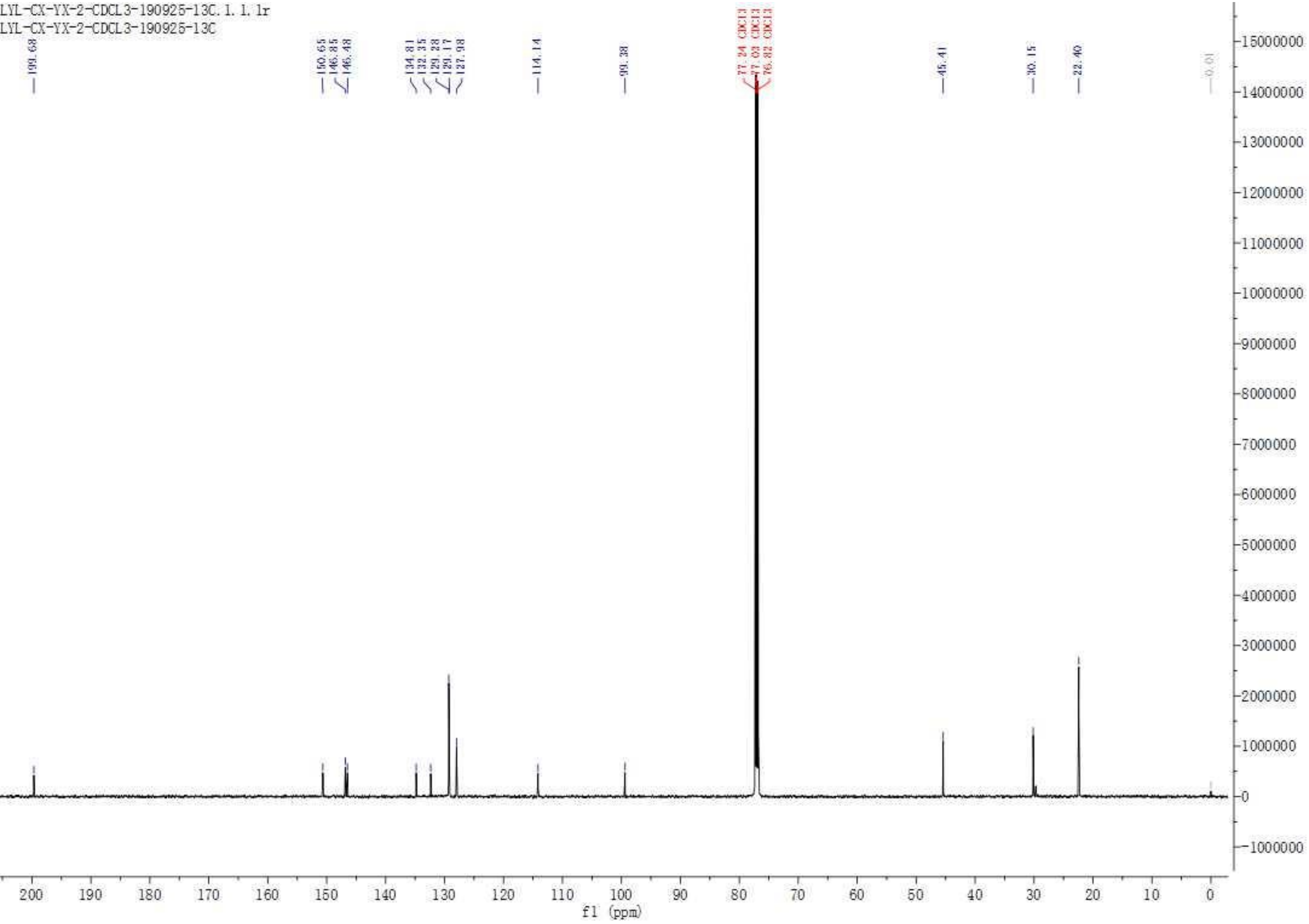

Figure 22S ^{13}C-NMR of compound 5f

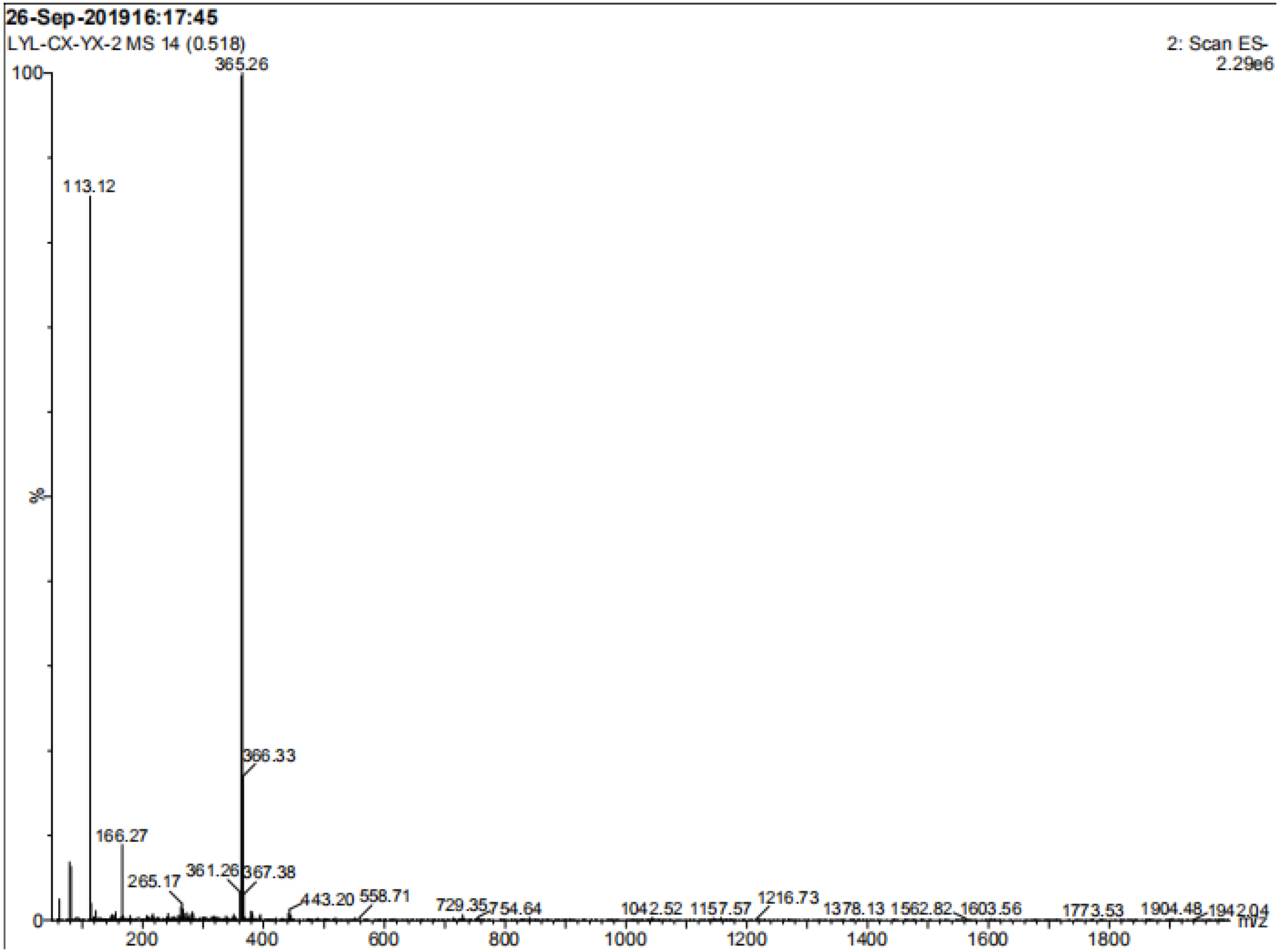

Figure 23S ESI-MS of compound 5f

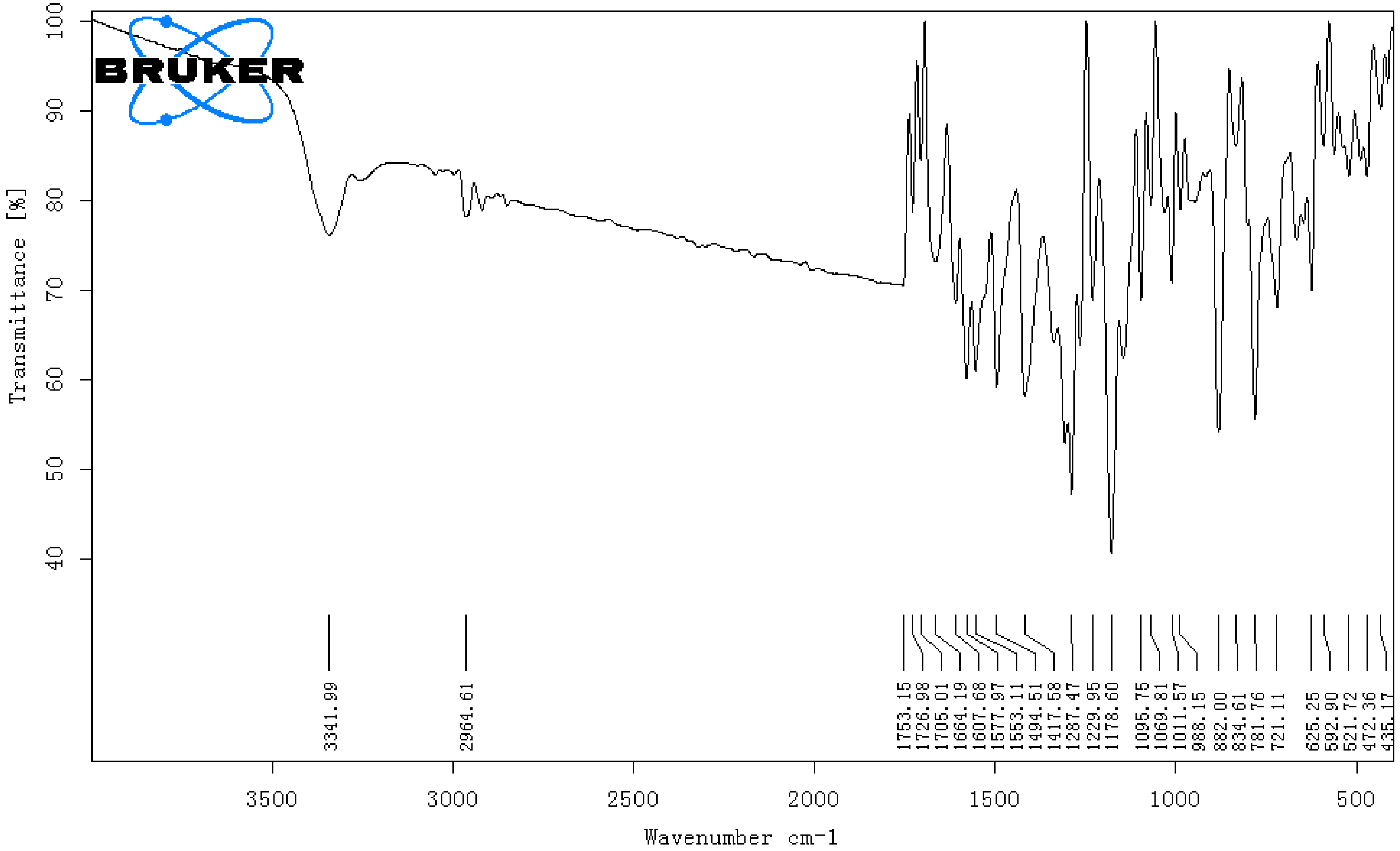

Figure 24S IR of compound 5f

- (6-bromo-2, 3, 4-trihydroxy-phenyl)-(4-isobutyl-phenyl)-methanone (5f): Brown solid. Yield: 45.7%. M.P.: 137.0-138.0 ℃. ^{1}H NMRδ/ppm (400 MHz, d_6-DMSO): 12.55 (s, 1H, OH), 7.59 (d, *J* = 7.8 Hz, 2H, Ar H), 7.43 (s, 1H, Ar H), 7.29 (d, *J* = 7.8 Hz, 2H, Ar H), 6.27 (s, 1H, OH), 5.85 (s, 1H, OH), 2.57 (d, *J* = 7.2 Hz, 2H, CH_2), 1.94 (q, *J* = 13.5, 6.7 Hz, 1H, CH), 0.95 (s, 3H, CH_3), 0.94 (s, 3H, CH_3). ^{13}C NMRδ/ppm (101 MHz, d_6-DMSO): 199.68 (CO), 150.65 (Ar C), 146.85 (Ar C), 146.48 (Ar C), 134.81 (Ar C), 132.35 (Ar C), 129.28 (Ar C), 129.17 (Ar C), 127.98 (Ar C), 114.14 (Ar C), 99.38 (Ar C), 45.41 (CH_2), 30.15 (CH), 22.40 (CH_3). MS (M^-):365.26. IR cm^{-1}: 3341 br m, 1607 m, 1287 s, 1178 s, 882 s, 781 s.

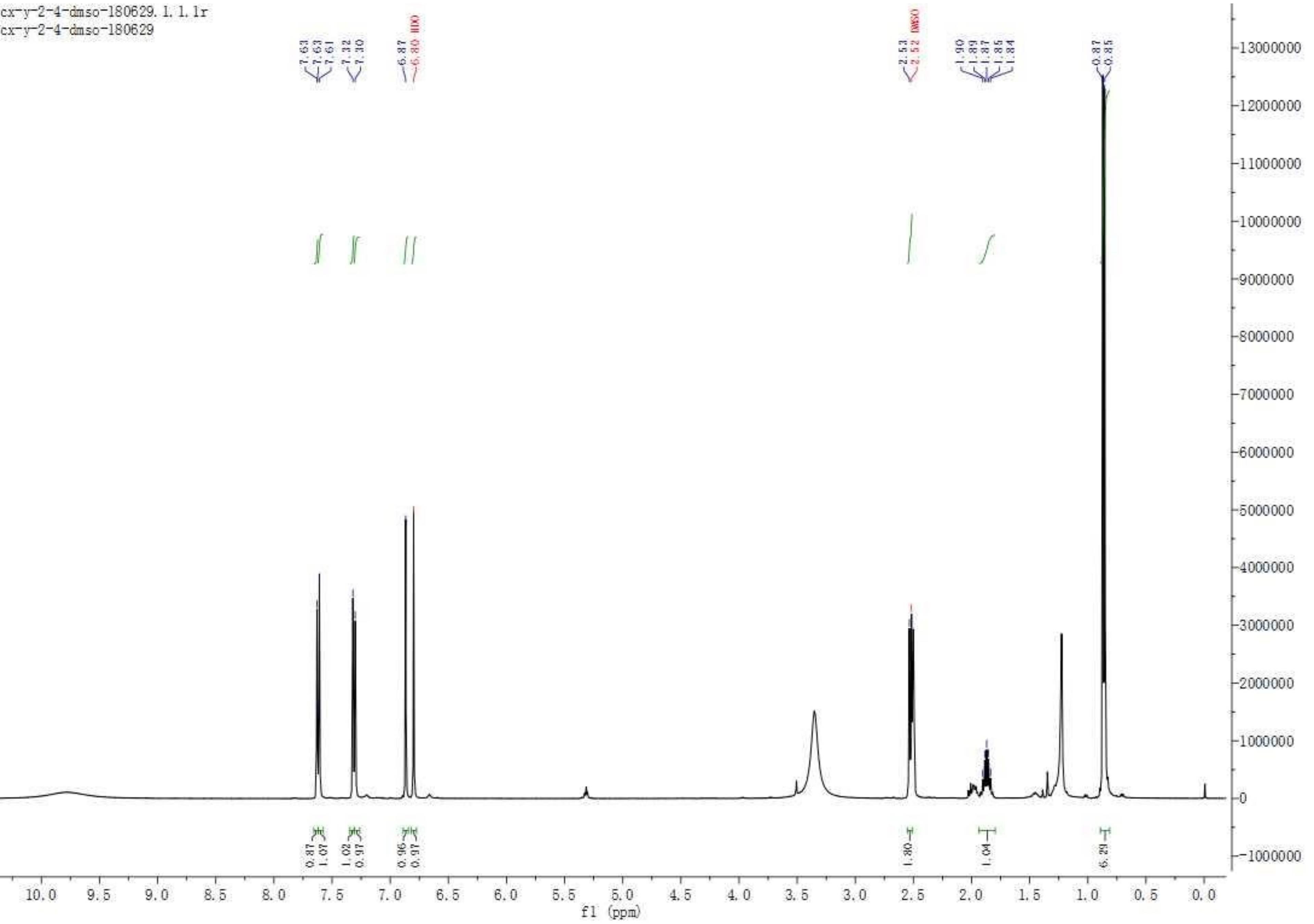

Figure 25S ^{1}H-NMR of compound 5g

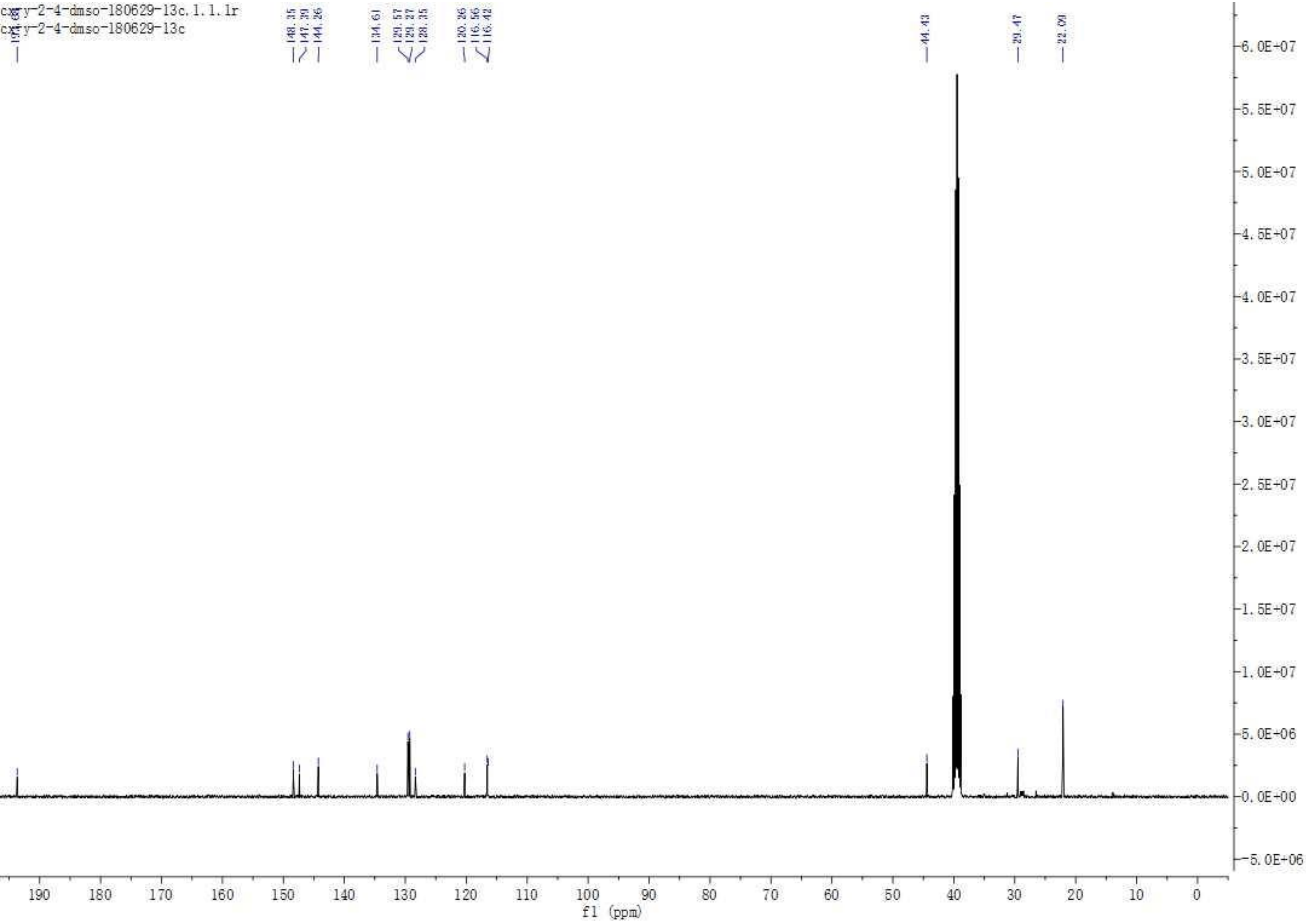

Figure 26S ^{13}C-NMR of compound 5g

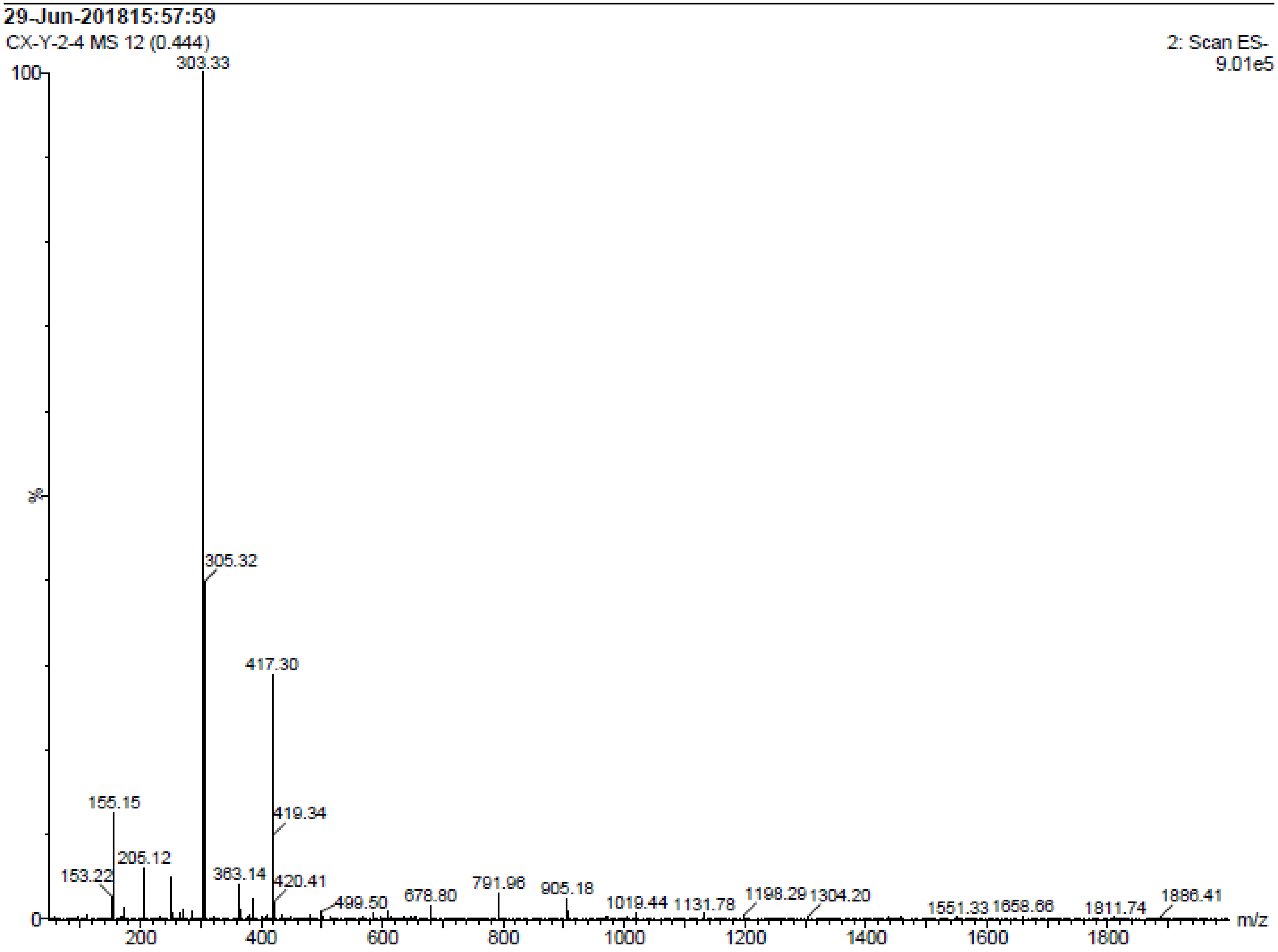

Figure 27S ESI-MS of compound 5g

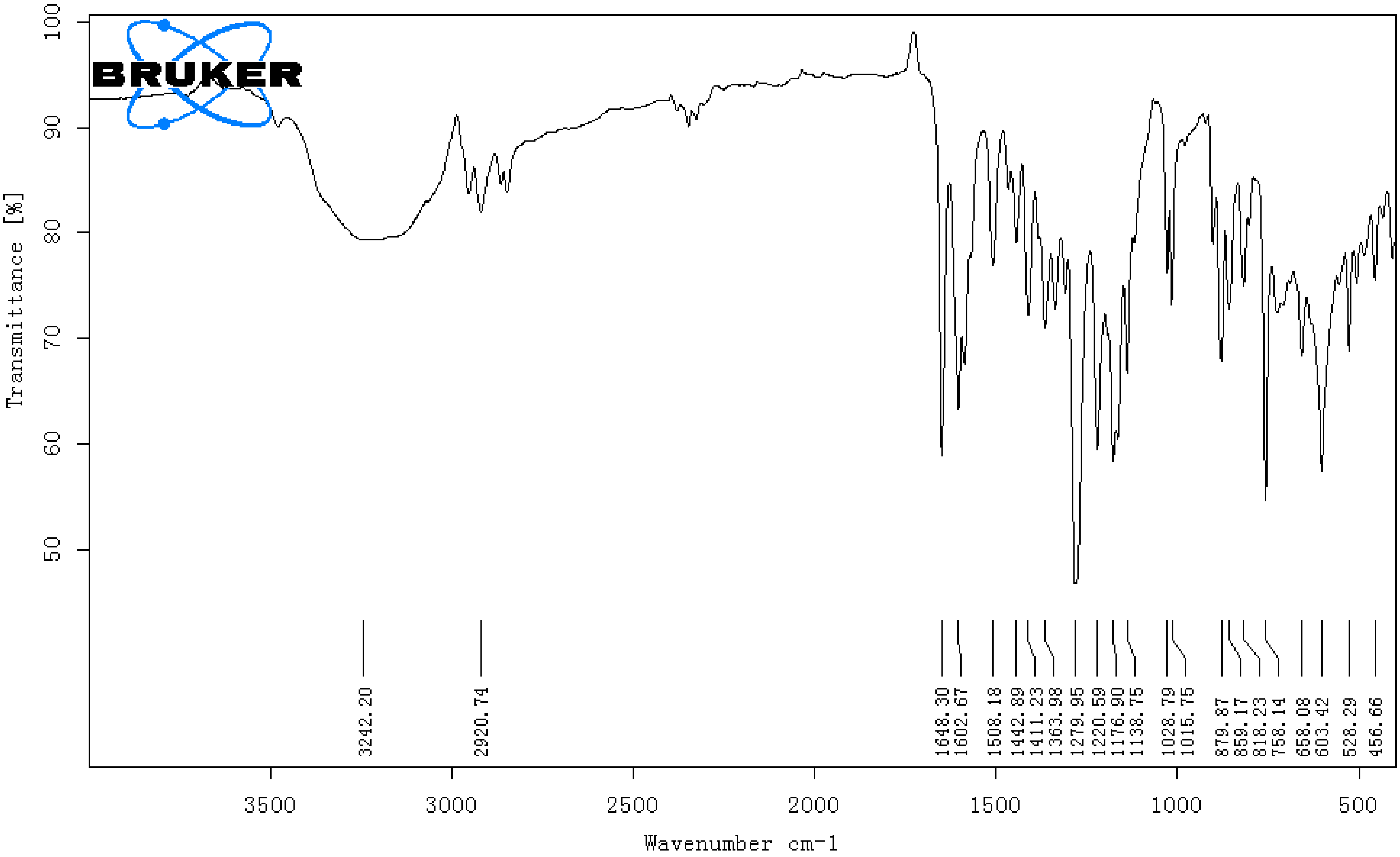

Figure 28S IR of compound 5g

- (2-chloro-4, 5-dihydroxy-phenyl)-(4-isobutyl-phenyl)-methanone (5g): Brown solid. Yield: 50.3%. M.P.: 105.0-105.7 ℃. ^{1}H NMRδ/ppm (400 MHz, d_6-DMSO):7.63 (d, *J* = 1.7 Hz, 1H, Ar H), 7.61 (s, 1H, Ar H), 7.32 (s, 1H, Ar H), 7.30 (s, 1H, Ar H), 6.87 (s, 1H, Ar H), 6.80 (s, 1H, Ar H), 2.53 (s, 2H, CH_2), 1.87 (h, *J* = 6.9 Hz, 1H, CH), 0.86 (d, *J* = 6.6 Hz, 6H, 2 × CH_3). ^{13}C NMRδ/ppm (101 MHz, d_6-DMSO):193.68 (CO), 148.35 (Ar C), 147.39 (Ar C), 144.26 (Ar C), 134.61 (Ar C), 129.57 (Ar C), 129.27 (Ar C), 128.35 (Ar C), 120.26 (Ar C), 116.56 (Ar C), 116.42 (Ar C), 44.43 (CH_2), 29.47 (CH), 22.09 (CH_3). MS (M^-):303.33. IR cm^{-1}: 3242 br m, 1648 s, 1279 s, 758 s.

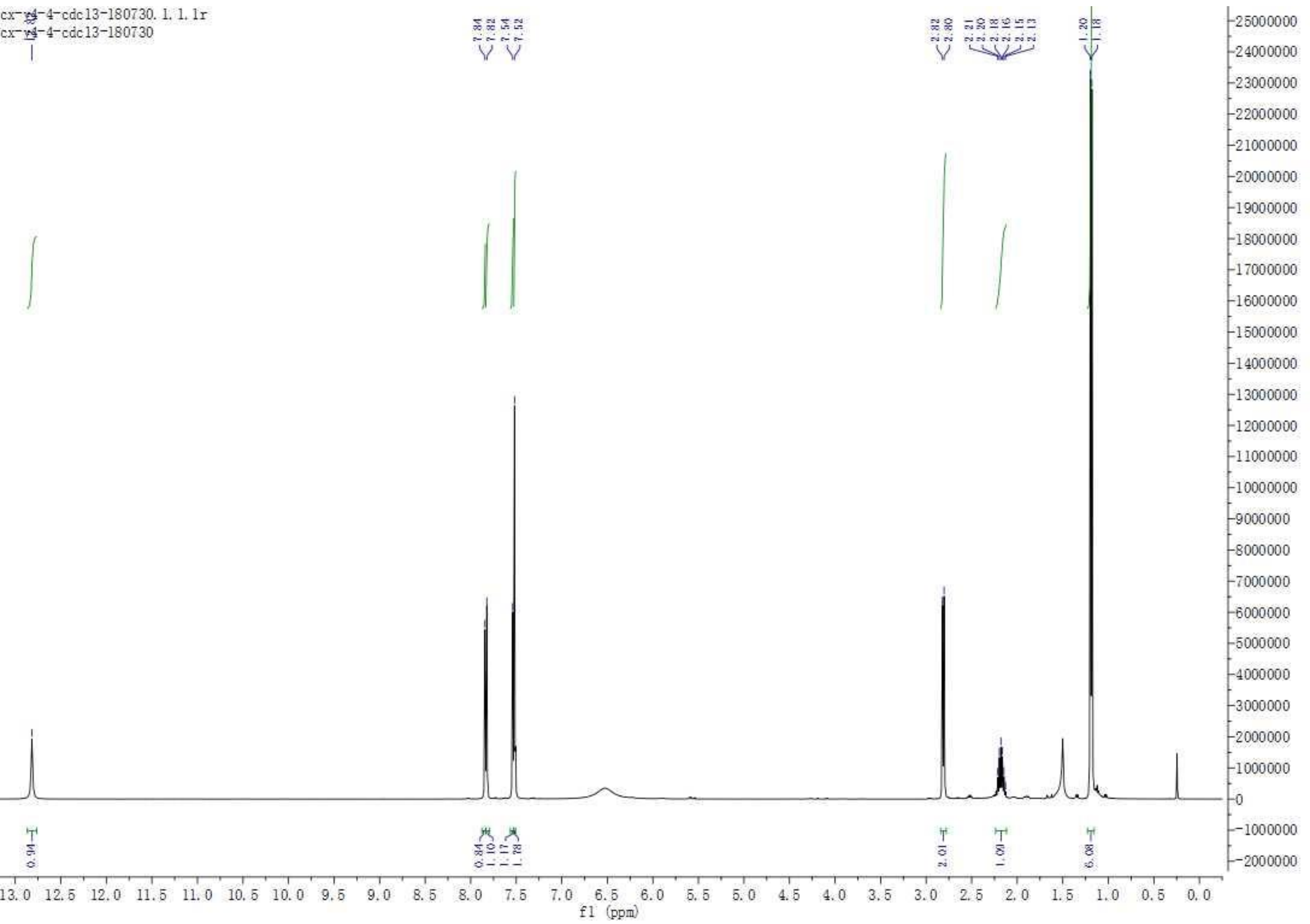

Figure 29S ¹H-NMR of compound 5h

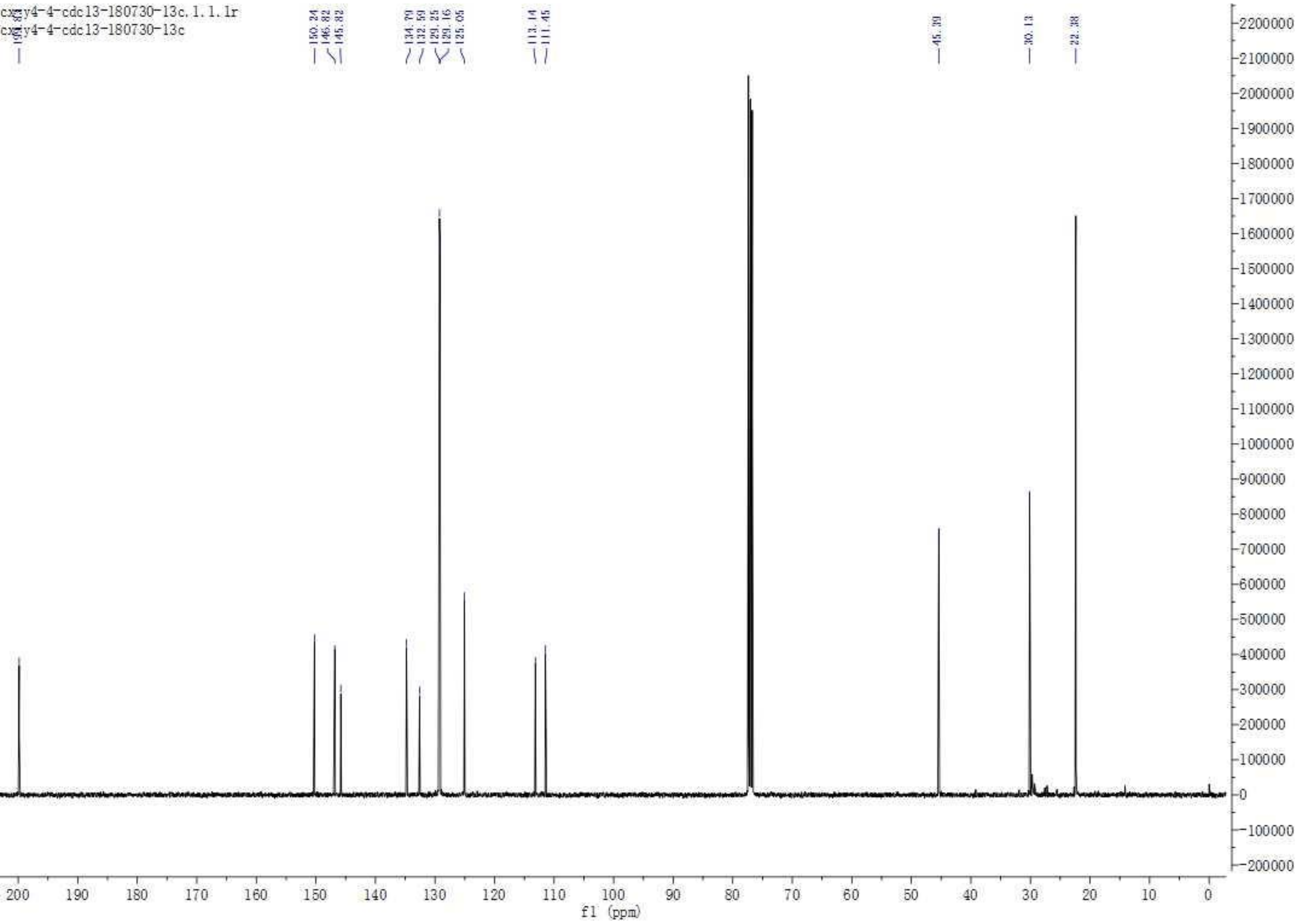

Figure 30S ^{13}C-NMR of compound 5h

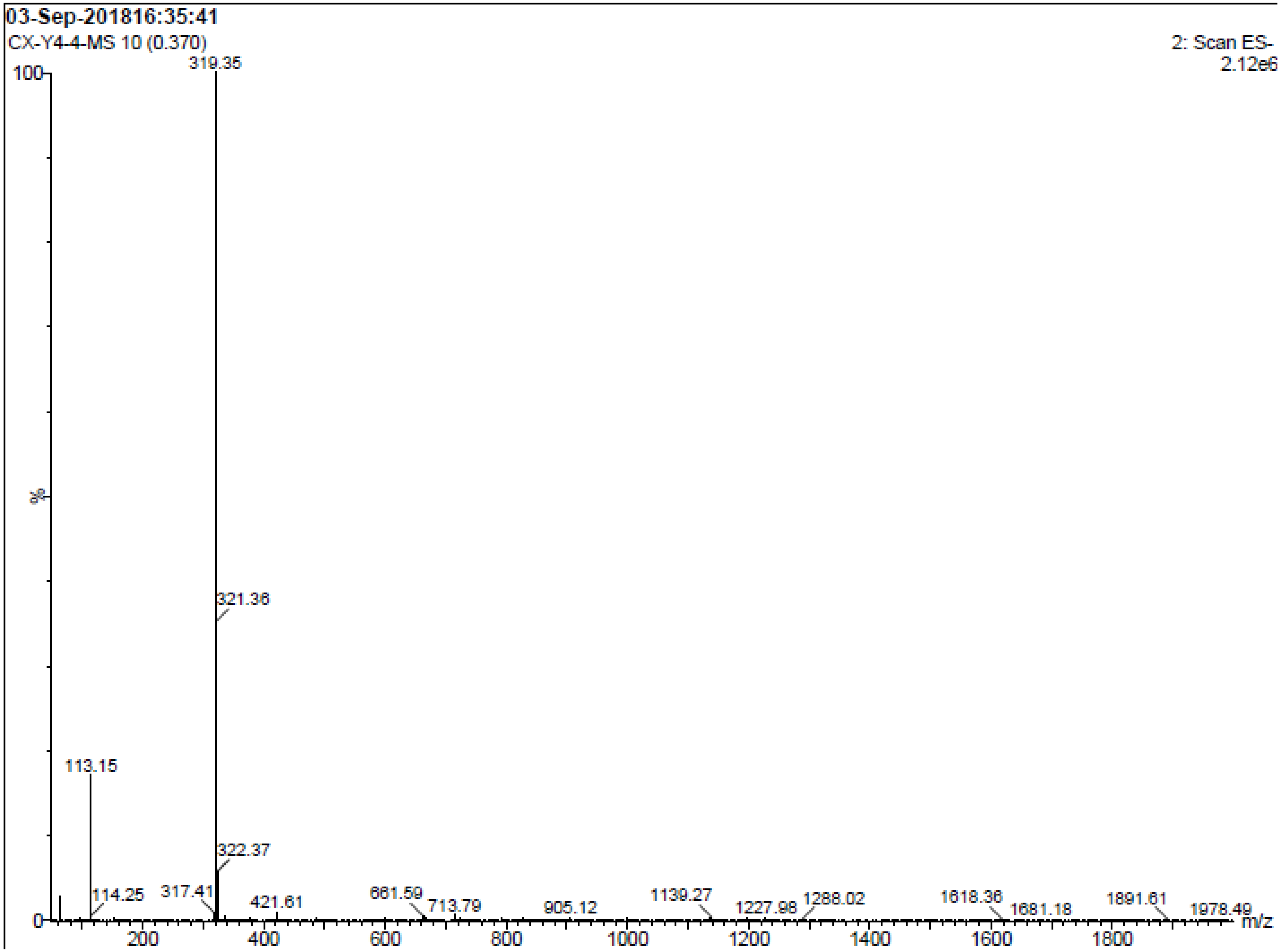

Figure 31S ESI-MS of compound 5h

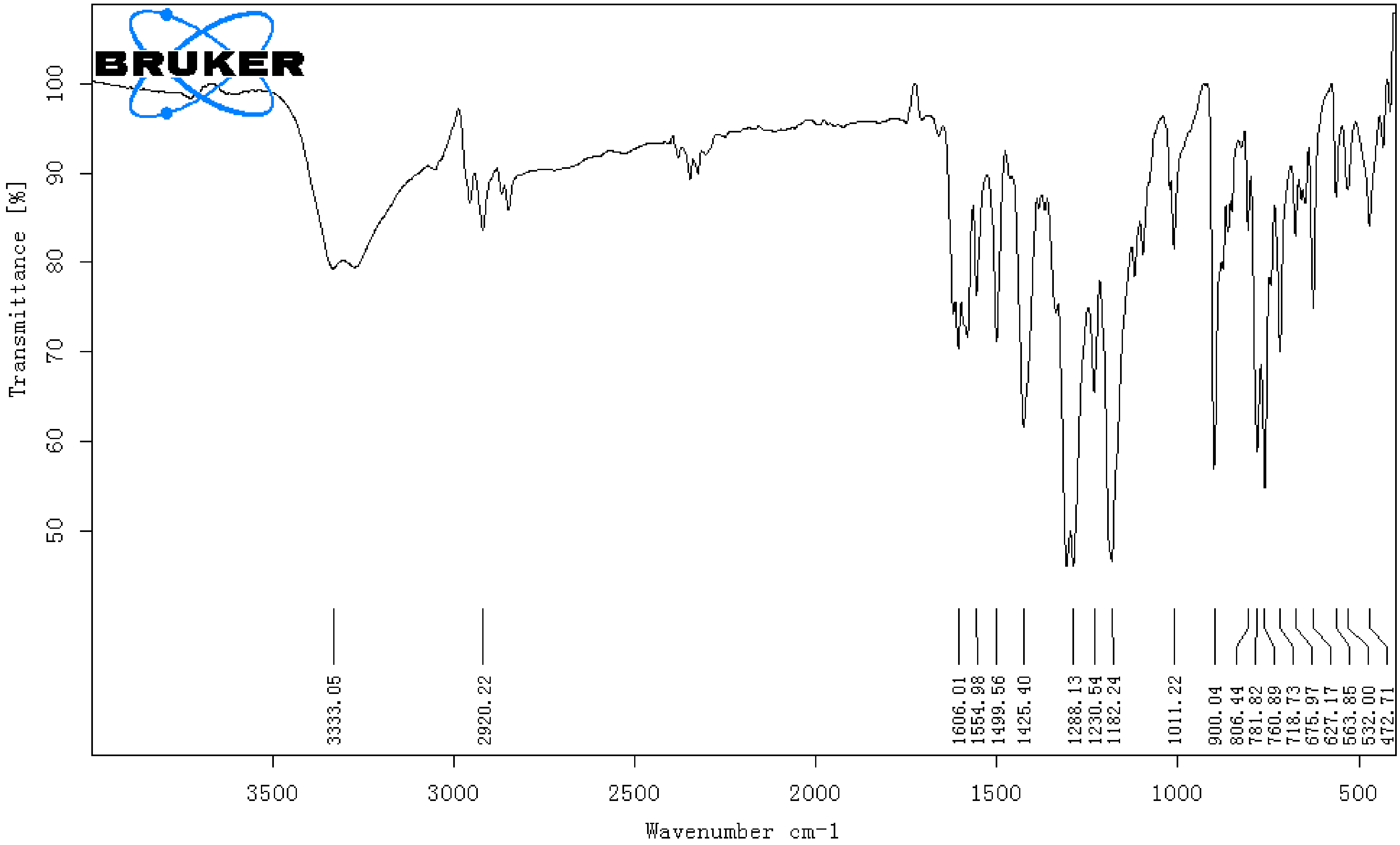

Figure 32S IR of compound 5h

- (6-chloro-2, 3, 4-trihydroxy-phenyl)-(4-isobutyl-phenyl)-methanone (5h): Yellow solid. Yield: 42.8%. M.P.: 138.5-139.2 ℃. ^{1}H NMRδ/ppm (400 MHz, d_6-DMSO): 12.82 (s, 1H, OH), 7.84 (d, *J* = 1.7 Hz, 1H, Ar H), 7.83 (d, *J* =2.0 Hz, 1H, Ar H), 7.54 (s, 1H, Ar H), 7.52 (s, 2H, Ar H), 2.81 (d, *J* = 7.1 Hz, 2H, CH_2), 2.18 (h, *J* = 6.7 Hz, 1H, CH), 1.19 (d, *J* = 6.7 Hz, 6H, 2 × CH_3). ^{13}C NMRδ/ppm (101 MHz, d_6-DMSO): 199.83 (CO), 150.24 (Ar C), 146.82 (Ar C), 145.82 (Ar C), 134.79 (Ar C), 132.59 (Ar C), 129.25 (Ar C), 129.16 (Ar C), 125.05 (Ar C), 113.14 (Ar C), 111.45 (Ar C), 45.39 (CH_2), 30.13 (CH), 22.38 (CH_3). MS (M^-):319.35. IR cm^{-1}: 3333 br m, 1606 m, 1288 s, 1182 s, 900m, 760 m.

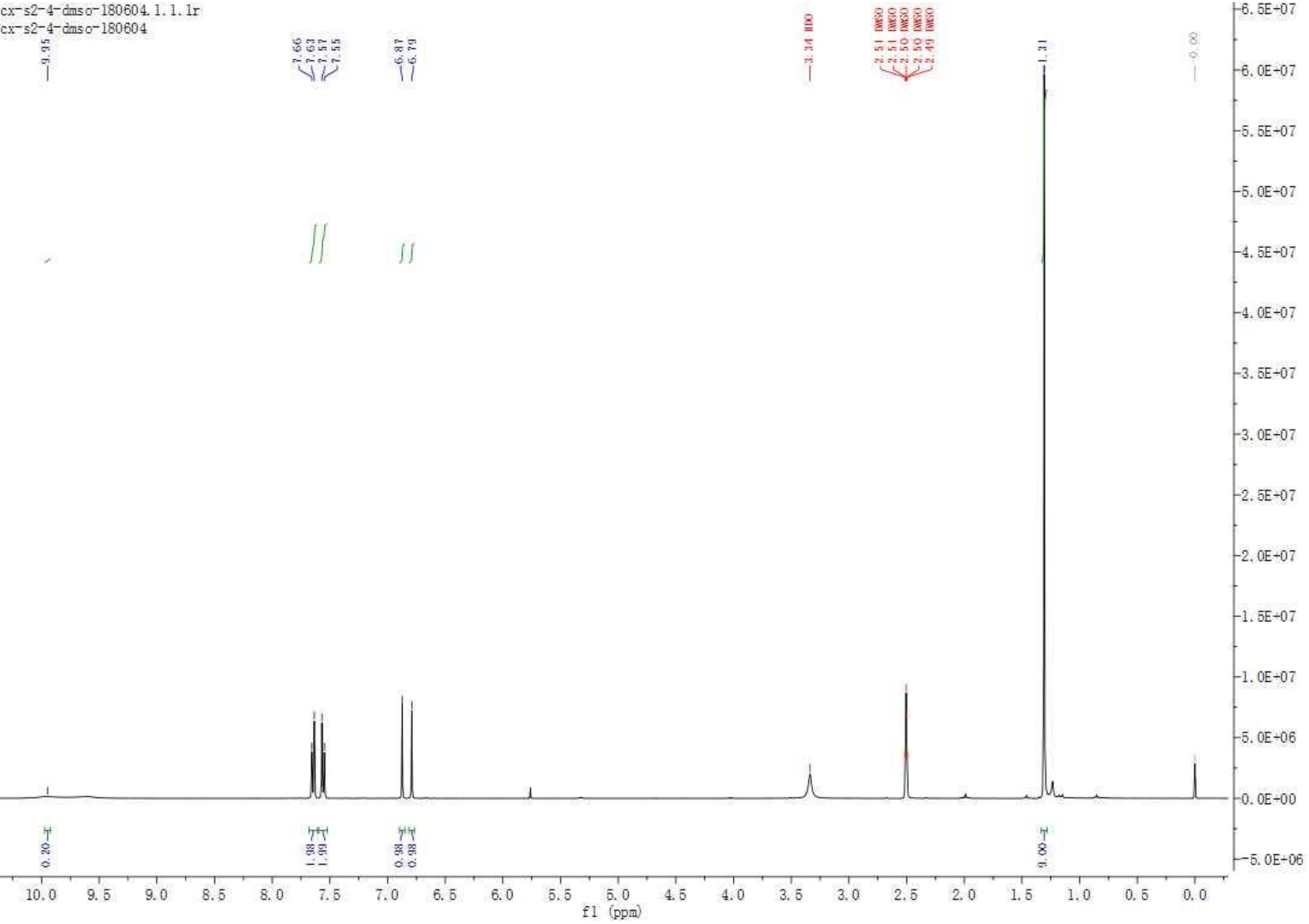

Figure 33S ^{1}H-NMR of compound 5i

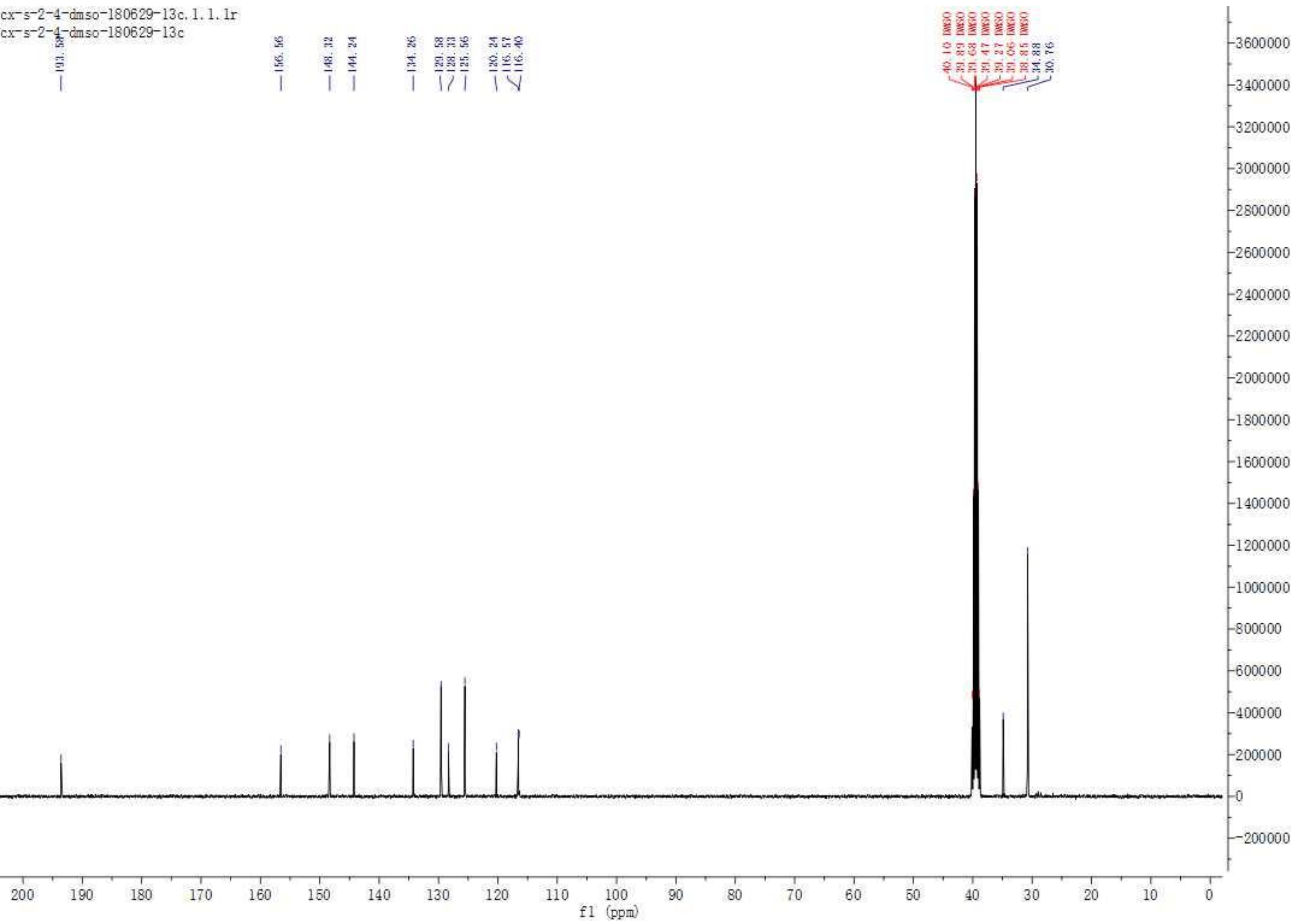

Figure 34S ^{13}C-NMR of compound 5i

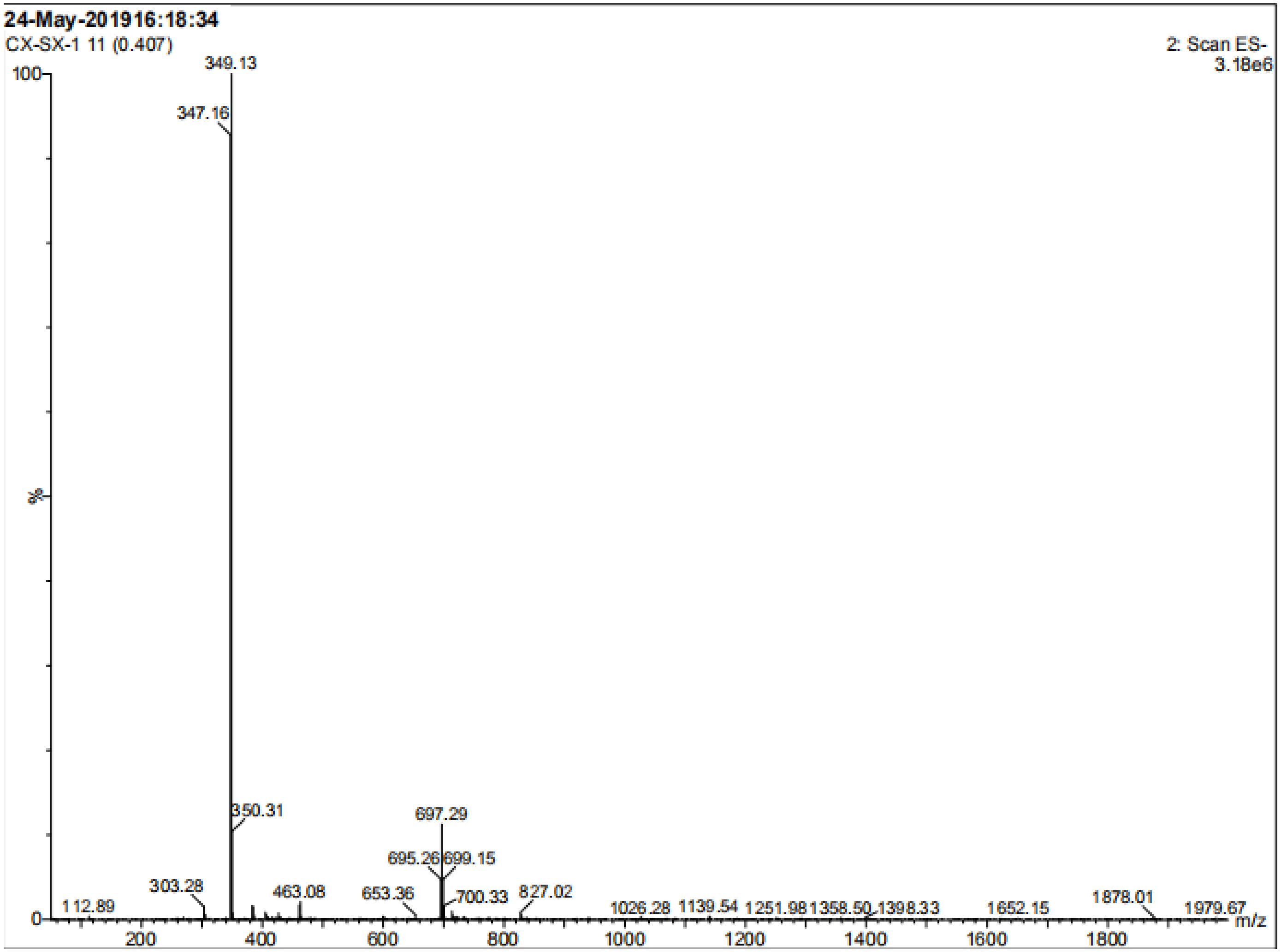

Figure 35S ESI-MS of compound 5i

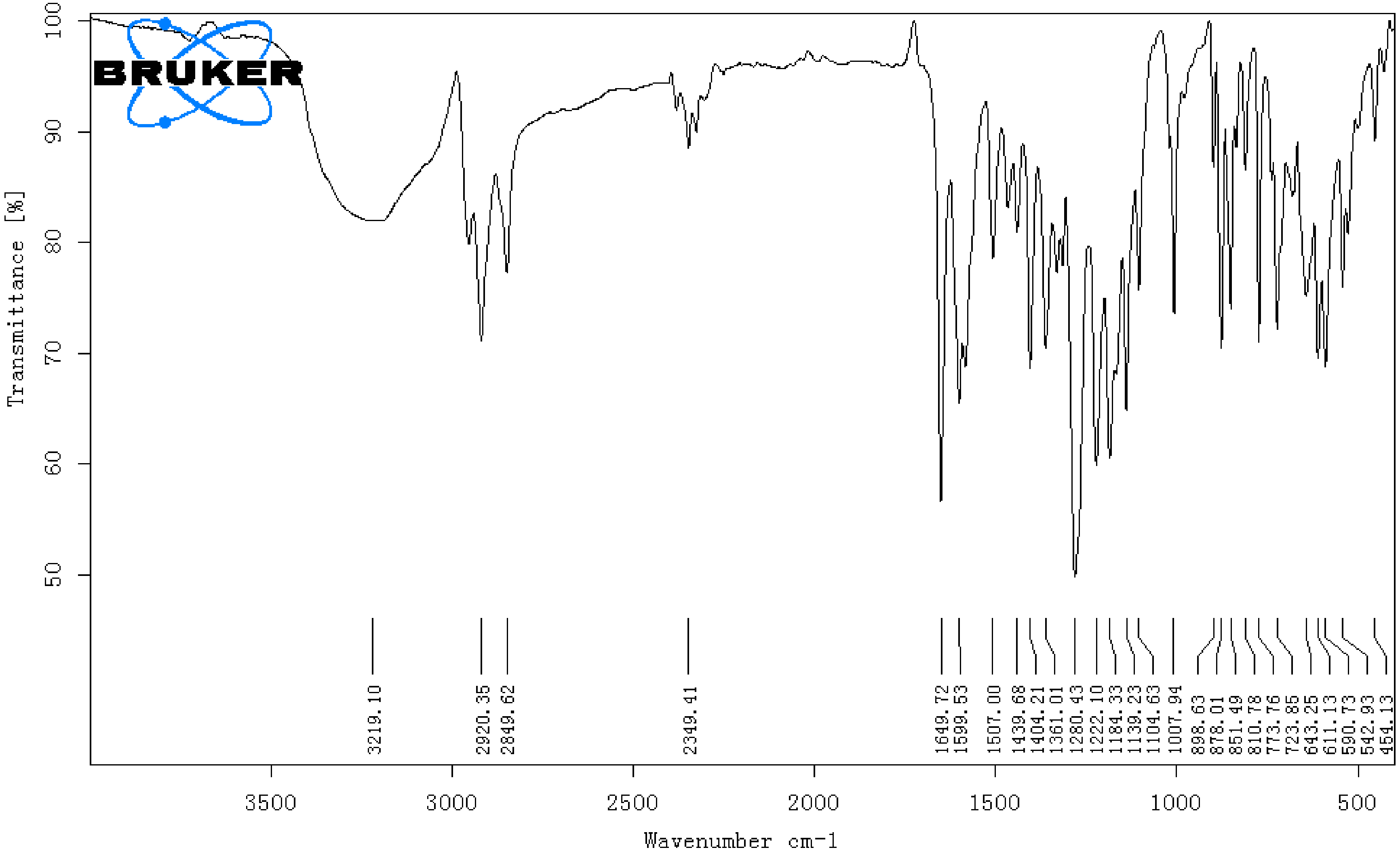

Figure 36S IR of compound 5i

- (2-bromo-4, 5-dihydroxy-phenyl)-(4-tert-butyl-phenyl)-methanone (5i): Brown solid. Yield: 42.5%. M.P.: 113.0-114.5 ℃. ^{1}H NMRδ/ppm (400 MHz, d_6-DMSO): 9.95 (s, 1H, OH), 7.65 (d, *J* = 8.6 Hz, 2H, Ar H), 7.56 (d, *J* = 8.6 Hz, 2H, Ar H), 6.87 (s, 1H, Ar H), 6.79 (s, 1H, Ar H), 1.31 (s, 9H, 3 × CH_3). ^{13}C NMRδ/ppm (101 MHz, d_6-DMSO): 193.58 (CO), 156.56 (Ar C), 148.32 (Ar C), 144.24 (Ar C), 134.26 (Ar C), 129.58 (Ar C), 128.33 (Ar C), 125.56 (Ar C), 120.24 (Ar C), 116.57 (Ar C), 116.40 (Ar C), 34.88 ($C(CH_3)_3$), 30.76 (CH_3). MS (M^-): 349.13. IR cm^{-1}: 3219 br m, 2920 s, 1649 s, 1599 s, 1280 s, 878 s.

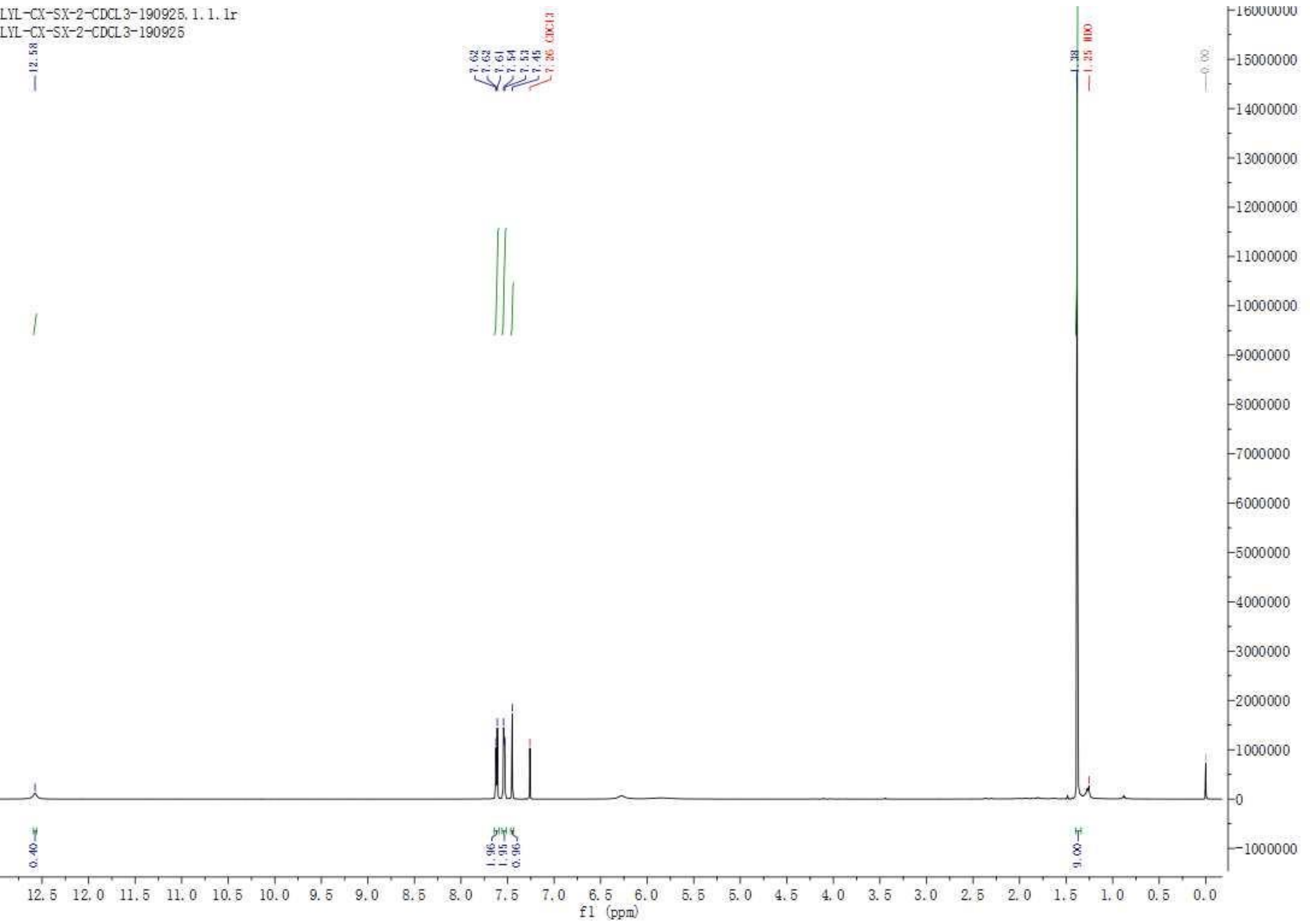

Figure 37S ^{1}H-NMR compound 5j

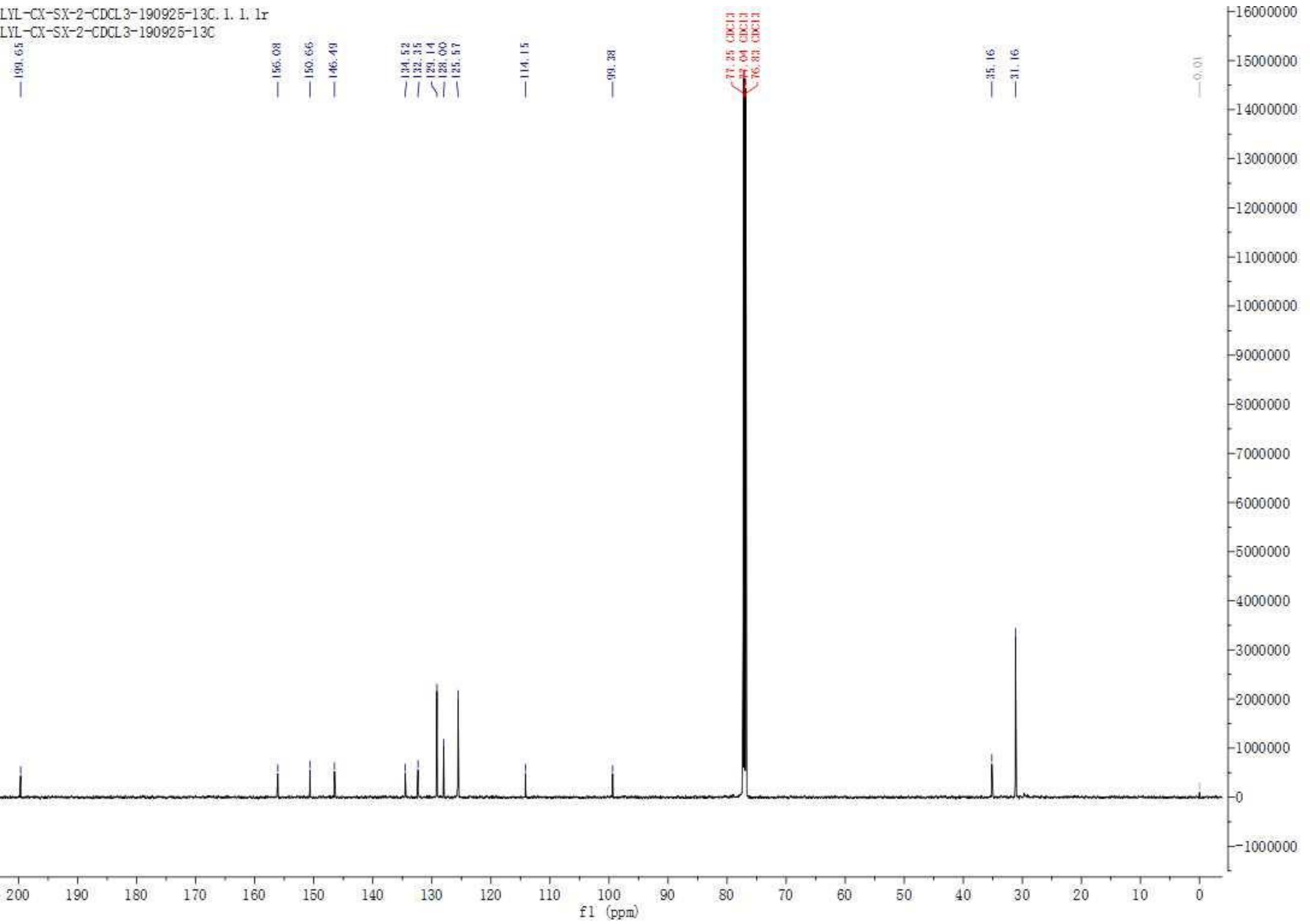

Figure 38S ^{13}C-NMR of compound 5j

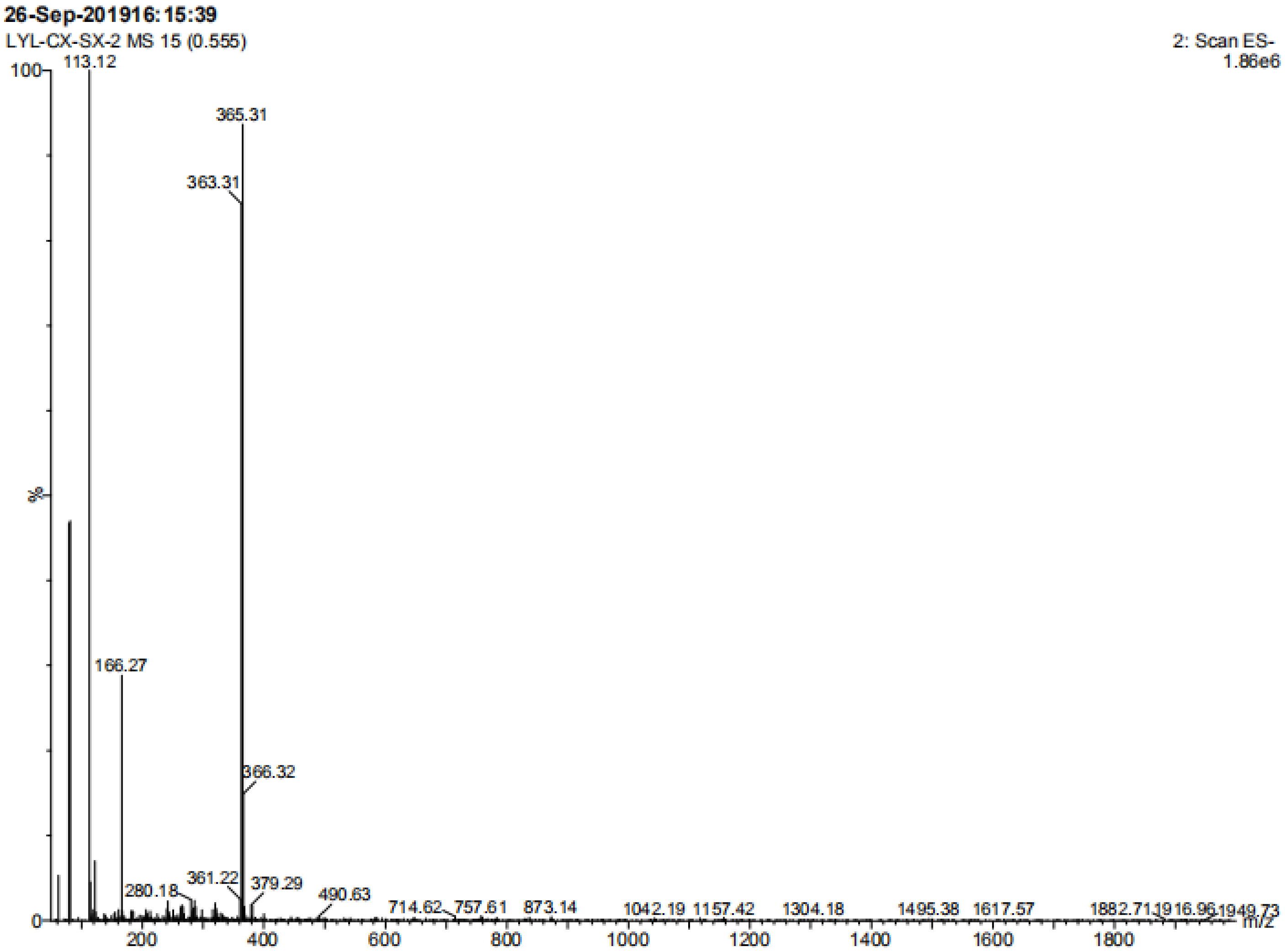

Figure 39S IR of compound 5j

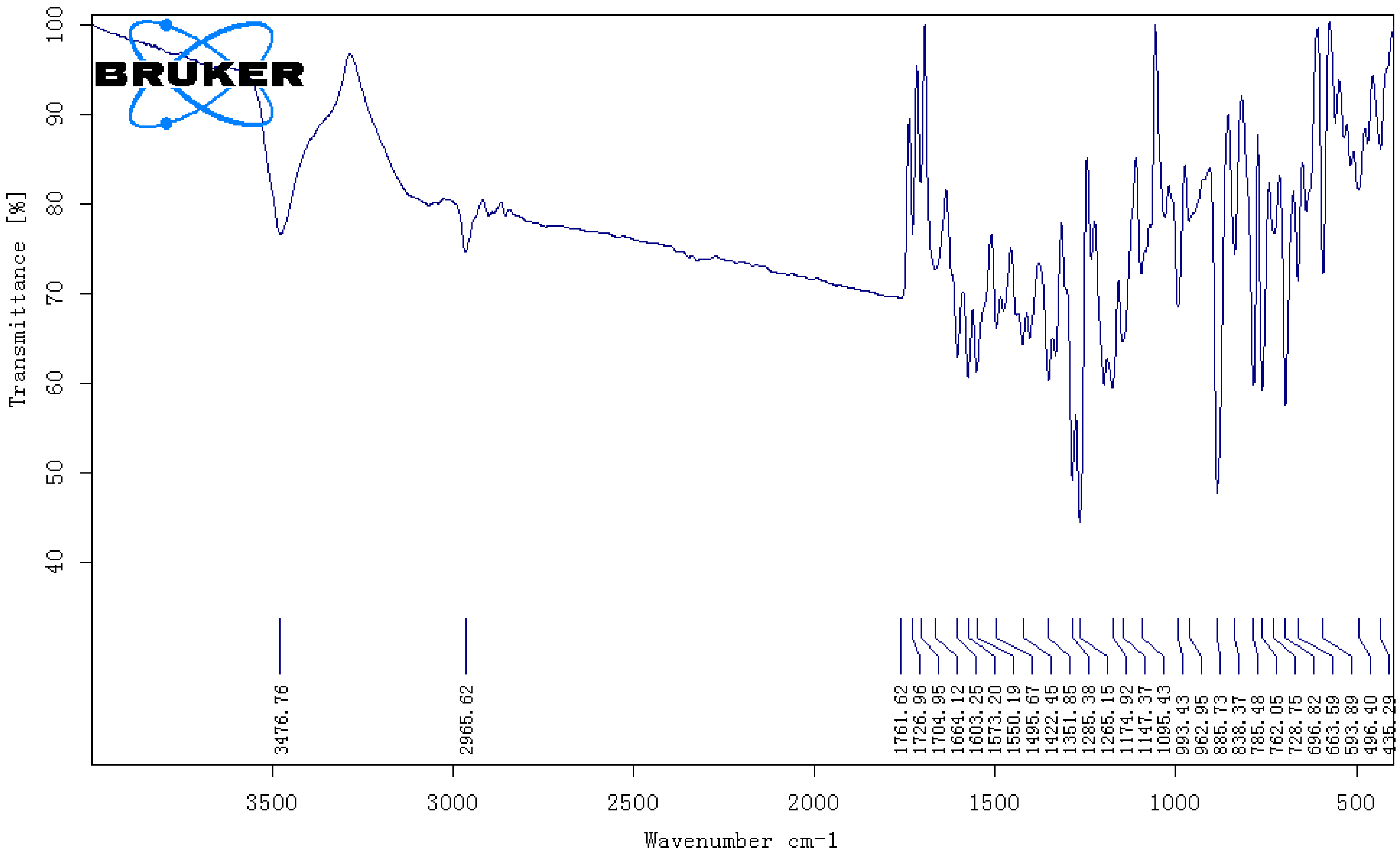

Figure 40S IR of compound 5j

- (6-bromo-2, 3, 4-trihydroxy-phenyl)-(4-tert-butyl-phenyl)-methanone (5j): Brown solid. Yield: 40.0% M.P.: 131.3-132.5 ℃. ^{1}H NMRδ/ppm (400 MHz, d_6-DMSO): 12.58 (s, 1H, OH), 7.62 (d, *J* = 8.3 Hz, 2H, Ar H), 7.54 (d, *J* = 8.2 Hz, 2H, Ar H), 7.45 (s, 1H, Ar H), 1.38 (s, 9H, 3 × CH_3) . ^{13}C NMRδ/ppm (101 MHz, d_6-DMSO): 199.65 (CO), 156.08 (Ar C), 150.66 (Ar C), 146.49 (Ar C), 134.52 (Ar C), 132.35 (Ar C), 129.14 (Ar C), 128.00 (Ar C), 125.57 (Ar C), 114.15 (Ar C), 99.38 (Ar C), 35.16 ($C(CH_3)_3$), 31.16 (CH_3). MS (M^-):365.31. IR cm^{-1}: 3476br m, 1603 s, 1265 s, 885 s.

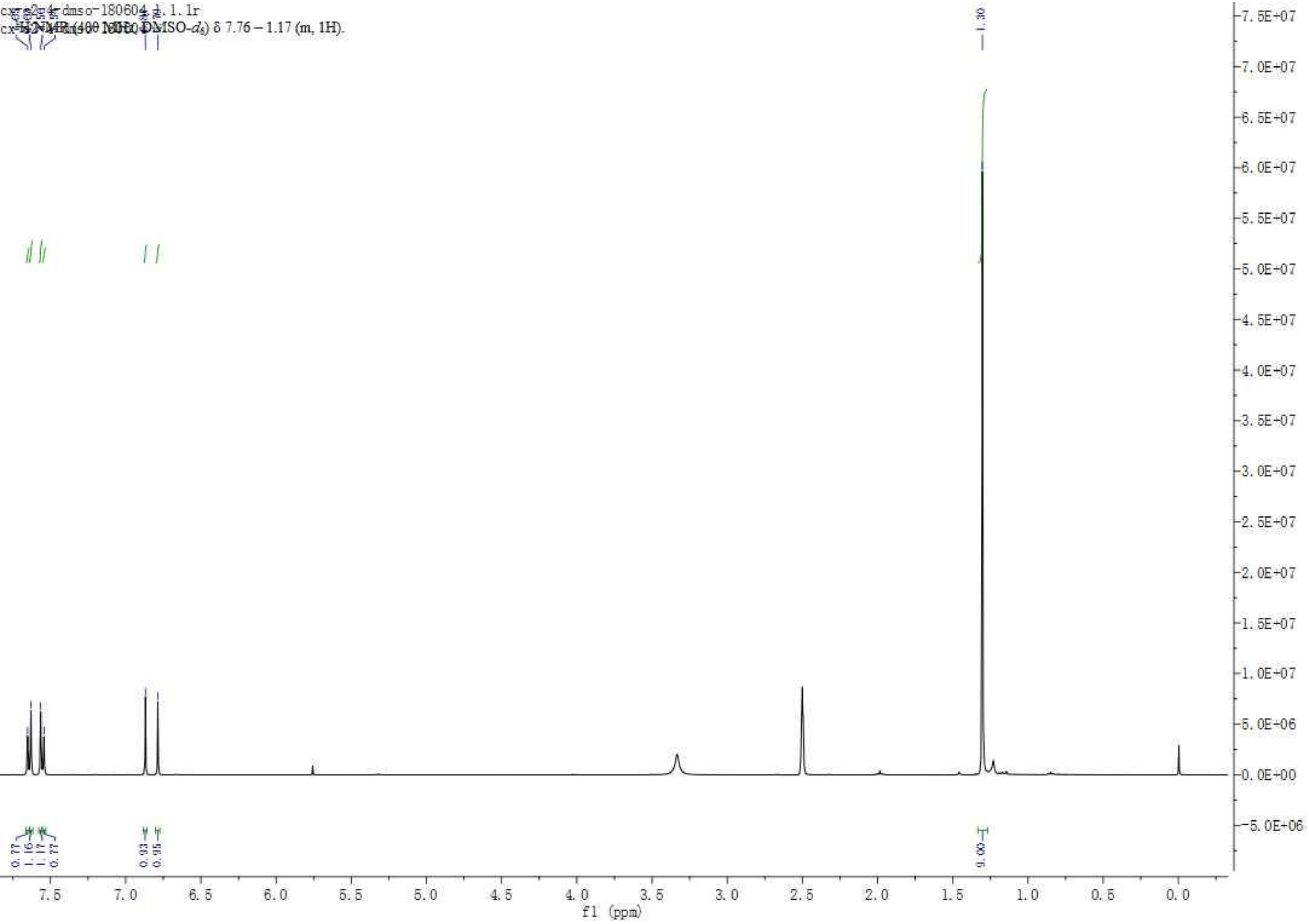

Figure 41S ^{1}H-NMR of compound 5k

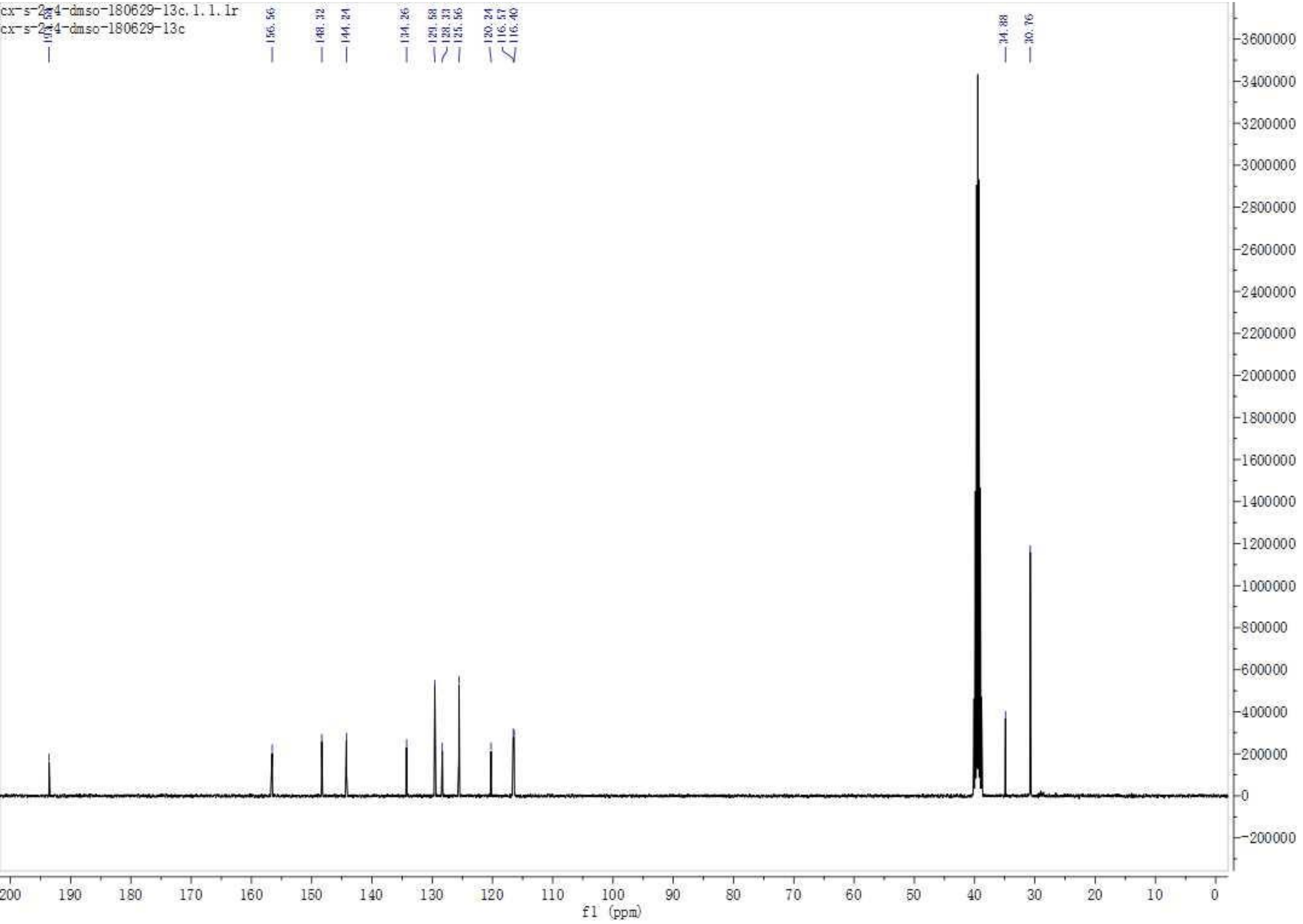

Figure 42S ^{13}C-NMR of compound 5k

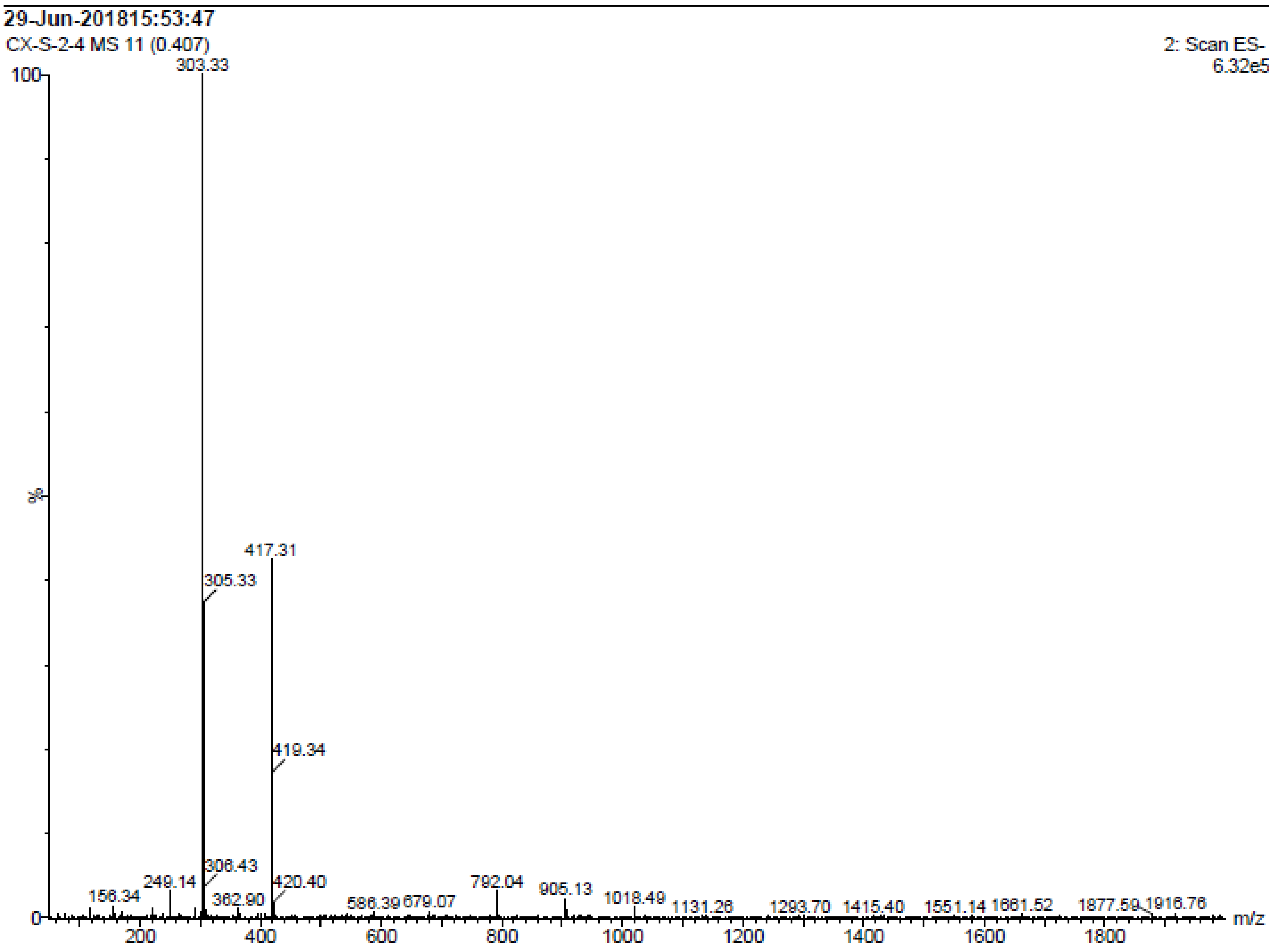

Figure 43S ESI-MS of compound 5k

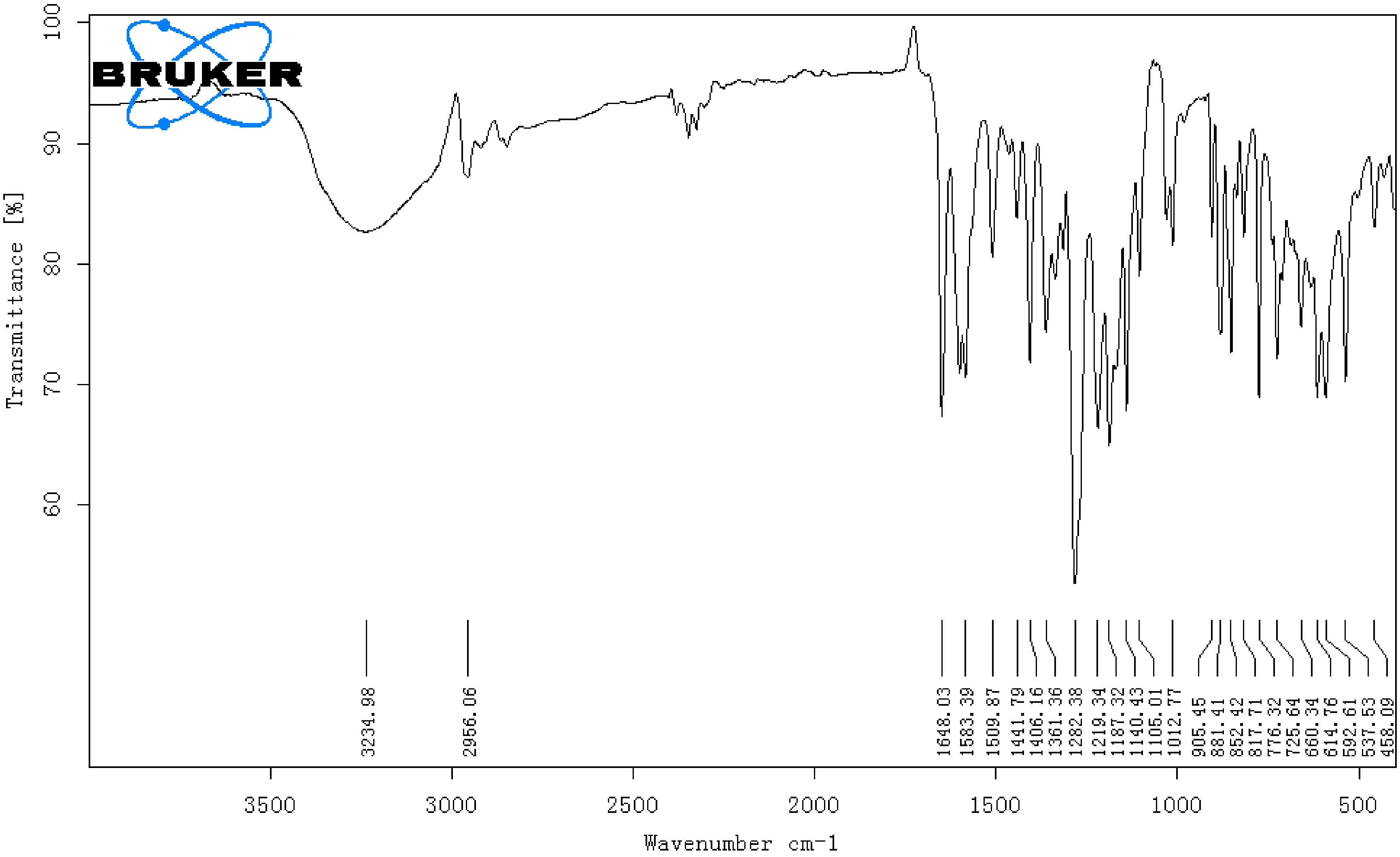

Figure 44S IR of compound 5k

- (4-tert-butyl-phenyl)-(2-chloro-4, 5-dihydroxy-phenyl)-methanone (5k): Brown solid. Yield: 45.8%. M.P.: 116.3-117.1 ℃. ^{1}H NMRδ/ppm (400 MHz, d_6-DMSO): 7.65 (s, 1H Ar H), 7.63 (s, 1H Ar H), 7.56 (s, 1H Ar H), 7.54 (s, 1H Ar H),6.87 (s, 1H Ar H), 6.79 (s, 1H Ar H), 1.30 (s, 9H, 3 × CH_3). ^{13}C NMRδ/ppm (101 MHz, d_6-DMSO): 193.58 (CO), 156.56 (Ar C), 148.32 (Ar C), 144.24 (Ar C), 134.26 (Ar C), 129.58 (Ar C), 128.33 (Ar C), 125.56 (Ar C), 120.24 (Ar C), 116.57 (Ar C), 116.40 (Ar C), 34.88 ($C(CH_3)_3$), 30.76 (CH_3). MS (M^-): 303.33. IR cm^{-1}: 3234 br m, 1648 s, 1282 s, 725 s.

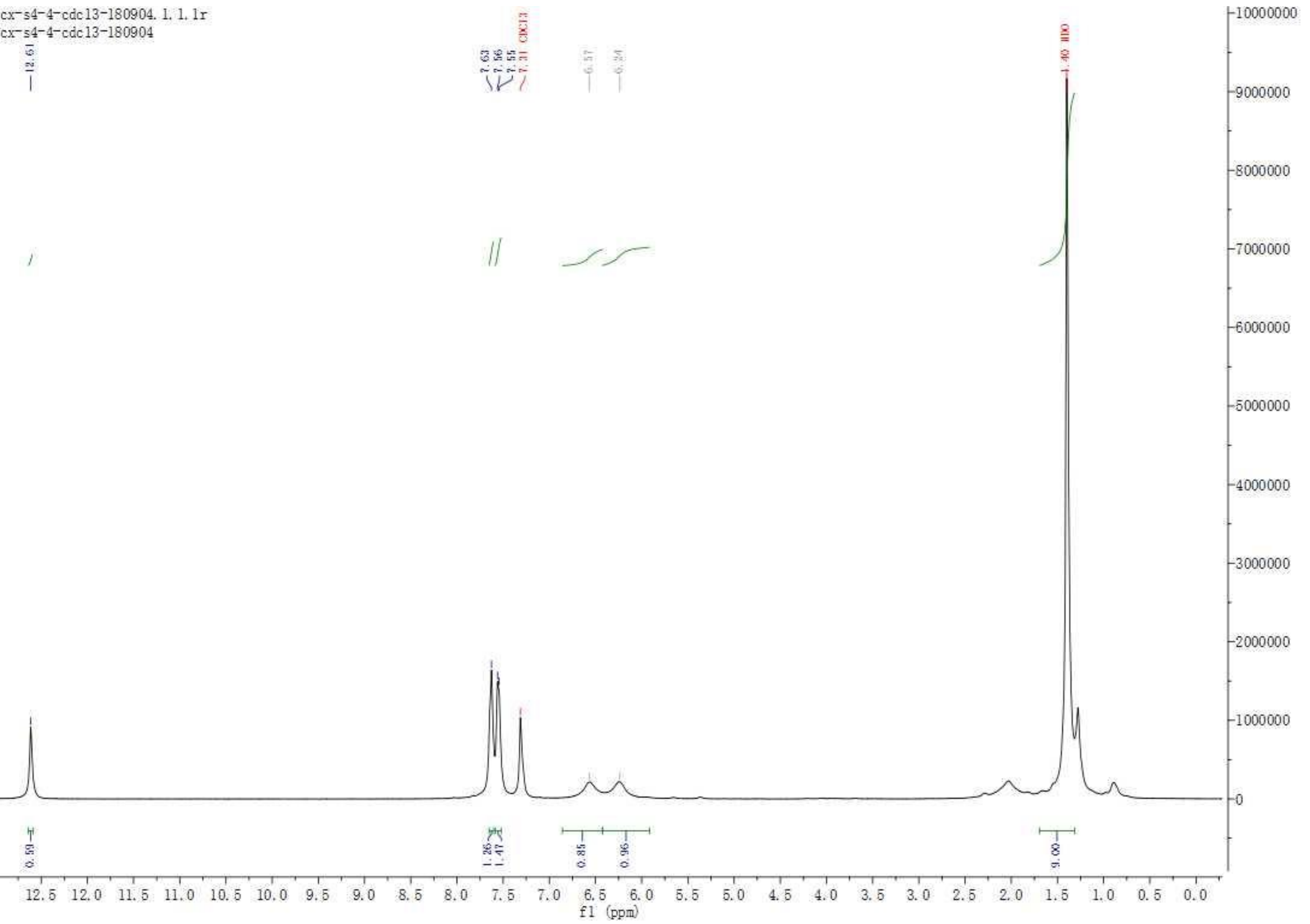

Figure 45S ¹H-NMR of compound 5l

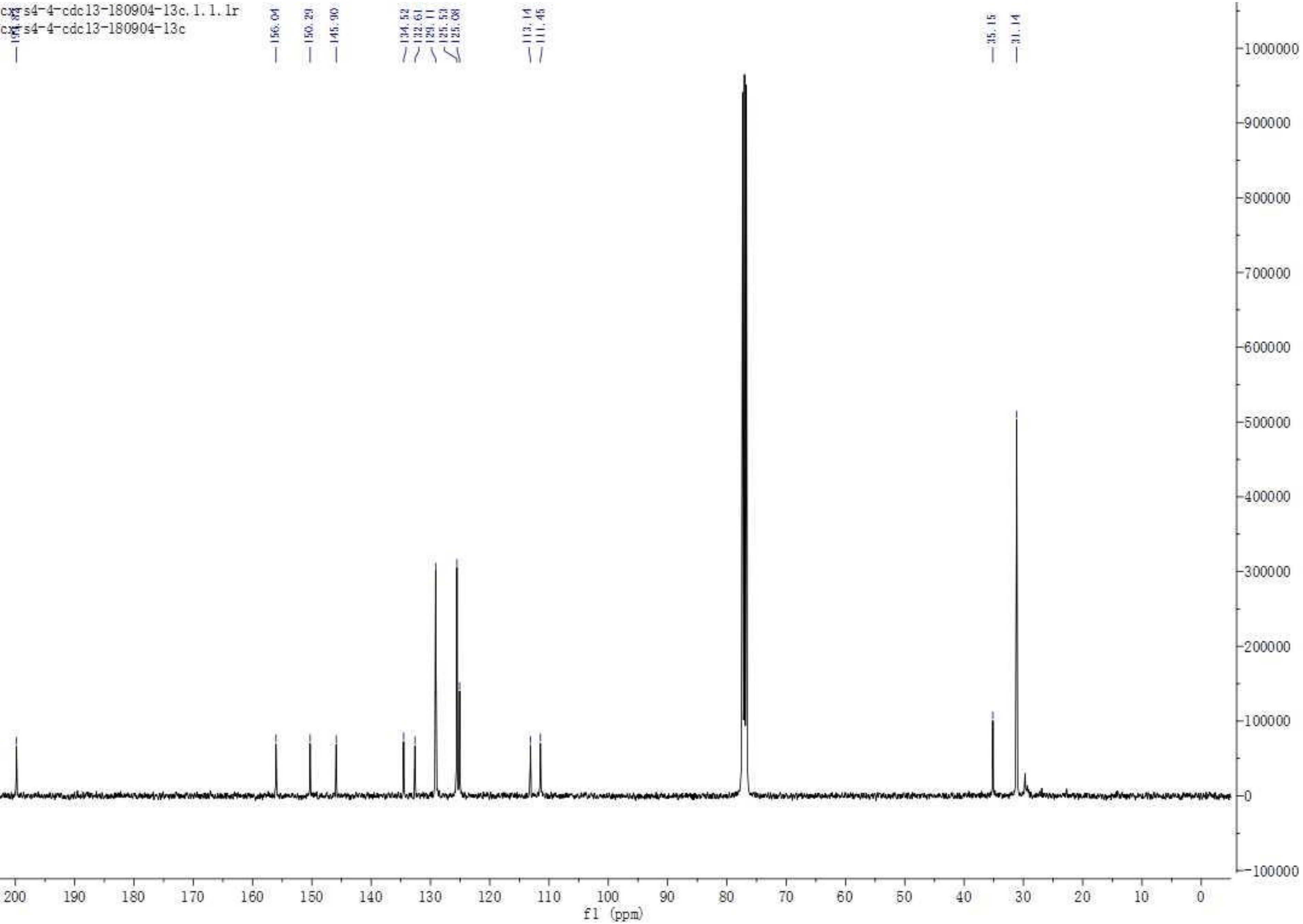

Figure 46S ^{13}C-NMR of compound 5l

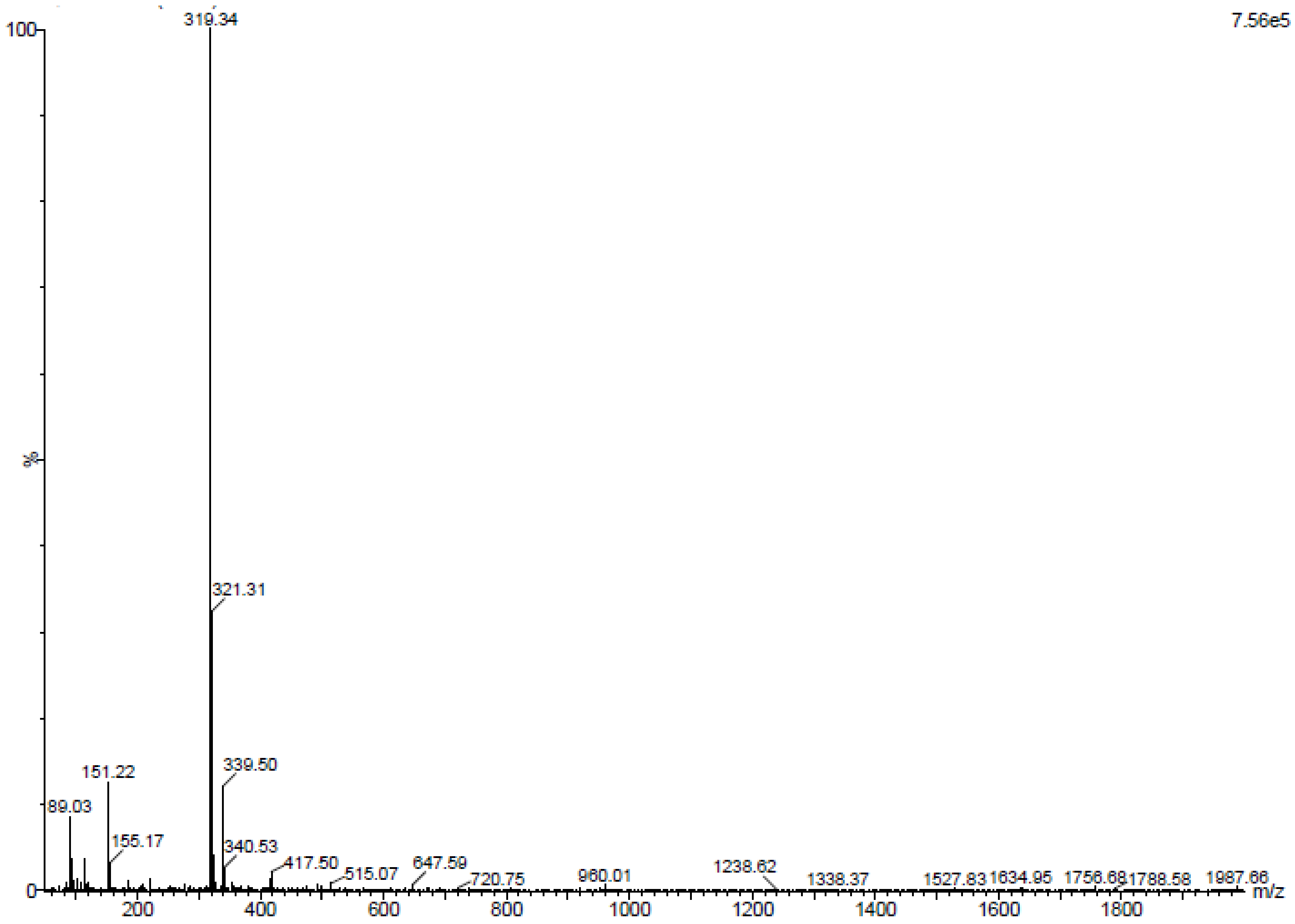

Figure 47S ESI-MS of compound 5l

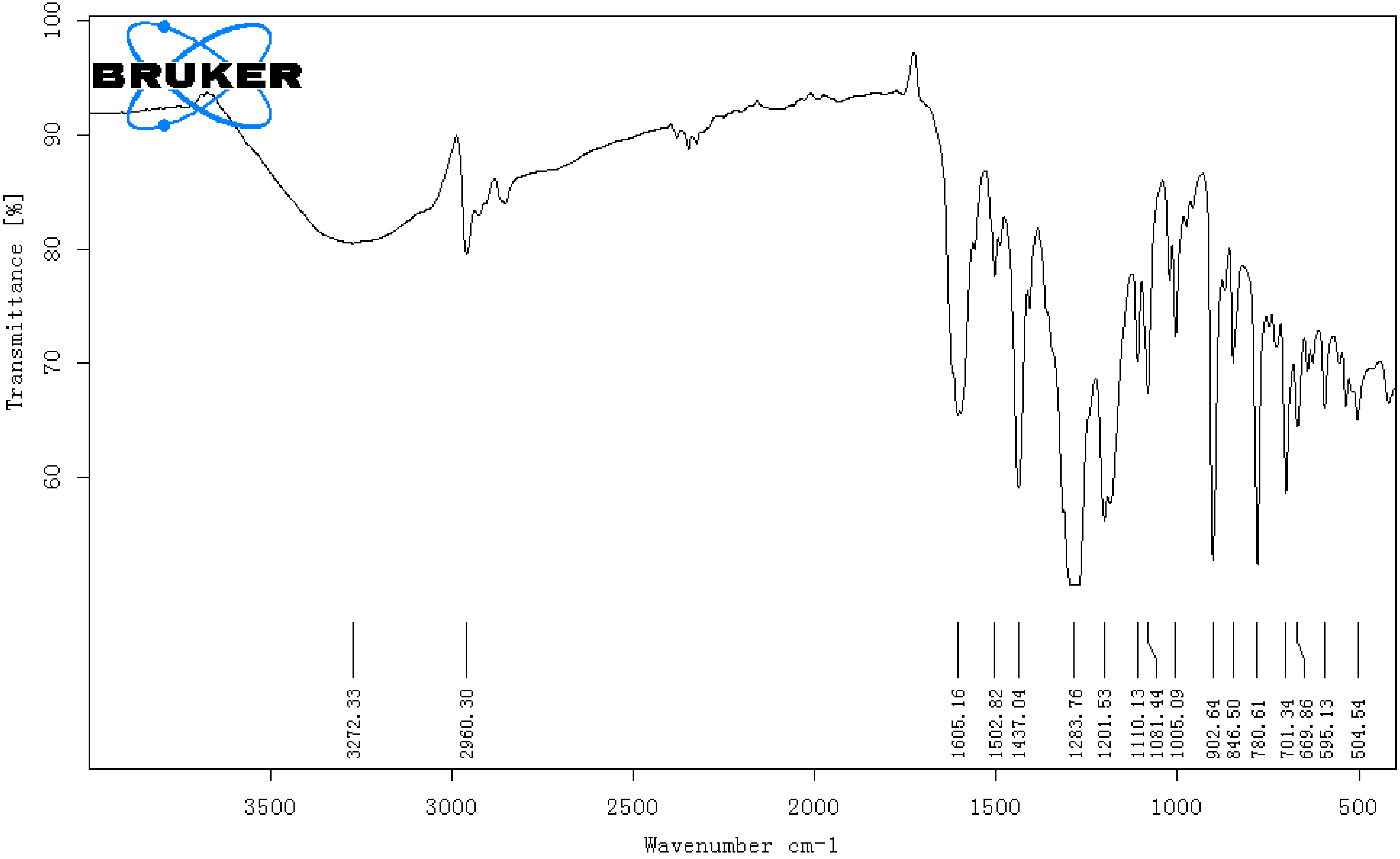

Figure 48S IR of compound 5l

- (4-tert-butyl-phenyl)-(6-chloro-2, 3, 4-trihydroxy-phenyl)-methanone (5l): Yellow solid. Yield: 39.2%. M.P.: 72.8-73.6 ℃. ^{1}H NMRδ/ppm (400 MHz, d_6-DMSO): 12.61 (s, 1H, OH), 7.59–7.52 (d, 3H, Ar H), 6.57 (s, 1H, Ar H), 6.24 (s, 1H, Ar H), 1.40 (s, 9H, 3 × CH_3). ^{13}C NMRδ/ppm (101 MHz, d_6-DMSO): 199.82 (CO), 156.04 (Ar C), 150.29 (Ar C), 145.90 (Ar C), 134.52 (Ar C), 132.61 (Ar C), 129.11 (Ar C), 125.53 (Ar C), 125.08 (Ar C), 113.14 (Ar C), 111.45 (Ar C), 35.15 ($C(CH_3)_3$), 31.14 (CH_3). MS (M^-):319.34. IR cm^{-1}: 3272 br m, 1605 s, 1283 s, 902 s, 780 s.
