# Supplementary material for: Highly Accessible Computational Prediction and In Vivo/In Vitro Experimental Validation: Novel Synthetic Phenyl Ketone Derivatives as Promising Agents against NAFLD via Modulating Oxidoreductase Activity
Source: Oxid Med Cell Longev. 2023 Jan 9;2023:3782230. doi: 10.1155/2023/3782230 (PMC9844233; doi:10.1155/2023/3782230)
Supplement: Supplementary 2 — Supplementary Material-predicted targets: the detailed information on predicted targets of twelve phenyl ketone compounds. [file 3782230.f2.pdf]

| | | | | | | | | |
|---|---|---|---|---|---|---|---|---|
| 5c | 5d | 5a | 5b | 5k | 5l | 5i | 5j | 5g |
| MAPK8 | APOA2 | MAPK8 | APOA2 | MMP3 | MAPK8 | MAPK8 | MAPK10 | MAPK8 |
| APOA2 | STS | APOA2 | STS | MAPK8 | MAPK10 | MAPK10 | MAPK8 | MMP3 |
| MMP3 | CASP7 | MAPK10 | CASP7 | MAPK10 | KIF11 | MMP3 | KIF11 | MAPK10 |
| MAPK10 | MAPK8 | MMP3 | ALB | CCNA2 | CCNA2 | GSTP1 | CCNA2 | GSTP1 |
| CASP7 | MAPK10 | STS | MAPK8 | GSTP1 | CFB | CCNA2 | CFB | MAPK1 |
| STS | LCN2 | CASP7 | LCN2 | MAOB | MMP3 | MAOB | MMP3 | CFB |
| ALB | CCNA2 | ALB | MAPK10 | CFB | MAOB | CFB | MAOB | NR1I3 |
| GSTP1 | PIM1 | GSTP1 | AKR1B1 | MAPK1 | PPARG | F2 | PPARG | BMP2 |
| CFB | AKR1B1 | PIM1 | CCNA2 | F2 | GSTP1 | MAPK1 | F2 | KIF11 |
| PIM1 | MAOB | CCNA2 | PIM1 | PDPK1 | F2 | PIM1 | THRB | PIM1 |
| MAOB | CFB | MAOB | MAOB | PIM1 | PIM1 | MAPK14 | GSTP1 | MAOB |
| KIF11 | MMP3 | NR1I3 | MMP3 | AKR1B1 | MAPK1 | PPARG | PIM1 | STS |
| NR1I3 | NR1I3 | KIF11 | CFB | MAPK14 | AKR1B1 | ESR1 | MAPK1 | CASP7 |
| MAPKAPK2 | CA2 | CFB | KIF11 | ESR1 | MMP8 | PDPK1 | AKR1B1 | F2 |
| MAPK1 | PGR | MAPKAPK2 | GSTP1 | PTPN1 | CA2 | ALB | MMP8 | APOA2 |
| ADAM17 | ADAM17 | ADAM17 | NR1I3 | TGFBR1 | ESR1 | NR3C2 | CA2 | PTPN1 |
| F2 | MAPKAPK2 | MAPK1 | PGR | MMP8 | CES1 | ADAM17 | PTPN1 | CCNA2 |
| CA2 | PPARG | F2 | ADAM17 | GC | PTPN1 | GC | ESR1 | PDPK1 |
| PGR | F2 | CA2 | MAPKAPK2 | NR3C2 | RORA | AKR1B1 | RORA | AKR1C2 |
| AKR1B1 | PTPN1 | PDPK1 | CA2 | KDR | GC | RORA | ALB | CA2 |
| PDPK1 | GSTP1 | TTR | PPARG | PPARG | MAPK14 | APOA2 | BACE1 | ALB |
| TTR | KIF11 | MAPK14 | PTPN1 | F10 | ALB | KDR | KDR | AKR1B1 |
| MAPK14 | TTR | AKR1B1 | F2 | BACE1 | KDR | TYMS | GC | MAPK14 |
| RORA | ADH5 | RORA | TTR | PPARD | TYMS | PPARD | MAPK14 | ADAM17 |
| F10 | RORA | F10 | RORA | SRC | BACE1 | BACE1 | TYMS | TTR |
| GC | GC | PPARG | ADH5 | ALB | AR | AR | CDK2 | ESR1 |
| PTPN1 | DHODH | AKR1C3 | F10 | RORA | CDK2 | SRC | PPARD | F10 |
| TGFBR1 | CHEK1 | SEC14L2 | CHEK1 | TYMS | PPARD | F10 | AR | PARP1 |
| AKR1C3 | F10 | GC | AKR1C3 | AR | F10 | MMP8 | PGR | BCHE |
| DHODH | HSPA8 | DHODH | GC | FGFR1 | TNNC1 | CDK2 | TNNC1 | PGR |
| NR3C2 | MMP8 | ESR1 | DHODH | CDK2 | SRC | TGFBR1 | CES1 | ANXA5 |
| EGFR | MAPK14 | NR3C2 | TGFBR1 | PDE4B | CHEK1 | TNNC1 | FGFR1 | NR3C2 |
| MMP13 | EGFR | PTPN1 | TYMS | CHEK1 | PDE4B | CHEK1 | F10 | TNNC1 |
| TYMS | TYMS | CES1 | MMP8 | TNNC1 | NOS3 | PDE4B | TTR | PPARD |
| HSD11B1 | NR3C2 | EGFR | HSPA8 | MMP13 | NR3C2 | EGFR | PDE4B | GC |
| PPARD | BACE1 | NR1H4 | FABP4 | NOS3 | PDPK1 | MMP13 | CHEK1 | BACE1 |
| SEC14L2 | CDK2 | TYMS | NR3C2 | ANXA5 | ANXA5 | NOS3 | NOS3 | PPARG |
| TNNC1 | ESRRG | CDK2 | MAPK14 | EGFR | HSD11B1 | FGFR1 | SRC | AR |
| MMP8 | TNNC1 | TGFBR1 | EGFR | DHODH | HSP90AA1 | ANXA5 | NR3C2 | MMP8 |
| SRC | NOS3 | MMP13 | TNNC1 | HSP90AA1 | AKR1C1 | DHODH | ANXA5 | TGFBR1 |
| HSPA8 | NR1H4 | PPARD | CDK2 | THRB | DHODH | HSP90AA1 | EGFR | MMP13 |
| BACE1 | ESR1 | MMP8 | BACE1 | GSK3B | FGFR1 | MET | AKR1C1 | RORA |
| PDE4B | AR | TNNC1 | ESR1 | TTR | FABP4 | GSK3B | HSP90AA1 | PPP5C |
| ESRRG | ANXA5 | HSPA8 | ESRRG | MET | EGFR | TTR | HSD11B1 | DUSP6 |
| NR1H4 | HSD17B1 | SRC | CES1 | NR1H4 | GSK3B | FABP5 | IGF1R | SRC |
| NOS3 | KDR | BACE1 | NOS3 | HSD17B1 | DPP4 | HDAC8 | DHODH | TYMS |
| KDR | PPARD | ESRRG | NR1H4 | SOD2 | ADH5 | DPP4 | FABP5 | CDK2 |
| ANXA5 | SEC14L2 | KDR | SEC14L2 | HDAC8 | TTR | SOD2 | GSK3B | CES1 |
| PARP1 | FABP4 | PDE4B | MMP13 | PLA2G2A | HDAC8 | HSD17B1 | ADH5 | DHODH |
| CHEK1 | SULT2A1 | NOS3 | AR | PDE3B | HMGCR | PDE3B | DPP4 | HSD11B1 |

| | | | | | | | | |
|---|---|---|---|---|---|---|---|---|
| FGFR1 | MMP13 | CHEK1 | ANXA5 | CTSK | NR1H4 | NR1H4 | HMGCR | EGFR |
| SULT2A1 | HSD11B1 | AR | HSD11B1 | PDE4D | PLA2G2A | AURKA | HDAC8 | FGFR1 |
| HSD17B1 | AKR1C3 | FGFR1 | HSD17B1 | DPP4 | HSD17B1 | PDE4D | NR1H4 | MAPKAPK2 |
| MET | HSP90AA1 | HSD11B1 | KDR | SULT2A1 | PDE3B | AKR1C1 | PDE3B | KDR |
| ADH5 | EPHB4 | ANXA5 | PPARD | AKR1C1 | PDE4D | CTSK | PLA2G2A | PDE4B |
| MDM2 | TRAPPC3 | HSD17B1 | SULT2A1 | REN | REN | PLA2G2A | PDE4D | HSP90AA1 |
| TRAPPC3 | MET | MDM2 | TRAPPC3 | SORD | FABP5 | PTPN11 | HSD17B1 | SEC14L2 |
| EPHX2 | PDE3B | MET | HSP90AA1 | WAS | PTPN11 | WAS | REN | NOS3 |
| PCK1 | DPP4 | LTA4H | EPHB4 | PTPN11 | PNMT | PNMT | PNMT | MET |
| WAS | HDAC8 | ADH5 | DPP4 | FABP5 | TGFBR1 | REN | PTPN11 | SULT2A1 |
| EPHB4 | PLA2G2A | SULT2A1 | PDE3B | AURKA | SOD2 | SORD | SORD | CHEK1 |
| BLVRB | PDE4D | PTPN11 | HDAC8 | PNMT | SORD | ADK | ERBB4 | PTPN11 |
| ADH1C | PTPN11 | TRAPPC3 | MET | ESR2 | PLAU | ESR2 | CTSK | HDAC8 |
| PDE3B | SRC | LCK | PDE4B | SEC14L2 | CTSK | ADH5 | CASP3 | ADH5 |
| DPP4 | SORD | BLVRB | PLA2G2A | PIK3CG | CASP3 | ADH1C | PLAU | MDM2 |
| PTPN11 | PNMT | WAS | PDE4D | SYK | ERBB4 | SEC14L2 | SEC14L2 | BLVRB |
| HDAC8 | PDK2 | HDAC8 | PTPN11 | CASP3 | SEC14L2 | CASP3 | ESR2 | TRAPPC3 |
| GSK3B | HMGCR | PARP1 | AKR1C1 | ADH1C | SYK | SYK | SYK | DPP4 |
| PDE4D | PDE4B | EPHB4 | ESRRA | ITK | PDK2 | MDM2 | CYP2C9 | GSK3B |
| ESRRA | CTSK | PDE3B | THRB | MDM2 | ESR2 | PIK3CG | PDK2 | ESRRA |
| HSP90AA1 | AKR1C1 | ADH1C | SRC | ADH5 | RBP4 | SULT2A1 | RBP4 | HSD17B1 |
| RXRA | PARP1 | ESRRA | PNMT | PARP1 | THRB | ERBB4 | AKR1C3 | ERBB4 |
| PNMT | ESRRA | DPP4 | SORD | NR1I2 | CYP2C9 | ITK | MDM2 | PIK3CG |
| SOD2 | ADK | THRB | HMGCR | RBP4 | SULT2A1 | NR1I2 | PIK3CG | SOD2 |
| PIK3CG | EPHX2 | PDE4D | ADK | NQO1 | AKR1C3 | RBP4 | TGFBR1 | RXRA |
| SORD | F7 | GSK3B | PCK1 | CTNNA1 | MDM2 | GSTA1 | FABP3 | SYK |
| PLA2G2A | MMP2 | HSP90AA1 | PLAU | ERBB4 | FABP3 | PDK2 | NR1I2 | PLA2G2A |
| SYK | CASP3 | RXRA | FABP5 | ADK | MET | FABP3 | PDE5A | WAS |
| ADK | TTPA | SOD2 | REN | FABP3 | PIK3CG | AKR1C2 | STS | LCK |
| CTSK | ACE2 | PNMT | PARP1 | AKR1C2 | NR1I2 | CYP2C9 | THRA | ITK |
| AKR1C1 | TGFBR1 | SORD | PDK2 | RARA | RARG | FKBP1A | RARB | NR1H4 |
| REN | PDPK1 | FABP5 | EPHX2 | RARG | RARB | THRA | RARA | AKR1C1 |
| ESR2 | SHBG | ADK | RXRA | CA5A | RARA | RARB | RARG | ESR2 |
| AURKA | FABP3 | CTSK | CTSK | MPL | CA7 | RARA | CA5A | PGF |
| F7 | PLAU | PLA2G2A | CASP3 | MIF | CA6 | RARG | MPL | AKR1C3 |
| ERBB4 | FABP7 | ERBB4 | MMP2 | HSD11B1 | MIF | CA5A | TRPV1 | PDE4D |
| ITK | RXRA | EPHX2 | TTPA | CES2 | HTR1E | CA14 | MIF | PNMT |
| GSTA1 | HNF4G | REN | SHBG | CA7 | RELA | MIF | AHR | RBP4 |
| CASP3 | CYP2C8 | AKR1C1 | F7 | CA6 | GPR35 | ALOX15 | CES2 | CTNNA1 |
| TTPA | REN | AURKA | ACE2 | ALPL | HSD17B2 | CES1 | BCL2A1 | CASP3 |
| SHBG | GLO1 | SYK | FGFR1 | RARB | ALPL | CACNA1B | CA7 | FABP5 |
| HNF4G | FKBP1A | ESR2 | SYK | CNR2 | ALOX5 | CES2 | CA6 | REN |
| PPARA | FGFR1 | CASP3 | WAS | KIF11 | DUSP3 | CA7 | RAF1 | ADK |
| FABP3 | PYGL | F7 | PDPK1 | NR1H3 | STS | CA5B | ALOX15 | ESRRG |
| RBP4 | LTA4H | PIK3CG | FABP3 | AHR | THRA | CA4 | PLIN1 | PDE3B |
| MMP12 | GSK3B | PCK1 | GLO1 | GCGR | CA14 | CA6 | CA5B | ADH1C |
| LCK | PCK1 | TTPA | HNF4G | CES1 | AHR | MPL | CA14 | PCK1 |
| FGFR2 | NR1I2 | MMP12 | GSK3B | CA5B | CTDSP1 | AHR | GPR35 | THRB |
| CYP2C8 | NR1H3 | FGFR2 | CYP2C8 | AKR1C3 | CA4 | PTGS2 | PTGS1 | EPHX2 |
| GLO1 | FABP5 | SHBG | PYGL | CACNA1B | CYP2C19 | BCL2A1 | CA4 | FKBP1A |
| NR1H2 | THRA | FABP3 | FABP7 | PTGES | ABCG2 | KIF11 | PTGS2 | HMGCR |

| | | | | | | | | |
|---|---|---|---|---|---|---|---|---|
| PDK2 | THRB | HNF4G | GSTA1 | ALOX15 | CYP1A2 | PLIN1 | PLIN5 | SHBG |
| NQO1 | MCL1 | PDK2 | MMP12 | CA14 | PTGER2 | STS | CACNA1B | CTSK |
| FABP7 | CES1 | CYP2C8 | PPARA | S1PR2 | CA5A | CA2 | RELA | F7 |
| LTA4H | PTGES | GLO1 | FKBP1A | PTGS2 | TLR9 | CNR2 | PTGES | NR1H3 |
| FKBP1A | S1PR2 | RBP4 | NR1I2 | GPR35 | DRD1 | GPR35 | CASP9 | SORD |
| RARG | CES2 | RARG | THRA | THRA | S1PR2 | PTGS1 | HTR1E | CCNT1 |
| NR1I2 | CA7 | NQO1 | CA5A | HTR1E | ABCB1 | S1PR2 | CA13 | RARG |
| PYGL | CA6 | FKBP1A | ALOX15 | ALOX5 | CACNA1H | AKR1C3 | ALOX5 | NQO1 |
| SULT1E1 | S1PR4 | NR1I2 | S1PR5 | HSD17B2 | GALR3 | RXRB | MCL1 | JAK2 |
| AKR1C2 | S1PR5 | PYGL | S1PR2 | PLIN1 | APOBEC3G | RXRG | DUSP3 | LTA4H |
| CCNT1 | CA13 | AKR1C2 | CES2 | BCL2A1 | APOBEC3A | HSD17B3 | PTGER4 | AURKA |
| HMGCR | DUSP3 | GSTA1 | CA7 | PTGS1 | GRM2 | CASP9 | CA9 | PDK2 |
| THRA | CNR2 | CYP2C9 | CA13 | RELA | ADRA2B | CA1 | CA1 | PYGL |
| THRB | PLIN5 | ITK | CA5B | CA4 | HRH1 | RELA | RAC1 | NR1I2 |
| CNR2 | PTGDR2 | CCNT1 | CA4 | RXRB | MTNR1B | CA9 | DNMT1 | GSTA1 |
| S1PR5 | GPR35 | HMGCR | CA6 | CA13 | HRH2 | PLA2G1B | HSD17B2 | FABP3 |
| CES1 | S1PR3 | THRA | S1PR4 | CA2 | DRD5 | ALPL | SIRT1 | GLO1 |
| PTGES | CA4 | CA5A | CNR2 | RAC1 | DRD4 | DUSP3 | CYP2C19 | HNF4G |
| S1PR2 | ALOX15 | ALOX15 | MIF | STS | CYP19A1 | CTDSP1 | NR2F2 | FABP7 |
| CES2 | S1PR1 | S1PR5 | CDC25B | CYP2C19 | MPL | HSD11B1 | RIPK2 | THRA |
| CA7 | CA5A | S1PR2 | CA12 | PLIN5 | TRPV1 | RAC1 | ABCG2 | CES2 |
| CA6 | CA12 | CES2 | CA14 | CASP9 | ADRA2A | CYP2C19 | CTDSP1 | CDC25B |
| S1PR4 | CNR1 | CA7 | DUSP3 | MCL1 | MAOA | PTPN1 | ADIPOQ | CACNA1B |
| CA13 | PTGER2 | CA13 | SIRT2 | CNR1 | HTR2A | CA13 | PLAT | ALOX15 |
| CA4 | CA5B | CA5B | PLA2G1B | S1PR4 | DRD2 | HTR1E | TNFRSF1A | MIF |
| MGLL | ALOX5 | CA4 | MAOA | MMP7 | NOS2 | PLIN5 | NFE2L2 | S1PR4 |
| MIF | BCL2A1 | CA6 | PTGES | NOS2 | MTNR1A | SIRT2 | TFF1 | ANPEP |
| CDC25B | ABCG2 | S1PR4 | S1PR1 | PLAT | CHRM4 | CA12 | CCL2 | CA7 |
| NR1H3 | HTR1E | CNR2 | MGLL | ADIPOQ | DRD3 | RXRA | MMP7 | CAPN1 |
| ALOX15 | MIF | MIF | CA9 | CCL2 | HTR5A | THRB | PROS1 | MCL1 |
| CA5B | AHR | CDC25B | LTB4R | TNFRSF1A | SLC6A3 | PTGES | | GCGR |
| MCL1 | TOP2A | CA12 | GPR35 | HMOX1 | SLC6A2 | ADIPOQ | | CA6 |
| TAAR1 | NOS2 | CA14 | MCL1 | CYP3A7 | SLC6A4 | PLAT | | FOLH1 |
| S1PR1 | MMP7 | DUSP3 | CTDSP1 | PGR | TAAR1 | TNFRSF1A | | PLA2G1B |
| CA12 | ADIPOQ | SIRT2 | RARB | CD86 | CES2 | PGR | | PTGER2 |
| PTGER2 | HMOX1 | PLA2G1B | BCL2A1 | CD14 | HTR2C | TFF1 | | ADIPOQ |
| LTB4R | PLAT | MAOA | PLIN5 | | CYP2D6 | CCL2 | | CCL2 |
| CA5A | CCL2 | PTGES | CA1 | | CHRM3 | NFE2L2 | | ELAVL1 |
| CNR1 | ABCA1 | S1PR1 | CYP2C19 | | ADRA2C | HMOX1 | | PLAT |
| PTGDR2 | GAPDH | MGLL | TOP2A | | CHRM1 | RARA | | GH1 |
| ALOX5 | | CA9 | MMP7 | | HDAC6 | GAPDH | | HMOX1 |
| DUSP3 | | LTB4R | PROS1 | | RXRG | MMP7 | | NGFR |
| GPR35 | | GPR35 | HBA1 | | P2RX7 | NOS2 | | PROC |
| CA14 | | MCL1 | NOS2 | | HRH3 | PROS1 | | MMP7 |
| ALPL | | CTDSP1 | ADIPOQ | | ADIPOQ | CASP8 | | NOS2 |
| PPARG | | RARB | PLAT | | PLAT | | | |
| PLIN5 | | BCL2A1 | HMOX1 | | HMOX1 | | | |
| SIRT2 | | PLIN5 | GAPDH | | TNFRSF1A | | | |
| NOS2 | | CA1 | | | CCL2 | | | |
| MMP7 | | CYP2C19 | | | CYP3A7 | | | |
| TOP2A | | MMP7 | | | MMP7 | | | |

| | | | | | | | | |
|---|---|---|---|---|---|---|---|---|
| MMP2 | | TOP2A | | | NOS2 | | | |
| VIM | | NOS2 | | | | | | |
| ADIPOQ | | MMP2 | | | | | | |
| CCL2 | | HBA1 | | | | | | |
| ABCA1 | | PROS1 | | | | | | |
| HMOX1 | | PPARA | | | | | | |
| GAPDH | | ADIPOQ | | | | | | |
| GH1 | | PLAT | | | | | | |
| TIMP1 | | CCL2 | | | | | | |
| | | GAPDH | | | | | | |
| | | HMOX1 | | | | | | |
| | | ABCA1 | | | | | | |
| | | PGR | | | | | | |
| | | TIMP1 | | | | | | |
| | | RARA | | | | | | |

| 5h | 5e | 5f |
|---|---|---|
| MAPK8 | MAPK10 | MAPK8 |
| NR1I3 | MAPK8 | NR1I3 |
| MAPK10 | MMP3 | MAPK10 |
| MMP3 | GSTP1 | MMP3 |
| KIF11 | NR1I3 | KIF11 |
| CFB | MAPK1 | CFB |
| LCN2 | BMP2 | LCN2 |
| BMP2 | KIF11 | BMP2 |
| MAPK1 | CFB | MAPK1 |
| STS | PIM1 | GSTP1 |
| CASP7 | MAOB | PIM1 |
| PIM1 | STS | CASP7 |
| GSTP1 | CASP7 | STS |
| PTPN1 | F2 | APOA2 |
| APOA2 | AKR1B1 | CCNA2 |
| CCNA2 | APOA2 | F2 |
| AKR1C2 | CCNA2 | MAOB |
| MAOB | PTPN1 | CA2 |
| F2 | PDPK1 | AKR1C2 |
| AKR1B1 | AKR1C2 | AKR1B1 |
| CA2 | CA2 | ALB |
| ALB | ALB | MAPK14 |
| PPARG | ADAM17 | ADAM17 |
| MAPK14 | TTR | PPARG |
| ADAM17 | MAPK14 | BCHE |
| ESR1 | PARP1 | PGR |
| BCHE | F10 | ESR1 |
| PGR | ESR1 | F10 |
| CHEK1 | BCHE | CHEK1 |
| ANXA5 | PGR | NR3C2 |
| F10 | ANXA5 | TNNC1 |
| NR3C2 | TGFBR1 | ANXA5 |
| RORA | NR3C2 | PPARD |
| TNNC1 | PPARD | RORA |
| BACE1 | TNNC1 | BACE1 |
| MMP13 | MMP13 | TGFBR1 |
| GC | MMP8 | MMP13 |
| TYMS | BACE1 | TYMS |
| PPP5C | HSD11B1 | AR |
| TGFBR1 | GC | PPP5C |
| DUSP6 | RORA | ADH5 |
| ADH5 | AR | DUSP6 |
| AR | PPARG | GC |
| DHODH | PPP5C | DHODH |
| EGFR | DUSP6 | HSD11B1 |
| PPARD | TYMS | CES1 |
| CDK2 | SRC | CDK2 |
| NOS3 | CDK2 | EGFR |
| HSD11B1 | DHODH | PDPK1 |
| SRC | FGFR1 | SRC |

| | | |
|---|---|---|
| PDPK1 | EGFR | NOS3 |
| SULT2A1 | KDR | HDAC8 |
| HDAC8 | MAPKAPK2 | MAPKAPK2 |
| HSP90AA1 | PDE4B | KDR |
| PDE4B | HSP90AA1 | SULT2A1 |
| KDR | SEC14L2 | PDE4B |
| ERBB4 | NOS3 | PARP1 |
| AKR1C1 | CES1 | HSP90AA1 |
| BLVRB | CHEK1 | FGFR1 |
| DPP4 | SULT2A1 | ERBB4 |
| MET | MET | AKR1C1 |
| PNMT | PTPN11 | MDM2 |
| GSK3B | HDAC8 | PCK1 |
| FGFR1 | MDM2 | BLVRB |
| TTR | ADH5 | DPP4 |
| NR1H3 | BLVRB | TTR |
| ESRRA | ERBB4 | SEC14L2 |
| PTPN11 | PCK1 | PNMT |
| PLA2G2A | DPP4 | NR1H3 |
| HSD17B1 | TRAPPC3 | MET |
| FABP5 | GSK3B | PTPN11 |
| HMGCR | LCK | GSK3B |
| SEC14L2 | PIK3CG | ESRRA |
| AKR1C3 | SYK | PLA2G2A |
| PDE4D | SOD2 | HMGCR |
| PGF | WAS | HSD17B1 |
| LCK | HSD17B1 | RXRA |
| CTNNA1 | ESRRA | AKR1C3 |
| MMP8 | PNMT | PDE4D |
| PDE3B | PLA2G2A | PGF |
| PARP1 | FABP5 | MMP8 |
| ESRRG | ITK | WAS |
| WAS | AKR1C1 | CTNNA1 |
| PCK1 | AKR1C3 | FABP5 |
| ESR2 | PDE4D | LCK |
| TRAPPC3 | PGF | PDE3B |
| CASP3 | RBP4 | ACE2 |
| SHBG | CTNNA1 | ESRRG |
| FKBP1A | ESR2 | TRAPPC3 |
| ACE2 | CASP3 | CASP3 |
| NR1H2 | ESRRG | FKBP1A |
| THRB | ADK | ESR2 |
| EPHX2 | ADH1C | NR1H4 |
| NR1H4 | REN | EPHX2 |
| CCNT1 | PDE3B | NR1H2 |
| SORD | THRB | SHBG |
| RXRA | FKBP1A | CCNT1 |
| FABP7 | EPHX2 | SORD |
| CTSK | HMGCR | JAK2 |
| JAK2 | CTSK | CTSK |
| F7 | F7 | CYP2C9 |

| | | |
|---|---|---|
| REN | NR1H4 | FABP7 |
| CYP2C9 | NR1H3 | REN |
| HNF4G | LTA4H | LTA4H |
| PDK2 | SHBG | PYGL |
| PYGL | RXRA | F7 |
| LTA4H | CCNT1 | FABP3 |
| FABP3 | SORD | HNF4G |
| TTPA | AURKA | PLAU |
| HPN | NQO1 | RBP4 |
| THRA | FABP7 | PDE5A |
| CES2 | GLO1 | TTPA |
| MCL1 | RARG | HPN |
| CDC25B | PYGL | THRA |
| ALOX15 | JAK2 | THRB |
| VCAM1 | HNF4G | ALOX15 |
| MDM2 | HCK | CES2 |
| FOLH1 | FABP3 | PTPN1 |
| PLA2G1B | NR1H2 | FOLH1 |
| CA7 | GSTA1 | CACNA1B |
| MIF | TTPA | ECE1 |
| CACNA1B | THRA | CA6 |
| PTGER2 | FOLH1 | MCL1 |
| ANPEP | ALOX15 | ANPEP |
| ADIPOQ | CACNA1B | CDC25B |
| CCL2 | CES2 | PLA2G1B |
| HMOX1 | CDC25B | CA7 |
| ELAVL1 | CA6 | SIRT1 |
| GH1 | MIF | MIF |
| PLAT | PLA2G1B | HDAC3 |
| NGFR | CA7 | CA13 |
| MMP7 | CA5A | BCL2A1 |
| | ANPEP | CA4 |
| | CA4 | ADIPOQ |
| | MMP2 | HMOX1 |
| | MMP1 | PLAT |
| | ECE1 | NGFR |
| | PTPRC | CCL2 |
| | MMP9 | MMP7 |
| | S1PR2 | |
| | MCL1 | |
| | ADIPOQ | |
| | PLAT | |
| | CCL2 | |
| | HMOX1 | |
| | NGFR | |
| | CD36 | |
| | GAPDH | |
| | GH1 | |
| | RARA | |
| | MMP7 | |
| | CASP8 | |

| | | |
|---|---|---|
| | NOS2 | |
| | | |
| | | |
| | | |
| | | |
| | | |
| | | |
| | | |
| | | |
| | | |
| | | |
| | | |
| | | |
| | | |
| | | |
